# Catalase and Ascorbate Peroxidase in Euglenozoan Protists

**DOI:** 10.3390/pathogens9040317

**Published:** 2020-04-24

**Authors:** Ingrid Škodová-Sveráková, Kristína Záhonová, Barbora Bučková, Zoltán Füssy, Vyacheslav Yurchenko, Julius Lukeš

**Affiliations:** 1 Institute of Parasitology, Biology Centre, Czech Academy of Sciences, 370 05 České Budějovice (Budweis), Czech Republic; kika.zahonova@gmail.com; 2 Faculty of Natural Sciences, Comenius University, 841 04 Bratislava, Slovakia; barbora@bucko.sk; 3 Faculty of Science, Charles University, BIOCEV, 128 00 Prague, Czech Republic; zoltan.fussy@gmail.com; 4 Life Science Research Centre, Faculty of Science, University of Ostrava, 710 00 Ostrava, Czech Republic; vyacheslav.yurchenko@osu.cz; 5 Martsinovsky Institute of Medical Parasitology, Tropical and Vector Borne Diseases, Sechenov University, 119435 Moscow, Russia; 6 Faculty of Sciences, University of South Bohemia, 370 05 České Budějovice (Budweis), Czech Republic

**Keywords:** Euglenozoa, ascorbate peroxidase, catalase, enzymatic activity, phylogeny

## Abstract

In this work, we studied the biochemical properties and evolutionary histories of catalase (CAT) and ascorbate peroxidase (APX), two central enzymes of reactive oxygen species detoxification, across the highly diverse clade Eugenozoa. This clade encompasses free-living phototrophic and heterotrophic flagellates, as well as obligate parasites of insects, vertebrates, and plants. We present evidence of several independent acquisitions of CAT by horizontal gene transfers and evolutionary novelties associated with the APX presence. We posit that Euglenozoa recruit these detoxifying enzymes for specific molecular tasks, such as photosynthesis in euglenids and membrane-bound peroxidase activity in kinetoplastids and some diplonemids.

## 1. Introduction

Aerobic metabolism is associated with the undesirable production of reactive oxygen species (ROS) due to the leakage of electrons to molecular oxygen [1]. The ROS molecules include free radicals, such as superoxide anion (O_2_^●−^) or hydroxyl radical (^●^OH), and non-radical molecules, e.g., hydrogen peroxide (H_2_O_2_) [2]. In the eukaryotic cell, respiration in mitochondria and photosynthesis in plastids are the main ROS producers. However, ROS may accumulate as a by-product in any cell compartment where aerobic metabolism occurs. While molecular oxygen shows relatively low reactivity towards most cellular components, its partially reduced forms are much more reactive. Oxidative damage of proteins, lipids, and nucleic acids happens when the level of ROS exceeds the physiological threshold under oxidative stress conditions [3]. Hydrogen peroxide is often used as a second messenger in cell signaling pathways and its accumulation has long been documented to play an important role in mediating programmed cell death—apoptosis or (at very high concentrations) necrosis [4]. Hence, both the production and removal of ROS must be strictly controlled. This is why enzymatic and non-enzymatic mechanisms for ROS detoxification are employed by virtually every cell [5].

The family of enzymatic antioxidants comprises catalase (CAT), ascorbate peroxidase (APX), superoxide dismutase (SOD), glutathione peroxidase (GPX), and peroxiredoxin (PrxR) [6,7,8]. Both CAT and APX are heme-containing enzymes widely distributed among aerobes. As such, the APX activity was documented in higher plants and a range of protist lineages, including chlorophytes, rhodophytes, stramenopiles, and euglenozoans [9,10,11]. The structure and function of APX have been well described in plants, which usually carry several isoforms [12]. While APX requires ascorbate for ROS detoxification, with four reactions of the ascorbate–glutathione cycle necessary for ascorbate regeneration, CAT represents a system for direct ROS dissociation by conversion of H_2_O_2_ molecules into water and molecular oxygen. This enzyme requires one molecule of H_2_O_2_ to bind at the CAT active site in order to generate a reaction intermediate that binds the second molecule of H_2_O_2_ [13]. The affinities of APX and CAT for H_2_O_2_ are in µM and mM ranges, respectively, reflecting that they belong to two fundamentally different classes of peroxidases [14]. Having a low affinity to H_2_O_2_, CAT is most effective at high concentrations of peroxide [15]. 

ROS scavenging systems are classified according to their subcellular localization, which is primarily determined by the organelle-specific targeting signals found in the N- and C-termini of the corresponding proteins. Soluble forms are found in the cytosol, mitochondria, and plastids, while membrane-bound isoforms are found in microbodies (including peroxisomes and glyoxysomes) and plastid thylakoids [16,17,18]. Usually, multiple systems are present in a given cellular compartment and cooperate to scavenge for ROS [19]. CAT occurs in either soluble or membrane-bound forms, and is typically localized in the peroxisomes and mitochondria, where H_2_O_2_ production is most significant [20].

Kinetoplastids and euglenids possess yet another mechanism for scavenging ROS centered on the glutathione analog trypanothione, that is unique to these protists [21]. The trypanothione system reduces H_2_O_2_ via tryparedoxin and tryparedoxin peroxidase. Since CAT and the selenium-containing GPX are absent in the kinetoplastid flagellates (for exceptions, see below), trypanothione is particularly important in this group of organisms [22]. For the same reasons, APX appears to be a key enzyme for redox homeostasis in these flagellates [23]. In addition, trypanosomatids also lack the whole thioredoxin/thioredoxin reductase pathway, and it was proposed that trypanothione actually substitutes it. However, in the absence of CAT, trypanothione and thioredoxin systems co-exist in *Euglena gracilis*, indicating that these systems are not entirely redundant [24,25,26]. 

It was shown that, under stress, plants boost the activity of all of their ROS scavenging enzymatic systems, namely CAT, APX, SOD, GPX, and PrxR [16]. Interestingly, an increase in the APX activity can compensate for the loss of the CAT activity in plants and prevent the accumulation of intracellular ROS, suggesting the functional overlap of both systems [5]. Indeed, in tobacco, the CAT and APX machineries are (to some extent) functionally redundant, as one can compensate for the lack of another [27]. Given the complex pattern of antioxidant systems in the euglenozoan protists, we performed detailed phylogenetic and functional analyses of their APX proteins, dissected their co-occurrence with CAT, and identified multiple acquisitions of these nearly ubiquitous enzymes from unrelated sources.

Euglenozoa are as diverse as can be, and they comprise free-living, parasitic, and photosynthetic species, having clinical, economical, and environmental importance. Yet, how euglenozoans actually cope with ROS is still poorly understood. Given the complex pattern of antioxidant systems in euglenozoan protists, we performed phylogenetic and biochemical analyses of their APX and CAT to shed more light on the importance of these nearly ubiquitous enzymes.

## 2. Results

### 2.1. Euglenozoans Encode Unique HPXs and CAT of Different Origins

**Figure 1:**
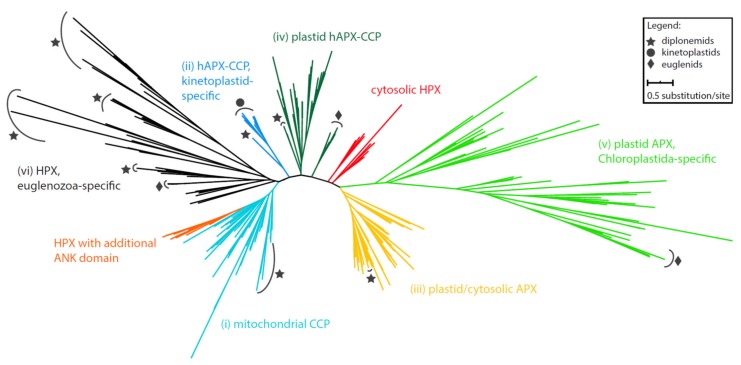
Maximum-likelihood phylogeny of heme peroxidases possessing APX domains in Euglenozoa. Taxa representing euglenozoan sequences are marked by symbols according to the graphical legend. Full tree in Newick format can be found in File S1.

An extensive phylogenetic analysis of the APX domain-containing sequences revealed the subdivision of sequences derived from Euglenozoa into several clades (Figure 1; File S1). They are well-separated from heme (HPX) and cytochrome *c* peroxidases (CCP), suggesting a sub-functionalization of the HPX superfamily early in the eukaryotic evolution. All studied sequences can be divided into the following clades: (i) diplonemid peroxidases, which are invariably predicted as mitochondrion-localized (in turquoise) (Supplementary Table S1), form a moderately supported sister lineage to mitochondrial CCPs; (ii) a sequence from the diplonemid *Lacrimia lanifica* forms a sister branch to the kinetoplastid-specific hybrid APX-CCP proteins (hAPX-CCPs, in blue). While the kinetoplastid hAPX-CCP orthologues carry a mitochondrial targeting signal, the *L. lanifica* sequence possesses a peroxisomal targeting signal (Supplementary Table S1), which should navigate the corresponding protein to the glycosomes [28]; (iii) APXs of the diplonemids *Artemidia motanka*, *Namystynia karyoxenos*, and *Sulcionema specki* are nested inside the plastid/cytosolic APX clade along with various algae, parasitic chytridiomycota, and filter-feeding choanoflagellates, all of which carry a PTS2 signal, strongly indicating their glycosomal localization (in bright orange); (iv) a clade, consisting solely of sequences from the diplonemids *A. motanka* and *N. karyoxenos*, branches inside the plastid hAPX-CCPs cluster, close to the plastid-targeted euglenid APX (in dark green). Consistent with the absence of plastid in diplonemids, their APXs are predicted to be cytosolic (Supplementary Table S1); (v) a small clade contains an additional APX homolog from the plastid-carrying euglenids, otherwise specific for Chloroplastida (in light green); (vi) all remaining 29 diplonemid and six euglenid sequences constitute five independent clades that are unrelated to known APX sequences (in black). In the absence of an appropriate reference ortholog, we cannot infer their function, and thus we denoted them as Euglenozoa-specific HPX. We predict these proteins to be targeted to a range of cellular compartments, including mitochondria, glycosomes, and the secretory organelles (Supplementary Table S1).

**Figure 2:**
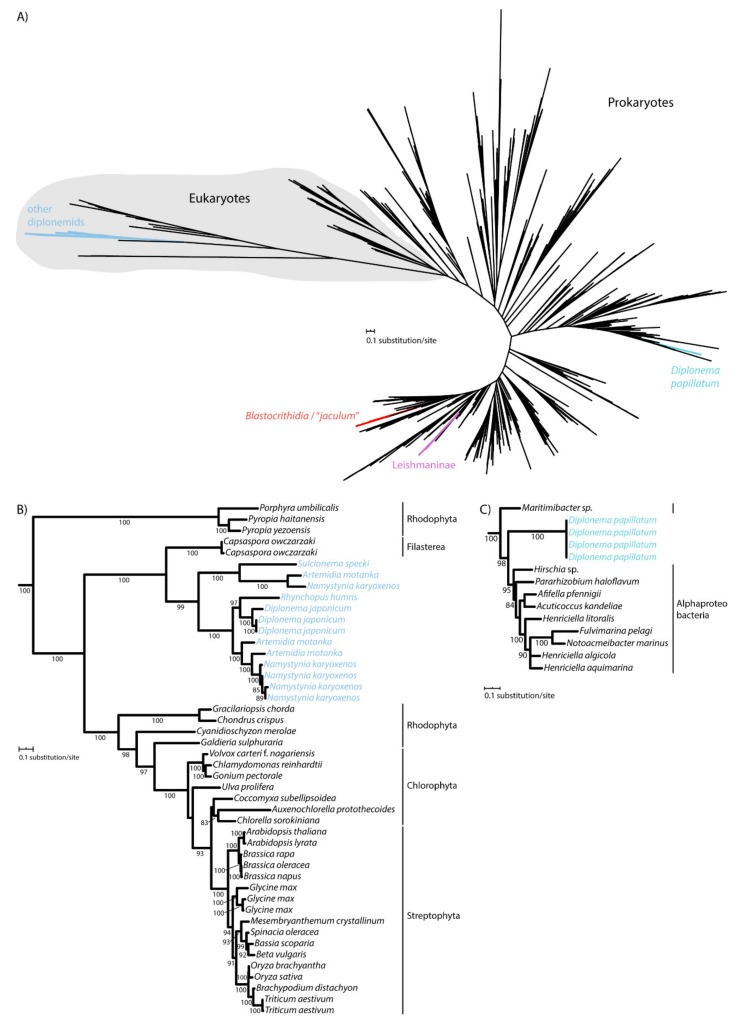
Maximum-likelihood phylogeny of catalases in Euglenozoa. (**A**) Unrooted tree showing ancestral origin of diplonemid CAT branching within eukaryotes and three horizontal gene transfer events in *Blastocrithidia*/“*jaculum*”, Leishmaninae and *D. papillatum*. Full tree in Newick format can be found in File S2. (**B**) Subtree showing the ancestral diplonemid CAT. (**C**) Subtree showing the *D. papillatum* CAT related to alpha-proteobacteria. UFBoot support values are shown when ≥ 80.

It was previously shown that kinetoplastid flagellates acquired their CAT enzyme at least two times independently from bacteria. As revealed by the phylogenetic analysis, CAT of Leishmaniinae and the *Blastocrithidia*/“*jaculum*” clade derive from different bacterial groups [29,30], and this was confirmed here using a larger dataset (Figure 2A; File S2). While euglenids, studied so far, do not encode CAT [11,31], we wondered whether the same pattern applies to diplonemids, which constitute a sister clade to kinetoplastids [32,33]. For this purpose, we took advantage of the transcriptomic data derived from the axenic cultures of several diplonemid species [26]. As supported by maximum likelihood, the CAT sequences of *Diplonema japonicum*, *N. karyoxenos*, *Rhynchopus humris*, *A. motanka*, and *S. specki* are nested within eukaryotes with maximum support, consistent with the ancestral origin of their CAT (Figure 2B). However, yet another diplonemid, *Diplonema papillatum*, has apparently gained its CAT by horizontal gene transfer from an alpha-proteobacterium (Figure 2A,C). Hence, the inspected euglenozoans have acquired CAT from at least three distinct bacterial sources, while the genes of the majority of studied diplonemids are clearly of eukaryotic origin. This shows an unusual propensity of this group of protists to functionally replace CAT with homologs from bacteria that they likely prey upon.

### 2.2. APX Activity Widely Varies Among Species

**Figure 3:**
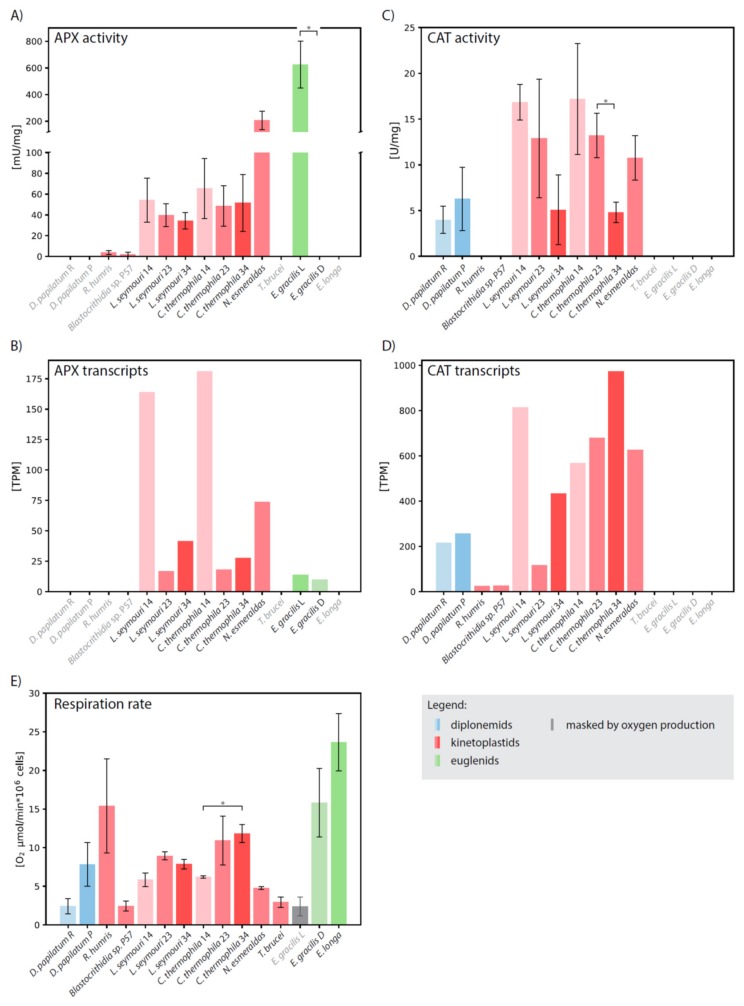
Biochemical and transcriptomic analysis. Comparison of (**A**) APX and (**C**) CAT activities, (**B**) APX and (**D**) CAT expression levels, and (**E**) oxygen uptake in *Diplonema papillatum* cultivated in nutrient-rich (R) and nutrient-poor (P) medium, *Rhynchopus humris*, *Blastocrithidia* sp. P57, *Leptomonas seymouri* cultivated at 14 °C (14), 23 °C (23) and 34 °C (34), *Crithidia thermophila* cultivated at 14 °C (14), 23 °C (23) and 34 °C (34), *Novymonas esmeraldas*, *Trypanosoma brucei*, *Euglena gracilis* cultivated in light (L) and dark (D), and *Euglena longa*. Species names in grey denote organisms, in which corresponding enzyme was not identified. Activity U is defined as the amount of the enzyme which catalyzes the conversion of 1 μmol of ascorbate (APX) or H_2_O_2_ (CAT) per 1 min. Each experiment was performed in two biological replicates. Statistical significance of differences between organisms was evaluated by an unpaired *t*-test. * statistically significant (*p* < 0.05). Note that respiration value in light-grown *E. gracilis* is masked by photosynthetic oxygen consumption (grey bar).

To corroborate computational results by biochemical evidence, we measured the CAT and APX activities separately in total cell lysates. In a good correlation, *D. papillatum* lacks both APX genes and the corresponding biochemical activity (Figure 3). 

Surprisingly, in *R. humris* and *Blastocrithidia* sp. P57 very low APX activity was detected, despite the fact that both species apparently lack the corresponding gene. *Leptomonas seymouri*, *Crithidia thermophila* (both cultivated at a standard temperature of 23 °C), and *Novymonas esmeraldas* possess the kinetoplastid-specific hAPX-CCP, and consistently, specific activities of 39 ± 10, 48 ± 19, and 206 ± 69 mU/mg, respectively, were documented in these species. (Figure 3A; Supplementary Table S2). It should be noted that the activity of *N. esmeraldas* APX is comparable to that of the full-length APX of *Leishmania major* [34]. Although the APX activity changed when *L. seymouri* and *C. thermophila* were shifted to 14 °C and 34 °C, the limit temperatures at which both organisms are able to grow, the change was not statistically significant (Figure 3A). Interestingly, gene expression followed the same pattern in both species with the highest number of transcripts at 14 °C and their decrease with elevated temperature (Figure 3B; Supplementary Table S2). 

When *E. gracilis* is grown under light conditions, promoting photosynthesis, it exhibits high APX activity of 625 ± 176 mU/mg (Supplementary Table S2), which is still 4-times lower than the activity reported for the pea plastids [35]. When the *E. gracilis* culture was transferred from light to dark, its color changed from green to pale reddish and the photosynthetic activity ceased. Although a low amount of APX transcripts were still present in these conditions, the enzymatic activity dropped below the limit of detection (Figure 3B; Supplementary Table S2). Consistent with the localization of APX in the plastid, neither the dark-grown *E. gracilis* nor the non-photosynthetic *Euglena longa* exhibit any APX activity (Figure 3A,B; Supplementary Table S2).

### 2.3. The Catalytic Center of APX is Altered in Euglenozoans

**Figure 4:**
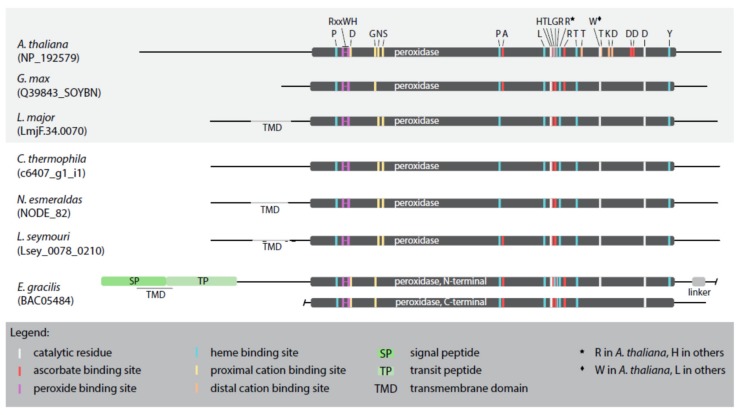
Schematic alignment of APX sequences from selected taxa. APXs from organisms studied previously are boxed in grey. Important amino acid residues are highlighted by different colors explained in the graphical legend.

The primary APX sequences from selected representatives, namely the plastid-bearing *Arabidopsis thaliana*, *Glycine max*, and *E. gracilis*, and the plastid-lacking *L. major*, *L. seymouri*, *C. fasciculata*, and *N. esmeraldas* possess the domains required for the H_2_O_2_-reducing APX activity (Figure 4; Supplementary Figure S1). 

The critical residues that coordinate binding of H_2_O_2_ by APX are R^158^, W^161^, and H^162^ (numbering according to the alignment in Supplementary Figure S1) [36], with the active site composed of the catalytic triad H^293^, W^325^, and D^354^ [37]. However, in the catalytic center of the studied APX sequences W^325^ is invariably replaced with L^325^, except for the APX of *A. thaliana* (Supplementary Figure S1). Furthermore, we have identified residues critical for the heme and ascorbate binding, the number of which being species-specific (Figure 4; Supplementary Figure S1). Regarding the heme binding residues, in all analyzed sequences (except for *A. thaliana*), H^299^ substitutes R^299^, similarly to the multiple isoforms of cytosolic APX [36]. Surprisingly, when compared to the model *A. thaliana* APX sequence, the proximal and distal cation-binding sites have been significantly changed or lost altogether, respectively, in all inspected euglenozoans and *G. max* (Figure 4; Supplementary Figure S1).

### 2.4. CAT Activity is Temperature-Dependent

In consonance with the absence of the CAT-encoding gene in euglenids [25] and *T. brucei* [38], we did not detect any CAT activity in these species (Figure 3C; Supplementary Table S2). *N. Esmeraldas* exhibited the CAT activity of 10 ± 2.4 U/mg, but with the employed methodology we failed to detect any activity in *Blastocrithidia* sp. P57 and *R. humris*, despite the fact that corresponding transcripts were expressed (Figure 3C,D; Supplementary Table S2). While the rich and poor cultivation medium has a significant impact on the metabolism of *D. papillatum*, the CAT activity remained stable under different conditions (Figure 3C; Supplementary Table S2).

Recently, it has been demonstrated that the human CAT and its orthologue from a monoxenous (insect-hosts only) trypanosomatid *C. fasciculata* have very different temperature optima [39]. In order to investigate the thermal properties of euglenozoan CAT in more detail, we examined its activity in *C. thermophila* and *L. seymouri*, both at their optimal cultivation temperature of 23 °C, as well as at 14 °C and 34 °C. These monoxenous species were selected because they have the highest expression and activity of CAT among the studied euglenozoans (Figure 3C,D; Supplementary Table S2), and are also thermo-tolerant, being able to withstand temperature changes [40,41]. In both species, the elevated temperature lowered CAT activity (Figure 3C). Nevertheless, the increased temperature caused a mild increase (1.5- and 3.7-times in *C. thermophila* and *L. seymouri*, respectively) in the mRNA level of CAT. The resulting pattern of CAT activity and transcription is rather complex. In both species, lower temperature generally resulted in increased CAT activity (Figure 3C), although the correlation between temperature and transcription levels was different for *C. thermophila* and *L. seymouri* (Figure 3D).

### 2.5. Respiration Rate Does Not Correlate with CAT and APX Activities

We assumed that organisms with high oxygen uptake require highly active systems for effective ROS detoxification. If so, CAT was the best candidate for this role, since its activity is not linked to any other system and it can provide direct and rapid reduction of H_2_O_2_. However, we did not find any correlation between the presence or activity of the CAT or APX detoxification systems and the rate of respiration (Figure 3E; Supplementary Table S2). Although it has the highest oxygen uptake, *E. longa* encodes neither CAT nor APX in its transcriptome (presumed to be highly representative [42]), and, correspondingly, their activities were lacking (Figure 3). Consistently, the dark-grown *E. gracilis* does not display any APX activity, with its respiration rate only slightly lower than that of *E. longa* (Figure 3E). Since oxygen consumption was masked by photosynthesis in the illuminated *E. gracilis* with fully developed plastids, we could not properly evaluate respiration in this case. The CAT activity of *D. papillatum* was not significantly influenced by the cultivation conditions (rich versus poor medium), even with increased respiration rate in the latter (Figure 3E). The respiration rate of thermostable *C. thermophila* and *L. seymouri* was lowest at 14 °C, however the enzymatic activities were highest (Figure 3A,C,E).

## 3. Discussion

The complex phylogenetic pattern, diversity, and distribution of CAT and APX in euglenozoans testify to their importance for these protists. Indeed, *E. gracilis* contains a photosynthesis-specific APX shared with other phototrophic euglenophytes, along with a putative plastidial APX acquired from and limited to Chloroplastida (Figure 1). Moreover, both diplonemids and euglenids encode a novel clade of peroxidases with an unknown function, while most kinetoplastids share a unique hAPX-CCP enzyme exhibiting both the APX and CCP activities [43]. Surprisingly, the kinetoplastid *Blastocrithidia* sp. P57 and the diplonemid *R. humris* apparently lack hAPX-CCP, so the low APX activity in both species must be assigned to another, possibly horizontally transferred, yet unidentified enzyme. Another such example is CAT in *D. papillatum* that was gained horizontally from an alpha-proteobacterium (Figure 2C). The distribution of APX and CAT in diplonemids is best explained by a scenario, in which the predecessor of these marine protists lacked both enzymes, which were reacquired by horizontal gene transfer from either prokaryotic or eukaryotic sources (Figure 1 and Figure 2). The importance of possessing such detoxifying systems is highlighted by the fact that this has occurred independently in several euglenozoan lineages. 

For a long time, APX was considered characteristic for photosynthetic organisms, while CAT was thought to be ubiquitous in the aerobic systems [44]. However, the distribution of these enzymes in euglenozoans challenges both claims. Evidence for the presence of APX and prominent absence of CAT in *Trypanosoma cruzi* and *Leishmania major* suggest that the former contributes to the ROS scavenging also in these parasites [34]. The absence of CAT in certain parasitic trypanosomatids is likely due to an adaptation to their dixenous (two-hosts) lifestyle, as the transition in the development from the insect to the mammalian stages of *T. brucei* and *Leishmania* spp. seem to rely on H_2_O_2_ production [39,45,46]. However, the lack of CAT in dixenous kinetoplastids is not universal, as exemplified by *Trypanoplasma borelli* harboring a glycosomal CAT [47].

CAT and APX complement each other’s function in tobacco [27], so to clarify the evolutionary context of their functional differentiation in euglenozoans, we searched for the CAT and APX sequences in genomes and/or transcriptomes of representatives, for which such data are available. From these organisms, only *T. brucei*, *E. gracilis*, and *E. longa* lack both the CAT genes and corresponding activity (Figure 3C,D). However, despite the presence of CAT transcripts in *R. humris* and *Blastocrithidia* sp. P57, respective enzymatic activity was below our detection limit (Figure 3C), which is not consistent with the detection of low CAT activity via heme-dependent oxygen production [29]. Our methodology is based on a spectrophotometric measurement of a decrease in H_2_O_2_ (for CAT) and ascorbate (for APX) levels. Although the sensitivity of oxygen detection appears to be significantly higher than that for the spectrophotometric detection of H_2_O_2_ reduction, we used the latter method in order to have consistent comparison for the CAT and APX enzymes. The kinetic parameters of CAT suggest yet another explanation for the lack of measurable activity in *R. humris* and *Blastocrithidia* sp. P57. The low affinity of CAT to H_2_O_2_ implies that it is responsible for the removal of excessive ROS when their concentration is high, while high-affinity APX modulates low concentration of ROS, necessary for cell signaling [14]. Thus, it is plausible that under the applied experimental setup *R. humris* and *Blastocrithidia* sp. P57 were not exposed to the conditions in which ROS would exceed a threshold and upregulate CAT activity. 

Since at least *E. longa* and *T. brucei* have neither CAT nor APX activity, the plant-like pattern with APX complementing the lack of CAT does not apply to the euglenozoan protists. In *E. gracilis*, a high APX activity was limited to the phototrophic growth conditions and, supposedly, the plastid. The corresponding APX contains two homologous catalytic domains, forming an intramolecular dimeric structure and a class II plastid-targeting bipartite sequence [48]. Previously, the APX activity was demonstrated to be cytosolic [48], which is most likely an artifact of the procedure, given the presence of the encoded targeting sequence and recent proteomic evidence [49]. We propose that the high APX activity in *E. gracilis*, comparable with that in plant plastids [35], mainly mitigates photosynthetic ROS production in plastids, rather than amends the absence of CAT. Furthermore, the APX activity in plastid-lacking protists, assayed herein, is 3–18-times lower when compared to the phototrophic *E. gracilis*, suggesting that its expression in these species does not meet conditions where high amounts of ROS need to be combatted.

The catalytic properties of APX depend on the architecture of its domains, substrate binding and orienting sites. Our results show that not all previously described catalytic amino acids are conserved. For instance, a substitution of W for L within the catalytic triad at position 325 can apparently be tolerated and it does not affect the enzymatic activity, since *G. max* APX (with L^325^) shows activity comparable to the *A. thaliana* orthologue (with W^325^) [50,51]. Uniquely, APXs of *L. major*, *L. seymouri*, and *N. esmeraldas* (and, probably, other representatives of Leishmaniinae [52]) possess the N-terminal anchoring trans-membrane domain that modulates their catalytic activity. While full-length proteins exhibit specific APX activities comparable to the cytosolic counterparts, deletion of this domain from *L. major* caused a 5-fold decrease in activity [34]. This highlights the importance of membrane tethering for enzyme architecture or substrate accessibility for this type of APX. On the other hand, the absence of several cation-binding residues has no effect on enzymatic activity, suggesting that they may be either redundant or not critical.

ROS production is a phenomenon, accompanying aerobic life, as both photosynthesis and respiration are major sources of ROS. However, we did not document a direct link between the rate of oxygen consumption and the activity of studied peroxidases. High ROS production via respiration is considered to recruit the CAT and APX systems to limit ROS reactivity [53]. However, we posit that this correlation may not be as straightforward. An increase in oxygen uptake was documented in *D. papillatum*, cultivated in the nutrient-poor medium, yet this had no effect on its CAT activity. Surprisingly, in thermostable *C. thermophila* and *L. seymouri*, elevated temperature triggered an increase in respiration, but a decrease in the activities of both CAT and APX. That transcript abundance did not follow the same pattern as activity can be explained by the key role of post-transcriptional regulation in the gene expression of euglenids and trypanosomatids [54,55,56,57,58]. Indeed, weak-to-moderate correlation has been observed between transcript abundance and protein levels in both trypanosomatids and *Euglena*. This suggests an important role of mRNA turnover, translational efficiency and protein degradation in modulating biological responses in these protists [57]. All euglenozoan protists are known for polycistronic transcription and the important role of post-transcriptional processes. Hence, the rather weak correlation between transcript levels and enzymatic activities of CAT and APX is not unexpected.

To conclude, despite the fact that the ROS detoxification systems in plants are upregulated under different conditions, we documented a similar activity pattern only for CAT in thermostable kinetoplastids, but not in the other studied euglenozoans. When present, these peroxidases appear to be constitutively transcribed, yet the extent of their enzymatic activity varies widely across the examined species. CAT and APX are retained (or horizontally acquired) only in a subset of studied protists, which is reflected in their complex phylogenies.

## 4. Materials and Methods 

### 4.1. Sequence Searches and Phylogenetic Analyses

CAT sequences from *D. papillatum* were found by tBLASTn [59] search in an unpublished genome and transcriptome assembly using kinetoplastid sequences, identified previously [29], as queries. The *D. papillatum* sequences served as queries for searches in the transcriptomes of other diplonemid species – *Diplonema japonicum*, *Rhynchopus humris*, *Lacrimia lanifica*, *Sulcionema specki*, *Artemidia motanka*, and *Namystynia karyoxenos*. 

APX sequences were downloaded from RedOxiBase [60] and GenBank [61] databases. These served as queries and references for APX identification in all studied euglenozoans and other protists. To distinguish between APX and cytochrome *c* peroxidases, with which they share the common phylogenetic origin [43], all euglenozoan sequences were submitted to the InterProScan [62]. Only those with identified APX domains or strong affiliation to the reference APX clades were retained for further phylogenetic analysis.

Sequences were clustered (50% identity and 80% coverage) using MMseqs2 [63]. Datasets were aligned by MAFFT [64] and poorly aligned positions were discarded by trimAl [65] using -gt 0.5 option. Maximum likelihood trees were inferred from the alignments using the LG + C20 + F + G model and the posterior mean site frequency method [66], LG + F + G guide tree in the IQ-TREE software [67] and employing the strategy of rapid bootstrapping followed by a “thorough” maximum likelihood search with 1000 bootstrap replicates.

### 4.2. Localization Predictions

To assess a putative subcellular localization of all studied euglenozoan proteins, PrediSi [68], NommPred (in kinetoplastid setting; [69]), TargetP v.2.0 [70], and MultiLoc2 (in fungal and animal settings; [71]) tools were employed. Glycosomal predictions were determined by an in-house python script based on previously identified targeting signals in kinetoplastids [28]. The number of transmembrane domains was predicted by TMHMM [72] or Phobius [73], implemented in the Geneious Prime software [74].

### 4.3. Transcript Expression Levels

Trimmed RNA-Seq reads were mapped onto the assembled transcriptomes using BBMap (part of the BBTools suite; https://jgi.doe.gov/data-and-tools/bbtools/). The expression values for each transcript were calculated as Fragments Per Kilobase of transcript per Million mapped reads (FPKM). From these, TPM (Transcripts Per Million) values were calculated as FPKM / sum(FPKMs) × 10^6^. For proteins with several transcript models, mean TPM was calculated.

### 4.4. Strains and Culture Conditions

*D. papillatum* cells were inoculated into the nutrient-rich and nutrient-poor media, to a final concentration 5 × 10^5^ cells/mL. The nutrient-rich medium contained 36 g/L sea salts (Red Sea), and 1% (v/v) horse serum (Sigma-Aldrich, St. Louise, USA), and 1 g/L tryptone (Duchefa Biochemie, Amsterdam, Netherlands), while the nutrient-poor medium consisted of 36 g/L sea salts, 1% (v/v) horse serum, and 0.01 g/L tryptone [75].

*R. humris* was cultivated in artificial sea water containing 3.6% sea salts (Sigma-Aldrich), enriched with 1% (v/v) heat-inactivated horse serum (Sigma-Aldrich), and 0.025 g/L LB broth powder (Amresco, Solon, USA) [76].

*Blastocrithidia* sp. P57 was cultivated in a medium composed of 40% (v/v) Schneider medium, 40% (v/v) RPMI medium, and 20% (v/v) inactivated fetal bovine serum (all Sigma-Aldrich).

*L. seymouri* and *C. thermophila* were cultivated at 23 °C in the Brain Heart Infusion medium (Sigma-Aldrich) supplemented with 10 μg/mL of hemin (Jena Bioscience, Jena, Germany), 10% fetal bovine serum, 100 units/mL of penicillin, and 100 μg/mL of streptomycin (all Sigma-Aldrich) [77]. For experiments at different temperatures (14 °C and 34 °C), cells were seeded at a concentration of 3 × 10^5^ cells/mL and cultured for 72 h as described previously [40]. 

*T. brucei* (strain 29-13) was cultured at 27 °C in SDM79 medium (GE Healthcare, Chicago, USA) containing 10% (v/v) heat-inactivated fetal bovine serum (Biosera, Nuaillé, France) and 2.5 mg/mL hemin [78].

*E. gracilis* cells were cultivated statically at 27 °C under constant illumination (10 μm/m^2^s^1^) and dark in liquid Hutner medium [79]. *E. longa* cells were cultivated statically under constant illumination (10 μm/m^2^s^1^) at 27 °C in Cramer-Myers medium supplemented with ethanol (0.8% v/v) [80].

### 4.5. RNA Isolation, Sequencing and Read Processing

Total RNA of 5 × 10^7^ cells from each *L. seymouri* and *C. thermophila* shifted to 14 °C was isolated using the RNeasy Mini kit (Qiagen, Hilden, Germany) according to the manufacturer’s instruction for three independent biological replicates. Paired-end strand-specific cDNA libraries were sequenced on Illumina NovaSeq platform (Macrogen Inc., Seoul, Korea). RNA-Seq reads were adapter and quality trimmed using BBDuk (part of the BBTools suite). As described above, trimmed reads were mapped onto previously assembled transcriptomes [40,41]. The raw sequencing data for *L. seymouri* and *C. thermophila* are available at NCBI (https://www.ncbi.nlm.nih.gov/) as BioProjects PRJNA611003 and PRJNA611063, respectively.

### 4.6. Protein Isolation

Five million cells of *D. papillatum* and *R. humris* were incubated with 2 mg of digitonin (AppliChem, Darmstadt, Germany) at room temperature for 5 min. Aliquot of 10^6^ lysed cells were used for activity measurements. 5 × 10^8^ *T. brucei*, *L. seymouri*, *C. thermophila, N. esmeraldas*, and *Blastocrithidia* sp. P57 cells were lysed on ice with 2% (w/v) dodecyl maltoside in 0.5 M aminocaproic acid (both AppliChem) for 1 h with subsequent 30 min centrifugation at 21,300× *g* at 4 °C. 10^5^ *E. gracilis* and *E. longa* cells were disrupted using 200 mg of 1 mm silica spheres (Silica matrix C; MP Biomedicals, Irvine, USA), and cycle of 3 × 15 s, 4.0 M/S on FastPrep-24 (MP Biomedicals) with cooling on ice between cycles. Residual intact cells were separated from lysate by centrifugation at 1800× *g* for 10 min at 4 °C. Supernatant was used for activity measurements. 

Protein concentration in cell lysates was determined using Bradford assay [81].

### 4.7. Measurements of Activities

Both CAT and APX activity were measured at 25 °C in whole-cell lysates. CAT activity was measured in total volume 1.3 mL that comprised 50 mM KPi pH 7.2, 0.005% (v/v) H_2_O_2_, and 10 µL of kinetoplastid lysate (*T. brucei*, *L. seymouri*, *C. thermophila, N. esmeraldas*, or *Blastocrithidia* sp. P57) or 100 µL of diplonemid lysate (*D. papillatum* or *R. humris*) or euglenid lysate (*E. gracilis* or *E. longa*). Activity of CAT was monitored for 2–4 min as a decrease in absorbance at 240 nm in the cuvette for UV-VIS spectra (Varian-Agilent Quartz Semi-Micro Cuvette Cell, Agilent, Santa Clara, USA). Activity U was calculated as the amount of enzyme that reduces 1 µmol of H_2_O_2_ (ε_240_ = 43.6 M^−1^cm^−1^) per 1 min. Specific activity was calculated as U per mg of cell proteins.

APX activity was measured analogously to CAT activity with the addition of freshly prepared 15 µM ascorbate (AppliChem). The activity was monitored for 2–4 min at 274 nm as a decrease in absorbance in the cuvette for UV-VIS spectra (Agilent). Activity U was calculated as the amount of enzyme required for a reduction of 1 µmol of ascorbic acid (ε_274_ = 14,900 M^−1^cm^−1^) per 1 min. Specific activity was calculated as U per mg of cell proteins.

In order to distinguish between H_2_O_2_ and ascorbate absorption spectra, we scanned absorbance of each substrate from 200 to 350 nm. Both molecules had absorption peaks at 300 nm, the wavelength at which CAT and APX activities are routinely detected [10,35]. After reducing the concentration of both substrates compared to the original protocols, 0.005% (v/v) H_2_O_2_ displayed an additional peak at 240 nm. Thus, we picked this wavelength (240 nm) for CAT measurements, because ascorbate did not absorb in this area. The control for CAT activity was the formation of oxygen that was visible in the cuvette in the form of bubbles. Since ascorbate displayed only one absorption peak around 300 nm, we adjusted the wavelength to 274 nm where 0.005% (v/v) H_2_O_2_ was not detected and only an increase in reduced ascorbate was monitored. Our optimized assay for APX activity measurement was closest to the protocol published previously [82], in which the concentration of ascorbate varied from 10 to 80 µM and oxidation of substrate was monitored at 265 nm with extinction coefficient for ascorbate ε_274_ = 14,900 M^−1^cm^−1^.

Each experiment was performed in two biological replicates. Each biological replicate consisted of 2–6 technical replicates. Statistical significance of differences between organisms was evaluated by unpaired *t*-test.

### 4.8. Oxygen Uptake Analysis

Clark oxygen electrode (Oxytherm System; Hansatech Instruments, Norfolk, UK) was used for measuring the oxygen consumption by intact cells. Each culture in the logarithmic growth phase was diluted to a concentration of 10^6^ cells/mL. The electrode chamber was filled with 1 mL of culture and oxygen consumption was recorded for 4–6 min. Final values were calculated as the difference in oxygen consumption per 1 min caused by 10^6^ cells. 

Each experiment was performed in two biological replicates. Each biological replicate consisted of two to six technical replicates. Statistical significance of differences between organisms was evaluated by unpaired *t*-test.

## Supplementary Materials

The following are available online at https://www.mdpi.com/2076-0817/9/4/317/s1, Figure S1: Full alignment of APX sequences from selected species. APX domains, signal and transit peptide, and linker are boxed by a colored background corresponding to domain colors in Figure 4. Important amino acid residues are highlighted in different colors corresponding to colors in Figure 4. Transmembrane domains are underlined. Table S1: Predicted subcellular localization of euglenozoan sequences used in this study. Table S2: Inferred expression levels and measurements of enzyme activities and oxygen uptake in studied euglenozoans. File S1: Maximum-likelihood phylogenetic tree of Euglenozoa HPX possessing APX domains in Newick format. File S2: Maximum-likelihood phylogenetic tree of Euglenozoa CAT in Newick format.
