# Supplementary material for: Catalase and Ascorbate Peroxidase in Euglenozoan Protists
Source: Pathogens. 2020 Apr 24;9(4):317. doi: 10.3390/pathogens9040317 (PMC7237987; doi:10.3390/pathogens9040317)
Supplement: Supplementary file 1 [file pathogens-09-00317-s001.pdf]

Catalase and ascorbate peroxidase in Euglenozoan protists

Ingrid Škodová-Sveráková[1,2,#,*], Kristína Záhonová[1,3,#], Barbora Bučková[2], Zoltán Füssy[3], Vyacheslav Yurchenko[1,4], Julius Lukeš[1,*]

[1] Institute of Parasitology, Biology Centre, Czech Academy of Sciences, České Budějovice (Budweis), Czech Republic

[2] Faculty of Natural Sciences, Comenius University, Bratislava, Slovakia

[3] Faculty of Science, Charles University, BIOCEV, Prague, Czech Republic

[4] Life Science Research Centre, Faculty of Science, University of Ostrava, Czech Republic

[#] Equal contribution

[*] Corresponding author: jula@paru.cas.cz; skodovaister@gmail.com

SUPPLEMENTARY FIGURE S1

SUPPLEMENTARY TABLES S1 – S2

SUPPLEMENTARY FILES S1 – S2

```
                        10        20        30        40        50        60        70        80
                ....|....|....|....|....|....|....|....|....|....|....|....|....|....|....|....|
A. thaliana     --------------------------MAERVSLTLNGTLLSPPPTTTTTTMSSSLRSTTAASLLLRSSSSSSRSTLTLSA
G. max          --------------------------------------------------------------------------------
L. major        ---------------------------------------------------------------------MFRCACVRLGQ
L. seymouri     ---------------------------------------------------------------------MFKCATVRLGQ
C. fasciculata  ---------------------------------------------------------------------MFKYASVRLGQ
N. esmeraldas   ---------------------------------------------------------------------MFRYAAVRLSQ
E. gracilis     MPAGLYDASLPMMAEHRRPAAAGQWVPLGAAALGALAALAAVALVWQPAPTETALFPRLGHRSRVAATPSTAPRAARAQH

                        90       100       110       120       130       140       150       160
                ....|....|....|....|....|....|....|....|....|....|....|....|....|....|....|....|
A. thaliana     SSSLSFVRSLVSSPRLSSS--SSLSQKKYRIASVNRSFNSTTAATKSSSSDPDQLKNAREDIKELLSTKF-CHPILVRLG
G. max          ---------------------------------------MGKSYPTVSADYQKAVEKAKKKLRGFIAEK-RCAPLMLRLA
L. major        RVLPSYVPRMSGTSRRAKGLFTGIAV--GTFVSGAMFVSCAS---ARVEEPPFDIRALRADIEDMISEKLELGPSLIRLA
L. seymouri     RLLPRALPRFSSGARRATGLFAGVAA--GTFFGGAAIVVCAP-GWSRVEEPPFDIRALRADIEDLVSNNLPLGPALVRLA
C. fasciculata  RLLPRMAPRFSTGVRRTAALCTGVAA--GAFVGGATFVFCAP-AKGAVEEPPFDIRGLRADIEDLISENLPLGPSIVRLA
N. esmeraldas   RVVPRLAPRLSRTPTRAKGLLAGIAV--GTVVSGVTFVSCAT-AKSRTREPPFDIAALRSDVEDLISDKLELGPALVRLA
E. gracilis     PPLPAARLPAAAAPRRDVALHAELPTWVPDFVKGLLEPPQPPYTLAEIRQVPWG--ELRRDLKALVQEK-QCAPILLRLA

                       170       180       190       200       210       220       230       240
                ....|....|....|....|....|....|....|....|....|....|....|....|....|....|....|....|
A. thaliana     WHDAGTYNKNIKEWPQRGGAN-GSLRFDIELKHAANAGLVNALNLIKDIKEKYSGISYADLFQLASATAIEEAGGPKIPM
G. max          WHSAGTYDVSSKT----GGP-FGTIKHPSELAHGANNGLDIAVRLLEPLKAEFPILTYADFYQLAGVVAVEVTGGPEVPF
L. major        WHEAASYDCFKKD----GSPNSASMRFKPECLYAGNKGLDIPRKALETLKKKYPQISYADLWVLAAYVAIEYMGGPTIPF
L. seymouri     WHEAGSFDCHKKD----GAANSASMRFKPECDYGGNKGLDVPRKALEPLKKKYPEISYADLWVLAAYVAIEYMGGPQIPF
C. fasciculata  WHEAGSYDCQRKD----GAPNSSSMRFKPECEYAGNRGLDVPRKALEPLKKKYPEISYADLWVLAAYVAIEYMGGPQIPF
N. esmeraldas   WHEAASYDSTKKD----GSPNSASMRFKPECMYAGNNGLDIPRAALEPLKKKYPQISYADLWVLASYVAIEYMGGPAIPY
E. gracilis     WHDAGTYDRASGT----GGPRAAMQYPGGEAAHGANAGLDIARNLLQPIREKYPTVSTADLWALASVVAIEVAGGPVIPF

                       250       260       270       280       290       300       310       320
                ....|....|....|....|....|....|....|....|....|....|....|....|....|....|....|....|
A. thaliana     KYGRVDASGPEDCPEEGRLPDAGPPSPATHLREVFYR-MGLDDKDIVALSGAHTLGRSRPERSGWGKPETKYTKEGPGAP
G. max          HPGREDKPEP---PPEGRLPDATKGS--DHLRDVFGKAMGLSDRDIVALSGGHTIGAAHKERSGFEGPWTSNPLIFDNSY
L. major        CWGRVDAKDGSVCGPDGRLPDGSKTQ--SHVREVFRR-LGFNDQETVALIGAHTCGECHIEFSGYHGPWTHDKNGFDNSF
L. seymouri     SWGRVDAKDGSVCGPDGRLPDAAQTQ--MHVRTVFTR-LGFNDQETVALIGAHTCGVCHLEYSGFVGPWTHDANGFDNSF
C. fasciculata  SWGRVDAKDGSVCGPDGRLPDGSQTQ--KHVRNVFTR-LGFNDQETVALIGAHTCGVCHLNYSGFVGPWTHDANGFDNSF
N. esmeraldas   SWGRVDAKDGAACGPDGRLPDGAQTQ--KHVRSVFTR-LGFNDQETVALIGAHTCGECHLKYSGFEGPWTHDKNGFDNSF
E. gracilis     RPGRRDAASAREAVEDGRLPDATRGP--DHLRAVFGR-MGLSDGEIVALSGAHTLGRAHVERSGFEGPWTEEPLKFDNTF

                       330       340       350       360       370       380       390       400
                ....|....|....|....|....|....|....|....|....|....|....|....|....|....|....|....|
A. thaliana     GGQSWTPEWLKFDNSYFKEIKEKRDEDLLVLPTDAAIFEDSSFKVYAEKYAADQDAFFKDYAVAHAKLSNLGAEFNPPEG
G. max          FKELLSGE----------------KEGLLQLPSDKALLSDPVFRPLVEKYASDEDAFFADYAEAHQKLSELGFAEA----
L. major        FTQLLDEDWVLNPKVEQMQLMDRATTKLMMLPSDVCLLLDPSYRKYVELYAKDNDRFNKDFANAFKKLTELGTRNLHKAP
L. seymouri     FTALLDEEWEVNPKVEKMQMMDRTTKRLMMLPSDIALILDPKYLKYVEMYAKDNERFNSDFAKAFKKLTELGTVNLHRAP
C. fasciculata  FTALLDEEWEVNPNVEQMQMMDRATKKLMMLPSDMALILDPKYKKYVEMYAKDNDRFNSDFSKAFKKLTELGTKNLHNAP
N. esmeraldas   FTELLDEDWVVNGKIQQMQMMDRATTRLMMLPSDMCLILDPKYRTYVELYAKDNDRFNSDFSKAFKKLTELGTSNLHPAP
E. gracilis     FTNLLNKKWTLGTSSAGKPQYTDETGTLMMLPSDMALLEDPIFRSYMEKYAKDEVAYFRDFATAYQRLAELGVPEL-PVP

                       410       420       430       440       450       460       470       480
                ....|....|....|....|....|....|....|....|....|....|....|....|....|....|....|....|
A. thaliana     IVI-----------------------------------------------------------------------------
G. max          --------------------------------------------------------------------------------
L. major        ASES----------------------------------------------------------------------------
L. seymouri     MPESA---------------------------------------------------------------------------
C. fasciculata  MPEEA---------------------------------------------------------------------------
N. esmeraldas   AAES----------------------------------------------------------------------------
E. gracilis     WDEIRADVAALVAEKGCAPILIRLAWHDAGTYDQQSNTGGPRAVMRFPGGEAEHGSNNGLDIARGLLQPIVDKYSWVSTA

                       490       500       510       520       530       540       550       560
                ....|....|....|....|....|....|....|....|....|....|....|....|....|....|....|....|
A. thaliana     --------------------------------------------------------------------------------
G. max          --------------------------------------------------------------------------------
L. major        --------------------------------------------------------------------------------
L. seymouri     --------------------------------------------------------------------------------
C. fasciculata  --------------------------------------------------------------------------------
N. esmeraldas   --------------------------------------------------------------------------------
E. gracilis     DLWAFASVVATEVSGGPKIPFRPGRRDAVTAKEAVERGRLPDATQTTNHLRDVFYRMGMTDEEIVALSGAHTMGRCHAER
```

```
                       570       580       590       600       610       620       630       640
                ....|....|....|....|....|....|....|....|....|....|....|....|....|....|....|....|
A. thaliana     --------------------------------------------------------------------------------
G. max          --------------------------------------------------------------------------------
L. major        --------------------------------------------------------------------------------
L. seymouri     --------------------------------------------------------------------------------
C. fasciculata  --------------------------------------------------------------------------------
N. esmeraldas   --------------------------------------------------------------------------------
E. gracilis     SGFEGPWTDNPLVFDNSYFKLLLERKWTAVTNSVGNLQFQDETGTLMMLTSDLALLMDPSFRKHVERFAADQDAFFRVYA

                       650       660
                ....|....|....|....|
A. thaliana     --------------------
G. max          --------------------
L. major        --------------------
L. seymouri     --------------------
C. fasciculata  --------------------
N. esmeraldas   --------------------
E. gracilis     GAYQKLTEGGCPFSKGAVPL
```

Figure S1: Full alignment of APX sequences from selected species. APX domains, signal and transit peptide, and linker are boxed by a colored background corresponding to domain colors in Fig. 4. Important amino acid residues are highlighted in different colors corresponding to colors in Fig. 4. Transmembrane domains are underlined.

Table S1. Predicted subcellular localization of euglenozoan sequences used in this study.

protein	organism	sequence	peroxisomal prediction	NommPred	TargetP	MultiLoc2 fungal	MultiLoc2 animal	PrediSI	No. TMDs	INFERRED
mitCCP	*Diplonema papillatum*	TR110952__c20_g26_i1__m.25215	-	_	mTP (36-37)	M: 0.62	S: 0.94	S (24)	0	M
eHPX	*Diplonema papillatum*	TR8739__c0_g1_i1__m.830	PTS1: SKL	M	mTP	S: 0.46	M: 0.41	-	0	M/G
eHPX	*Diplonema papillatum*	TR45711__c0_g1_i1__m.5672	-	M	SP (16-17)	S: 0.98	C: 0.46	S (16)	0	truncated N&C
eHPX	*Diplonema papillatum*	TR130880__c10_g1_i1__m.31721	-	_	SP (19-20)	S: 0.98	S: 0.9	S (17)	1	S
mitCCP	*Diplonema japonicum*	TRINITY_DN10300_c4_g1_i2	-	_	mTP (32-33)	M: 0.74	M: 0.87	-	0	M
mitCCP	*Diplonema japonicum*	TRINITY_DN10460_c6_g1_i1	PTS2: MSVSQNRLKVLNSHV	_	noTP	S: 0.68	C: 0.67	-	1	C
eHPX	*Diplonema japonicum*	TRINITY_DN10282_c5_g3_i2	-	M	mTP (23-24)	M: 0.72	M: 0.75	-	1	M
eHPX	*Diplonema japonicum*	TRINITY_DN8646_c0_g2_i1	-	_	SP (16-17)	S: 0.99	S: 0.89	S (16)	0	S
eHPX	*Diplonema japonicum*	TRINITY_DN10642_c2_g3_i1	-	_	SP (21-22)	S: 0.89	S: 0.84	S (21)	0	S; truncated C
eHPX	*Diplonema japonicum*	TRINITY_DN8053_c0_g1_i1	PTS1: SRI	_	noTP	S: 0.52	N: 0.43	-	1	truncated N
eHPX	*Rhynchopus humris*	TRINITY_DN40212_c0_g1_i1	-	_	mTP (34-35)	M: 0.65	M: 0.81	-	1	M
eHPX	*Rhynchopus humris*	TRINITY_DN23907_c1_g1_i1	-	_	SP (44-45)	S: 0.45	S: 0.4	S (44)	1	S; truncated C
eHPX	*Rhynchopus humris*	TRINITY_DN57024_c0_g1_i1	-	_	noTP	S: 0.89	S: 0.66	S (50)	1	S; truncated C
eHPX	*Rhynchopus humris*	TRINITY_DN63548_c0_g1_i1	-	_	noTP	S: 0.9	S: 0.83	-	0	truncated N&C
mitCCP	*Lacrimia lanifica*	TRINITY_DN7350_c0_g1_i1	-	_	mTP (26-27)	M: 0.71	M: 0.95	-	0	M
eHPX	*Lacrimia lanifica*	TRINITY_DN4895_c0_g1_i1	-	_	noTP	M: 0.56	M: 0.83	-	1	M
eHPX	*Lacrimia lanifica*	TRINITY_DN11643_c1_g8_i2	-	_	SP (19-20)	S: 0.99	S: 0.99	S (21)	1	S
eHPX	*Lacrimia lanifica*	TRINITY_DN11235_c0_g1_i6	-	_	SP (23-24)	S: 1.0	S: 0.99	S (23)	0	S; truncated C
eHPX	*Lacrimia lanifica*	TRINITY_DN11361_c1_g9_i2	-	_	SP (22-23)	S: 0.97	S: 0.72	S (22)	0	S; truncated C
eHPX	*Lacrimia lanifica*	TRINITY_DN8638_c0_g1_i1	-	_	noTP	S: 0.65	C: 0.57	-	1	truncated N
k-hAPX-CCP	*Lacrimia lanifica*	TRINITY_DN11414_c4_g13_i1	PTS2: MERLHVLKTHL	_	noTP	C: 0.64	C: 0.45	-	0	G
mitCCP	*Sulcionema specki*	TRINITY_DN80007_c0_g1_i1	-	_	mTP (36-37)	M: 0.59	M: 0.9	-	0	M
eHPX	*Sulcionema specki*	TRINITY_DN10012_c0_g1_i1	-	M	noTP	M: 0.39	M: 0.61	-	1	M
eHPX	*Sulcionema specki*	TRINITY_DN49103_c1_g2_i3	-	_	mTP (32-33)	M: 0.64	M: 0.89	-	0	M; truncated C
eHPX	*Sulcionema specki*	TRINITY_DN77967_c0_g1_i1	-	M	mTP (24-25)	M: 0.64	M: 0.95	-	0	M
eHPX	*Sulcionema specki*	TRINITY_DN50008_c0_g1_i1	-	_	SP (16-17)	S: 0.99	S: 0.81	S (16)	0	S
eHPX	*Sulcionema specki*	TRINITY_DN70017_c0_g1_i1	-	_	SP (17-18)	S: 0.97	S: 0.95	S (17)	0	S; truncated C
eHPX	*Sulcionema specki*	TRINITY_DN23548_c0_g1_i2	-	_	noTP	M: 0.58	C: 0.47	-	0	truncated N
pt/cytAPX-LGT	*Sulcionema specki*	TRINITY_DN24282_c1_g1_i1	PTS2: MEARRLDVLRSHI	_	noTP	C: 0.72	C: 0.44	-	0	G
mitCCP	*Artemidia motanka*	TRINITY_DN15980_c4_g3_i1	-	_	mTP (37-38)	M: 0.91	M: 0.92	S (22)	0	M
eHPX	*Artemidia motanka*	TRINITY_DN6375_c0_g1_i2	-	M	mTP (27-28)	M: 0.87	M: 0.92	-	0	M; truncated C
eHPX	*Artemidia motanka*	TRINITY_DN14823_c0_g2_i1	-	M	mTP (26-27)	M: 0.83	M: 0.82	-	1	M
eHPX	*Artemidia motanka*	TRINITY_DN24471_c0_g1_i1	-	M	mTP (24-25)	M: 0.73	M: 0.89	-	1	M
pt/cytAPX-LGT	*Artemidia motanka*	TRINITY_DN15980_c4_g5_i1	PTS2: MHRLAVVSSHL	_	noTP	C: 0.66	C: 0.49	-	0	G
pt-hAPX-CCP-LGT	*Artemidia motanka*	TRINITY_DN17334_c8_g3_i1	-	_	noTP	C: 0.63	C: 0.65	-	0	C
mitCCP	*Namystynia karyoxenos*	TRINITY_DN77678_c0_g1_i1	-	_	mTP (27-28)	M: 0.77	M: 0.98	-	0	M
eHPX	*Namystynia karyoxenos*	TRINITY_DN89651_c0_g1_i1	PTS2: MPPAVIAAQLLLPGRLRLLLRARRV	M	noTP	M: 0.58	M: 0.54	-	1	M/G
eHPX	*Namystynia karyoxenos*	TRINITY_DN15097_c0_g1_i1	-	_	noTP	M: 0.76	M: 0.92	S (44)	1	M
eHPX	*Namystynia karyoxenos*	TRINITY_DN52435_c0_g1_i1	-	M	mTP (33-34)	M: 0.71	M: 0.86	-	1	M
eHPX	*Namystynia karyoxenos*	TRINITY_DN26871_c0_g1_i1	-	_	SP (28-29)	S: 0.99	M: 0.86	S (28)	0	S
pt/cytAPX-LGT	*Namystynia karyoxenos*	TRINITY_DN89136_c0_g1_i1	PTS2: MAARRIDVLSSHV	_	noTP	M: 0.37	C: 0.47	-	0	G

pt-hAPX-CCP-LGT	*Namystynia karyoxenos*	TRINITY_DN22390_c0_g1_i1	-	_	noTP	C: 0.7	N: 0.64	-	0	C
pt-hAPX-CCP	*Euglena gracilis*	GEFR01005776.1/BAC05484	-	_	noTP	S: 0.37	M: 0.81	S (43)	1	P
g-ptAPX	*Euglena gracilis*	GDJR01039455.1	-	_	noTP	S: 0.45	M: 0.63	S (49)	2	truncated N
eHPX	*Euglena gracilis*	GDJR01082147.1	-	_	noTP	S: 0.92	C: 0.6	-	0	truncated N&C
eHPX	*Euglena gracilis*	GDJR01082152.1	-	_	noTP	S: 0.9	C: 0.74	-	0	truncated N&C
eHPX	*Euglena gracilis*	GEFR01009177.1 (C-terminal of GDJR01082147.1)	-	_	noTP	S: 0.83	C: 0.67	-	0	truncated N&C
eHPX	*Euglena gracilis*	GEFR01013723.1	-	_	noTP	S: 0.97	C: 0.71	-	0	truncated N&C
eHPX	*Euglena gracilis*	GEFR01042768.1	-	_	noTP	S: 0.94	S: 0.7	-	0	truncated N&C
eHPX	*Euglena longa*	GGOE01045416.1 (Contig45548)	-	_	SP (19-20)	S: 0.99	S: 0.95	S (19)	0	S; truncated C
pt-hAPX-CCP	*Eutreptiella gymnastica* NIES-381	CAMNT_0000682101	-	_	noTP	S: 0.54	C: 0.74	-	0	truncated N
pt-hAPX-CCP	*Eutreptiella gymnastica* NIES-381	CAMNT_0000688179	PTS1: NKA	_	noTP	S: 0.75	M: 0.37	S (44)	1	P
pt-hAPX-CCP	*Eutreptiella gymnastica* NIES-381	CAMNT_0000690323	-	_	noTP	S: 0.47	C: 0.52	S (42)	1	P
pt-hAPX-CCP	*Eutreptiella gymnastica* NIES-381	CAMNT_0000693181	-	_	noTP	C: 0.51	C: 0.45	S (40)	1	P
pt-hAPX-CCP	*Eutreptiella gymnastica* NIES-381	CAMNT_0000708097	-	_	noTP	S: 0.47	C: 0.52	S (42)	1	P
g-ptAPX	*Eutreptiella gymnastica* NIES-381	Transcript_62771	-	_	noTP	C: 0.44	C: 0.39	S (50)	0	truncated N
g-ptAPX	*Eutreptiella gymnastica* NIES-381	Transcript_108943	-	_	SP (28-29)	M: 0.57	S: 0.95	S (31)	3	P; truncated C
g-ptAPX	*Eutreptiella gymnastica* NIES-381	MMETSP0039_2-20120614_21586	-	_	noTP	M: 0.59	M: 062	S (35)	3	P
pt-hAPX-CCP	*Eutreptiella gymnastica* -like CCMP1594	CAMNT_0046500393	-	_	noTP	S: 0.59	M: 0.68	S (48)	1	P
pt-hAPX-CCP	*Eutreptiella gymnastica* -like CCMP1594	CAMNT_0046515501	-	_	noTP	S: 0.52	M: 0.63	S (41)	1	P
g-ptAPX	*Eutreptiella gymnastica* -like CCMP1594	MMETSP0809_Transcript_4877	-	_	noTP	M: 0.47	S: 0.59	S (40)	2	P
g-ptAPX	*Eutreptiella gymnastica* -like CCMP1594	MMETSP0811_Transcript_101747	-	_	noTP	S: 0.57	S: 0.55	S (33)	2	P
hAPX-CCP	*Blechomonas ayalai*	Baya_117_0180	-	M	mTP (26-27)	M: 0.53	M: 0.86	-	1	M
hAPX-CCP	*Crithidia fasciculata*	CFAC1_290028200.1	-	M	mTP (49-50)	C: 0.44	M: 0.95	S (43)	0	M
hAPX-CCP	*Crithidia thermophila*	c6407_g1_i1	-	M	mTP (28-29)	C: 0.48	M: 0.92	-	1	M
hAPX-CCP	*Endotrypanum monterogeii*	EMOLV88_340006000.1	-	M	mTP (49-50)	M: 0.36	M: 0.81	S (52)	0	M
hAPX-CCP	*Leishmania enriettii*	LENLEM3045_340005600.1	-	M	mTP (52-53)	M: 0.39	M: 0.88	S (52)	1	M
hAPX-CCP	*Leishmania major Friedlin*	LmjF.34.0070	-	M	mTP (46-47)	C: 0.41	M: 0.87	S (52)	1	M
hAPX-CCP	*Leishmania mexicana*	LmxM.33.0070.1	-	_	SP (32-33)	M: 0.45	M: 0.73	S (32)	1	M
hAPX-CCP	*Leishmania* sp. MAR LEM2494	LMARLEM2494_340005600.1	-	M	SP (35-36)	M: 0.44	C: 0.4	S (35)	1	M
hAPX-CCP	*Leptomonas pyrrhocoris*	LpyrH10_05_0250	-	M	mTP (21-22)	M: 0.5	M: 0.9	S (43)	1	M
hAPX-CCP	*Leptomonas seymouri*	Lsey_0078_0210	-	M	mTP (27-28)	C: 0.41	M: 0.96	S (45)	1	M
hAPX-CCP	*Novymonas esmeraldas*	NODE_82	PTS2: MFRYAAVRLSQRVVPRLAPRLSRTPTRA	_	mTP (52-53)	M: 0.51	M: 0.88	S (52)	1	M
hAPX-CCP	*Paratrypanosoma confusum*	PCON_0074310.mRNA	-	_	noTP	C: 0.6	C: 0.54	-	0	truncated N
hAPX-CCP	*Strigomonas culicis*	EPY37063.1	-	M	mTP (28-29)	M: 0.48	M: 0.88	-	0	M
hAPX-CCP	*Trypanosoma cruzi* Dm28c 2018	C4B63_47g23-t42_1	-	M	noTP	C: 0.56	M: 0.61	S (50)	0	M
hAPX-CCP	*Trypanosoma cruzi marinkellei*	Tc_MARK_3376	PTS2: MFFRLGSFFSKY	M	mTP (12-13)	C: 0.56	M: 0.67	-	1	M
hAPX-CCP	*Trypanosoma grayi*	DQ04_02591010-t26_1	-	M	mTP (51-52)	M: 0.49	M: 0.8	S (47)	0	M
hAPX-CCP	*Trypanosoma theileri*	TM35_000011050-t36_1	-	M	mTP (34-35)	C: 0.56	M: 0.88	S (45)	1	M
CAT	*Diplonema papillatum*	DIPPA_00897.mRNA.1	-	_	noTP	C: 0.88	C: 0.77	-	0	C
CAT	*Diplonema papillatum*	DIPPA_00900.mRNA.1	-	_	noTP	C: 0.88	C: 0.77	-	0	C
CAT	*Diplonema papillatum*	DIPPA_00901.mRNA.1	-	_	noTP	C: 0.88	C: 0.77	-	0	C
CAT	*Diplonema papillatum*	DIPPA_00904.mRNA.1	-	_	noTP	C: 0.8	C: 0.67	-	0	C
CAT	*Diplonema japonicum*	TRINITY_DN11173_c4_g2_i1	PTS1: SQA	_	SP (20-21)	S: 0.93	S: 0.94	S (16)	0	S
CAT	*Diplonema japonicum*	TRINITY_DN11173_c4_g3_i1	PTS1: SQA	_	SP (16-17)	S: 0.89	S: 0.92	S (16)	0	S
CAT	*Diplonema japonicum*	TRINITY_DN11173_c4_g3_i2	PTS1: SQA	_	SP (16-17)	S: 0.91	S: 0.92	S (16)	0	S

CAT	*Rhynchopus humris*	TRINITY_DN23544_c0_g1_i1	-	_	SP (19-20)	S: 0.91	S: 0.9	S (19)	0	S
CAT	*Sulcionema specki*	TRINITY_DN284_c0_g1_i1	-	_	SP (17-18)	S: 0.92	S: 0.94	S (17)	0	S
CAT	*Artemidia motanka*	TRINITY_DN17294_c8_g2_i1	-	_	SP (18-19)	S: 0.94	S: 0.92	S (18)	0	S
CAT	*Artemidia motanka*	TRINITY_DN17294_c8_g5_i1	-	_	SP (23-24)	S: 0.93	S: 0.95	S (16)	0	S
CAT	*Artemidia motanka*	TRINITY_DN17294_c8_g7_i1	-	_	SP (16-17)	S: 0.89	S: 0.86	S (16)	0	S
CAT	*Namystynia karyoxenos*	TRINITY_DN46753_c0_g1_i1	-	_	SP (19-20)	S: 0.91	S: 0.84	S (19)	0	S; truncated C
CAT	*Namystynia karyoxenos*	TRINITY_DN46753_c0_g1_i2	-	_	SP (19-20)	S: 0.9	S: 0.87	S (19)	0	S
CAT	*Namystynia karyoxenos*	TRINITY_DN46753_c0_g1_i3	-	_	SP (19-20)	S: 0.91	S: 0.9	S (19)	0	S
CAT	*Namystynia karyoxenos*	TRINITY_DN46753_c0_g1_i4	-	_	SP (19-20)	S: 0.91	S: 0.86	S (19)	0	S; truncated C
CAT	*Namystynia karyoxenos*	TRINITY_DN57307_c0_g1_i1	-	M	SP (17-18)	S: 0.96	C: 0.49	S (17)	0	S
CAT	*Novymonas esmeraldas*	ANJ89233	-	_	noTP	C: 0.91	C: 0.8	-	0	C
CAT	*Blastocrithidia* sp. P57	TRINITY_GG_3943_c1_g1_i1	-	_	noTP	C: 0.9	C: 0.79	-	0	C
CAT	*Blastocrithidia triatomae*	NODE_379_length_14747_cov_61.7013	-	_	noTP	C: 0.89	C: 0.79	-	0	C
CAT	*jaculum*	NODE_189_length_21547_cov_70.3833	-	_	SP (19-20)	M: 0.4	S: 0.47	S (19)	2	S
CAT	*Crithidia acanthocephali*	caca	-	_	noTP	C: 0.74	C: 0.67	-	0	C
CAT	*Crithidia bombi*	cbom	-	_	noTP	C: 0.91	C: 0.79	-	0	C
CAT	*Crithidia brevicula*	ANJ89235	-	_	noTP	C: 0.92	C: 0.82	-	0	truncated N&C
CAT	*Crithidia fasciculata*	CFAC1_160031400	-	_	noTP	C: 0.9	C: 0.79	-	0	C
CAT	*Crithidia mellificae*	cmel	-	_	noTP	C: 0.79	C: 0.78	-	0	C
CAT	*Crithidia thermophila*	ANJ89234	-	_	noTP	C: 0.87	C: 0.79	-	0	C
CAT	*Leptomonas pyrrhocoris*	XP_015656182	-	_	noTP	C: 0.9	C: 0.79	-	0	C
CAT	*Leptomonas seymouri*	KPI89353	-	_	noTP	C: 0.9	C: 0.79	-	0	C
CAT	*Lotmaria passim*	lpas	-	_	noTP	C: 0.9	C: 0.81	-	0	C

mitCCP, mitochondrial CCP

eHPX, euglenozoa specific heme peroxidase

k-hAPX-CCP, kinetoplastid specific hybrid APX-CCP

pt-hAPX-CCP, plastid hybrid APX-CCP

pt-hAPX-CCP-LGT, plastid hybrid APX-CCP, gained by LGT

pt/cytAPX-LGT, plastid/cytosolic APX, gained by LGT

g-ptAPX, green lineage specific plastid APX

CAT, catalase

M, mitochondrion

P, plastid

G, glycosome

S, secretory pathway

C, cytosol

N, nucleus

truncated N, truncated sequence at N-terminus

truncated C, truncated sequence at C-terminus

truncated N&C, truncated sequence at both termini

Table S2. Inferred expression levels and measurements of enzyme activities and oxygen uptake in studied euglenozoans.

			CATALALASE					ASCORBATE PEROXIDASE					RESPIRATION
lineage	species	condition	contig	FPKM	TPM	TPM mean	activity [U/mg]	contig	FPKM	TPM	TPM mean	activity [mU/mg]	[O2 µmol/ min*10^6 cells]
Diplonemids	*Diplonema papillatum*	rich medium	DIPPA_00897.mRNA.1	80.87	112.16	216.21	3.99 ± 1.48	n.a.	n.a.	n.a.	n.a.	0	2.42 ± 0.98
			DIPPA_00900.mRNA.1	459.22	636.89								
			DIPPA_00901.mRNA.1	56.43	78.26								
			DIPPA_00904.mRNA.1	27.06	37.52								
		poor medium	DIPPA_00897.mRNA.1	92.55	131.25	256.86	6.27 ± 3.47	n.a.	n.a.	n.a.	n.a.	0	7.83 ± 2.81
			DIPPA_00900.mRNA.1	505.04	716.26								
			DIPPA_00901.mRNA.1	107.16	151.97								
			DIPPA_00904.mRNA.1	19.72	27.97								
	Rhynchopus humris		TRINITY_DN23544_c0_g1_i1	30.45	25.39	25.39	0	n.a.	n.a.	n.a.	n.a.	3.69 ± 1.80	15.42 ± 6.09
Kinetoplastids	*Blastocrithidia* sp. P57		TRINITY_GG_3943_c1_g1_i1	21.04	26.93	26.93	0	n.a.	n.a.	n.a.	n.a.	2.06 ± 1.84	2.44 ± 0.67
	Leptomonas seymouri	14 °C	Lsey_0026_0490	1117.21	813.49	813.49	16.84 ± 1.94	Lsey_0078_0210	225.21	163.98	163.98	54.26 ± 21.32	5.84 ± 0.90
		23 °C	Lsey_0026_0490	106.45	116.80	116.80	12.90 ± 6.48	Lsey_0078_0210	15.23	16.71	16.71	39.79 ± 10.96	8.93 ± 0.52
		34 °C	Lsey_0026_0490	398.47	432.93	432.93	5.07 ± 3.81	Lsey_0078_0210	38.24	41.55	41.55	34.35 ± 7.93	7.86 ± 0.61
	Crithidia thermophila	14 °C	TR9337|c1_g1_i1/ANJ89234	384.23	567.61	567.61	17.19 ± 6.06	TR7333|c1_g1_i1/c6407_g1_i1	122.50	180.96	180.96	65.40 ± 28.87	6.21 ± 0.16
		23 °C	TR9337|c1_g1_i1/ANJ89234	457.21	678.91	678.91	13.19 ± 2.43	TR7333|c1_g1_i1/c6407_g1_i1	12.14	18.03	18.03	48.63 ± 19.51	10.92 ± 3.15
		34 °C	TR9337|c1_g1_i1/ANJ89234	646.46	972.86	972.86	4.79 ± 1.11	TR7333|c1_g1_i1/c6407_g1_i1	18.42	27.72	27.72	51.57 ± 27.44	11.82 ± 1.16
	Novymonas esmeraldas		TRINITY_GG_6749_c0_g1_i1/ANJ89233	927.28	1220.49	626.23	10.76 ± 2.43	NODE_82	56.08	73.81	73.81	206.63 ± 69.37	4.78 ± 0.20
			TRINITY_GG_6749_c0_g1_i2/ANJ89233	24.30	31.97								
	Trypanosoma brucei		n.a.	n.a.	n.a.	n.a.	0	n.a.	n.a.	n.a.	n.a.	0	2.95 ± 0.67
Euglenids	*Euglena gracilis*	light	n.a.	n.a.	n.a.	n.a.	0	GEFR01005776.1/BAC05484	12.51	13.79	12.00	625.22 ± 176.36	2.37 ± 1.22
								GEFR01016514.1/GDJR01039455.1	9.18	10.12			
		dark	n.a.	n.a.	n.a.	n.a.	0	GEFR01005776.1/BAC05484	9.27	9.87	8.30	0	15.81 ± 4.42
								GEFR01016514.1/GDJR01039455.1	6.55	6.79			
	Euglena longa		n.a.	n.a.	n.a.	n.a.	0	n.a.	n.a.	n.a.	n.a.	0	23.65 ± 3.69

n.a.; not available

File S1: Maximum-likelihood phylogenetic tree of Euglenozoa HPX possessing APX domains in Newick format.

(Acanthoeca_MMETSP0105_Gene.63460-
Transcript_35775_Opisthokonta_Choanoflagellida:0.4799428336,(((((((((((((((Alexandrium-temarense-CCMP1771-
20130823_405166_1:0.2582221535,Scrippsiella-hangoei-SHTV5-
20131105_129680_1:0.4488957369):0.4045670100,Crypthecodinium_cohnii_WH_Provasly-
Seligo_CAMPEP_0193932514:0.3012689995):0.1206385833,(((((Aplanochytrium-sp-PBS07-
20140214_161485_1:0.3555901191,(SEED_Artemidia_motanka_TRINITY_DN15980_c4_g5_i1:0.2676357074,(SEED_Na
mystynia_karyoxenos_TRINITY_DN89136_c0_g1_i1:0.1100771516,SEED_Sulcionema_specki_TRINITY_DN24282_c1_g1_i
1:0.2552773747):0.0816210166):0.1010433252):0.0555519179,Undescribed-sp-CCMP2436-
20130826_9488_1:0.4974049891):0.0089568098,(SEED_Monosiga_brevicollis__Choanoflagellata__APX2_6306_MbrAPx
01:0.3249027813,SEED_Salpingoeca_sp.__Choanoflagellata__APX2_11129_SAspAPx01:0.6406658181):0.1564732249):0
.0532104613,Chrysochromulina-polylepis-CCMP1757-
20130903_147481_1:0.3577300785):0.0740498441,((((Durinskia_baltica_CSIRO_CS-
38_CAMPEP_0199942210:0.1786917746,Symbiodinium_microadriaticum_OLP81201.1_putative_L-
ascorbate_peroxidase_7__chloroplastic:0.3760242337):0.0809307226,Karenia_brevis_MMETSP0201_Gene.62695-
Transcript_54590_Dinophyta_Gymnodiniales:0.3607998209):0.0844633386,(((SEED_Heterocapsa_triquetra__Dinoflagel
lata__APX2_2364_HtrAPx01:0.2657416377,SEED_Karlodinium_micrum__Dinoflagellata__APX2_3944_KmAPx01:0.1584
784386):0.0857746088,Symbiodinium_microadriaticum_OLP89888.1_putative_L-
ascorbate_peroxidase_7__chloroplastic:0.1643165697):0.0374100190,SEED_Oxyrrhis_marina__Dinoflagellata__APX2_1
3535_OmarAPx01:0.2671773075):0.1133740826):0.0327784263,(((Ostreococcus_tauri_XP_003074417.1_chloroplast_as
corbate_peroxidase__ISS_:0.2860756314,Pyramimonas-parkeae-CCMP726-
20131001_721_1:0.3673569433):0.0775410388,SEED_Micromonas_pusilla__Chlorophyta__APX2_11130_MpuAPx01:0.
2389665078):0.0841747686,SEED_Bigelowiella_natans__Rhizaria__APX2_2512_BnaAPx01:0.4265877014):0.086846008
4):0.0663447642):0.0682003812):0.0667212430,(((((((Dinophysis_acuminata_CAMPEP_0179227314_Dinophyta_Dinoph
ysiales:0.5107858685,(SEED_Phaeodactylum_tricornutum__Stramenopila__APX2_2571_PtrAPx01:0.0608757369,SEED_
Thalassiosira_pseudonana__Stramenopila__APX2_2558_TpsAPx02:0.1482984233):0.0608750835):0.0448343751,SEED_
Guillardia_theta__Cryptophyta__APX2_13019_GtAPx02:0.0687858912):0.0173975934,Rhodomonas_lens_MMETSP048
4_Gene.19640-
Transcript_12385_Cryptophyta_Pyrenomonadales:0.1983868215):0.0541687749,Guillardia_theta_MMETSP0046_Gene.
34396-Transcript_14618_Cryptophyta_Pyrenomonadales:0.1685588972):0.1751092276,(Hemiselmis-
tepida_MMETSP1355-
20121228_35325_1:0.2917722247,Klebsormidium_nitens_GAQ79980.1_ascorbate_peroxidase:0.6165955307):0.05745
67637):0.0389664407,(Dunaliella-tertiolecta-CCMP1320-
20130909_10980_1:0.3430469622,(Klebsormidium_nitens_GAQ80163.1_ascorbate_peroxidase:0.2782663029,((((SEED
_Arabidopsis_thaliana__Streptophyta__APX2_1886_AtAPx05:0.1322120075,Sorghum_bicolor_XP_021316116.1_probab
le_L-
ascorbate_peroxidase_8__chloroplastic_isoform_X1:0.1119127387):0.0653577370,SEED_Physcomitrella_patens__Strep
tophyta__APX2_2497_PpaAPx02:0.2885329011):0.0702716452,SEED_Selaginella_moellendorffii__Streptophyta__APX2
_7198_SmAPx03:0.1487839545):0.1455879782,(SEED_Chlamydomonas_reinhardtii__Chlorophyta__APX2_1958_CreAPx
02:0.2829783581,SEED_Chlorella_vulgaris__Chlorophyta__APX2_2636_CvAPx01:0.3989233367):0.0899889104):0.0887
840974):0.0674395935):0.0321346243):0.0992350738,((SEED_Ectocarpus_siliculosus__Stramenopila__APX2_13374_Esi
lAPx01:0.4058125973,SEED_Vitrella_brassicaformis_CEM12648.1:0.3847436229):0.0425200356,SEED_Vitrella_brassicaf
ormis__Alveolata__APX2_13536_VbraAPx01:0.3683347766):0.0187912019):0.1109479328):0.1143727114,(((Chlamydo
monas-euryale_MMETSP0063_2-20130828_62321_1:0.1033356054,Tetraselmis-striata-LANL1001-
20140214_117058_1:0.1448357222):0.2985933071,SEED_Cyanidioschyzon_merolae__Rhodophyta__APX2_2611_CmeA
Px01:0.4471611357):0.1024478914,(Climacostomum-virens_MMETSP1397-
20131031_1011_1:0.5940688572,SEED_Perkinsus_marinus__Alveolata__APX2_11145_PmariAPx01:0.4953608562):0.19
81606060):0.0730142215):0.0432083832,(Proteomonas-sulcata_MMETSP1049-
20130417_3516_1:0.6546435603,SEED_Guillardia_theta__Cryptophyta__APX2_13353_GtAPx03:0.4818331555):0.3717

679710):0.0604571353,(((((((((Alexandrium_tamarense_MMETSP0384_Gene.140806-
Transcript_86135_Dinophyta_Gonyaulacales:0.7830904689,Prasinococcus_capsulatus_MMETSP0941_Gene.17794-
Transcript_6790_Chlorophyta_Prasinococcales:1.8730203545):0.5352779285,(Micromonas_MMETSP1080-
20121227_4761_1:0.2158839597,SEED_Micromonas_pusilla__Chlorophyta__APX4_15865_MpuAPx02:0.1552600820):1
.1898182960):0.4226018126,(((Euglena_gracilis_GDJR01039455.1_m.86725_Euglenozoa_Euglenophyta:0.7147021263,E
utreptiella_gymnastica-like_CCMP1594_MMETSP0811_Gene.96963-
Transcript_101747_Euglenozoa_Euglenophyta:0.3988134961):0.0842608711,((Eutreptiella-
gymnastica_MMETSP0039_2-20120614_21586_1:0.8743744586,(Eutreptiella_gymnastica_NIES-
381_Transcript_108943-m.115750_Euglenozoa_Euglenophyta:0.5635037737,Eutreptiella_gymnastica_NIES-
381_Transcript_62771-
m.69171_Euglenozoa_Euglenophyta:0.5751075809):0.1316417824):0.3135583150,Eutreptiella_gymnastica-
like_CCMP1594_MMETSP0809_Transcript_4877-
m.4987_Euglenozoa_Euglenophyta:0.5320652761):0.0609262896):0.4316228405,Pyramimonas-
obovata_MMETSP1169-
20130426_1770_1:1.2273192502):0.4599544969):0.0523293246,(((Chlorella_variabilis_XP_005847371.1_hypothetical_
protein_CHLNCDRAFT_48220:0.6830188395,((Picochlorum_sp._MMETSP1330_Transcript_3147-
m.6609_Chlorophyta_Trebouxiophyceae:0.7589710490,Tetraselmis-striata-LANL1001-
20140214_117711_1:0.9834187614):0.1199234140,(SEED_Chlamydomonas_reinhardtii__Chlorophyta__APX4_2805_Cr
eAPx01:0.2963862399,SEED_Volvox_carteri__Chlorophyta__APX4_8560_VcaAPx01:0.1644153866):0.2582753526):0.11
31649233):0.1373537343,Stichococcus_MMETSP1473-
20131121_25656_1:0.7323608821):0.1519108230,(Nephroselmis-pyriformis_MMETSP0034_2-
20130328_17760_1:1.0912089801,Picocystis-salinarum-CCMP1897-
20130926_1711_1:0.9337169191):0.3610567570):0.4482953105):0.0836153780,(Pycnococcus_MMETSP1085-
20130531_6256_1:0.5536861591,uncultured-eukaryote_MMETSP1310-
20130426_10555_1:0.9830490600):1.1825651590):0.1655627441,Klebsormidium_nitens_GAQ82344.1_ascorbate_pero
xidase:1.0122206394):0.0814803130,(((SEED_Arabidopsis_thaliana__Streptophyta__APX4_3920_AtAPx07:0.235022873
0,xxx_Corchorus_capsularis_OMO94487.1_heme_peroxidase__plant_fungal_bacterial:0.1204201789):0.0797946518,(S
EED_Brachypodium_distachyon__Streptophyta__APX4_7925_BdiAPx08:0.1360015548,xxx_Dichanthelium_oligosanthes
_OEL32843.1_Thylakoid_lumenal_29_kDa_protein__chloroplastic:0.0805090817):0.0728186905):0.2083346798,SEED_
Marchantia_polymorpha__Streptophyta__APX4_7344_MpAPx04:0.4335800602):0.2410027466):0.7729424948,((((Arabi
dopsis_thaliana_Q9LNL0_Viridiplantae_Streptophyta:1.4454062281,Oryza_sativa_Japonica_NP_001048638_Viridiplanta
e_Streptophyta:0.3939245907):0.4831084507,Chattonella-subsalsa-CCMP2191-
20130903_28645_1:1.2110500361):0.1701475286,SEED_Chlorella_sorokiniana__Chlorophyta__APX7_15185_CsorAPx0
2:1.8990925135):0.2967643363,(Chlamydomonas_reinhardtii_MMETSP1391_Gene.16250-
Transcript_9787_Chlorophyta_Chlorophyceae:0.5506001715,Klebsormidium_nitens_GAQ79820.1_L-
ascorbate_peroxidase:0.7974480598):0.2849054977):0.1016400712):0.0326584993,(((Eutreptiella_gymnastica_NIES-
381_Transcript_79356-m.86242_Euglenozoa_Euglenophyta:0.3931838200,(Ostreococcus-_lucimarinus__MMETSP0939-
20121108_1360_1:0.6956540524,Pyramimonas-parkeae-CCMP726-
20131001_13231_1:0.4894516038):0.0666004937):0.1426730017,(Stichococcus_MMETSP1473-
20131121_7338_1:0.6029321284,Tetraselmis-striata-LANL1001-
20140214_3195_1:0.4505865374):0.0989852857):0.2186612717,((Fragaria_vesca_XP_011459177.1_putative_L-
ascorbate_peroxidase_6:0.4016330766,Prasinococcus_capsulatus_MMETSP0941_Gene.14333-
Transcript_5569_Chlorophyta_Prasinococcales:0.7476716751):0.1223779590,Prasinoderma-coloniale_MMETSP0806_2-
20130528_8641_1:1.1681884745):0.0413322735):0.3570991027):0.1236423106,(Cyanoptyche-
gloeocystis_MMETSP1086-20130531_37175_1:1.0671494049,Synchroma-pusillum_MMETSP1452-
20131203_3767_1:1.9245949090):0.2945069749):0.4026632068):0.0990374925,(Pyramimonas-parkeae-CCMP726-
20131001_134760_1:1.1616828668,(((SEED_Arabidopsis_thaliana__Streptophyta__APX1_1890_AtAPx01:0.1765574573
,(SEED_Brachypodium_distachyon__Streptophyta__APX1_7906_BdiAPx01:0.1008721002,SEED_Selaginella_moellendorf
fii__Streptophyta__APX1_3324_SmAPx01:0.2512911639):0.0940626885):0.0066904395,SEED_Physcomitrella_patens__

Streptophyta__APX1_2325_PpaAPx01:0.3135808018):0.1753091331,(((SEED_Arabidopsis_thaliana__Streptophyta__AP
X3_1891_AtAPx03:0.1015188312,SEED_Brachypodium_distachyon__Streptophyta__APX3_7876_BdiAPx03:0.14388665
75):0.0556699894,(SEED_Physcomitrella_patens__Streptophyta__APX3_2498_PpaAPx03:0.1519719551,SEED_Selaginell
a_moellendorffii___Streptophyta__APX3_7196_SmAPx02:0.0932756415):0.0701120110):0.0275691237,SEED_Arabidop
sis_thaliana__Streptophyta__APX5_1887_AtAPx04:0.3280895109):0.0821916580):0.1051538951):0.0376072613):0.049
8332631,(((Erythrolobus-australicus_MMETSP1353-
20130828_18498_1:0.3527052062,SEED_Porphyra_yezoensis__Rhodophyta__APX6_13396_PyAPx01:0.2264833863):0.
0550265548,(SEED_Chondrus_crispus__Rhodophyta__APX6_2631_CcriAPx01:0.4241618486,(SEED_Chondrus_crispus__
Rhodophyta__APX6_2632_CcriAPx02:0.2953298351,SEED_Galdieria_sulphuraria__Rhodophyta__APX6_2566_GsAPx01:
0.3723963802):0.0258335203):0.0481023470):0.0649685213,SEED_Porphyridium_cruentum__Rhodophyta__APX6_133
97_PcruAPx01:0.4178863315):0.1519615314):0.1939124340,((((SEED_Euglena_gracilis__APX-
CCP_BAC05484:0.1618286676,SEED_Euglena_sp.__Euglenida__APX-CCP_13415_EUgAPx-
CcP03:0.1150075262):0.1359099047,(SEED_Eutreptiella_gymnastica-
like_CCMP1594_CAMNT_0046500393:0.1139509626,SEED_Eutreptiella_gymnastica-
like_CCMP1594_CAMNT_0046515501:0.0704616901):0.1045292970):0.0646606604,SEED_Eutreptiella_gymnastica_NIE
S-381_CAMNT_0000688179:0.1727320646):0.1031606172,(SEED_Eutreptiella_gymnastica_NIES-
381_CAMNT_0000682101:0.1997848076,((SEED_Eutreptiella_gymnastica_NIES-
381_CAMNT_0000690323:0.0000023451,SEED_Eutreptiella_gymnastica_NIES-
381_CAMNT_0000708097:0.0000023451):0.0000020636,SEED_Eutreptiella_gymnastica_NIES-
381_CAMNT_0000693181:0.0523283508):0.0142065732):0.1791774229):0.1703681345):0.1229264546,((((Chrysochro
mulina-polylepis-CCMP1757-20130903_169011_1:0.2132803633,((Pleurochrysis-carterae-CCMP645-
20130926_177674_1:0.5781481282,Prymnesium-parvum-Texoma1-
20131001_22049_1:0.2744531289):0.1247555719,Prymnesium-parvum-Texoma1-
20131001_24982_1:0.2147489663):0.1046073246):0.1750312092,Isochrysis-galbana-CCMP1323-
20130912_17711_1:0.6019072789):0.3306018066,((((Chrysochromulina-polylepis-CCMP1757-
20130903_20923_1:0.3118191386,SEED_Emiliania_huxleyi__Haptophyta__APX-CCP_15881_EhuxAPx-
CcP04:0.2288904514):0.2779760753,((Isochrysis-galbana-CCMP1323-
20130912_18445_1:0.0854435151,SEED_Isochrysis_sp.__Haptophyta__CCP9_13317_IspCcP05:0.0680520645):0.15131
57462,Prymnesium-parvum-Texoma1-20131001_13108_1:0.3485228100):0.0747627605):0.2499460543,((Lotharella-
globosa-CCCM811-20130918_11324_1:0.0858757967,SEED_Bigelowiella_natans__Rhizaria__APX-CCP_11106_BnaAPx-
CcP:0.1463487262):0.5395609427,SEED_Plasmodiophora_brassicae_CEO99391.1:0.3976259403):0.0941353783):0.0601
479943,((((((Dinophysis_acuminata_CAMPEP_0179259890_Dinophyta_Dinophysiales:0.2461304068,((((Haptolina-
ericina_MMETSP1096-20121128_13351_1:0.4594975748,SEED_Thalassiosira_pseudonana__Stramenopila__APX-
CCP_2557_TpsAPx-CcP01:0.1969960349):0.0522318652,Rhizochromulina_marina_MMETSP1173_Gene.18745-
Transcript_9751_Stramenopila_Dictyochophyceae:0.2317188903):0.0498836221,Mesodinium_pulex_MMETSP0467_Ge
ne.77675-
Transcript_52191_Ciliophora_Litostomatea:0.3121950276):0.0645919581,(SEED_Emiliania_huxleyi__Haptophyta__APX-
CCP_13365_EhuxAPx-CcP03:0.3362746919,Scrippsiella-hangoei-SHTV5-
20131105_129681_1:0.1738864532):0.0240715492):0.0866268840):0.0361811865,SEED_Symbiodinium_sp.__Dinoflage
llata__APX-CCP_13187_SymAPx-CcP1_F1:0.4149099201):0.0453891635,SEED_Ochromonas_sp.__Stramenopila__APX-
CCP_13322_OCgrAPx-CcP03:0.1639129504):0.0988179202,Karenia_brevis_MMETSP0027_Gene.36904-
Transcript_30870_Dinophyta_Gymnodiniales:0.2860789491):0.1763099918,((Phaeocystis_MMETSP1162-
20130426_4569_1:0.4638936534,Prymnesium-parvum-Texoma1-
20131001_199463_1:0.5382319950):0.1552764533,SEED_Chrysochromulina_tobin__Haptophyta__APX-
CCP_13471_CtoAPx-CcP01:0.5301052341):0.2296349654):0.2274477386,(Norrisiella-sphaerica_MMETSP0113_2-
20130426_52835_1:0.1826958037,SEED_Bigelowiella_natans__Rhizaria__CCP_2510_BnaCcP01:0.1101447431):0.48719
09323):0.0172340858):0.0719699876):0.0508890675,(SEED_Artemidia_motanka_TRINITY_DN17334_c8_g3_i1:0.23371
38148,SEED_Namystynia_karyoxenos_TRINITY_DN22390_c0_g1_i1:0.1415549914):0.3306232949):0.0278808013):0.135
2451242,((((((SEED_C4B63_47g23-

t42_1_Trypanosoma_cruzi_Dm28c_2018:0.0342790097,SEED_Tc_MARK_3376_Trypanosoma_cruzi_marinkellei:0.01437
49540):0.1364423321,SEED_DQ04_02591010-
t26_1_Trypanosoma_grayi:0.0793711377):0.0580683993,SEED_TM35_000011050-
t36_1_Trypanosoma_theileri:0.1259540826):0.0569719161,SEED_PCON_0074310.mRNA_Paratrypanosoma_confusum:
0.2635188697):0.1056707001,SEED_rna_Baya_117_0180-
1_Blechomonas_ayalai:0.3143218795):0.0803691984,(((((SEED_CFAC1_290028200.1_Crithidia_fasciculata:0.061369718
3,SEED_rna_Lsey_0078_0210-
1_Leptomonas_seymouri:0.0570830122):0.0371841853,SEED_c6407_g1_i1_Crithidia_thermophila:0.0684115909):0.01
13426565,SEED_rna_LpyrH10_05_0250_Leptomonas_pyrrhocoris:0.0957432945):0.0225087988,(SEED_EMOLV88_3400
06000.1_Endotrypanum_monterogeii:0.1578596734,(((SEED_LENLEM3045_340005600.1_Leishmania_enriettii:0.03180
71391,SEED_LMARLEM2494_340005600.1_Leishmania_sp._MAR_LEM2494:0.0498217707):0.0349041917,(SEED_LmjF.
34.0070_Leishmania_major_Friedlin:0.0597597933,SEED_LmxM.33.0070.1_Leishmania_mexicana:0.0415564245):0.046
0357457):0.0273388181,SEED_NODE_82_Novymonas_esmeraldas:0.0820190344):0.0248492218):0.0306888251):0.108
6391229,Strigomonas_culicis_EPY37063.1_L-
ascorbate_peroxidase:0.2098527592):0.0725591740):0.3140564306,SEED_Lacrimia_lanifica_TRINITY_DN11414_c4_g13
_i1:0.4912223954):0.1244791728):0.1308348323,(((((Alexandrium_tamarense_MMETSP0384_Gene.274304-
Transcript_168547_Dinophyta_Gonyaulacales:0.3946360354,((Chlamydomonas-euryale_MMETSP0063_2-
20130828_11317_1:0.3505846317,Polytomella-parva_MMETSP0052_2-
20121109_7164_1:0.3352029811):0.0651694873,Chlamydomonas-euryale_MMETSP0063_2-
20130828_63691_1:0.1589002677):0.0876688266):0.0939544193,((Candida_maltosa_EMG50043.1_hypothetical_prote
in_G210_4953:0.2969706445,Debaryomyces_hansenii_XP_462060.2_DEHA2G12166p:0.4136224034):0.3725589178,(C
hlamydomonas-leiostraca_MMETSP1391-
20130828_9607_1:0.3197047525,(Phytophthora_sojae_XP_009521257.1_hypothetical_protein_PHYSODRAFT_482248:
0.9054243758,Protoceratium-reticulatum_MMETSP0228-
20121227_49520_1:0.2829266507):0.8432552424):0.1157493597):0.1262254858):0.0403573586,(Picochlorum-
oklahomense_MMETSP1161-20130426_1898_1:0.2775435861,(Picocystis_salinarum_MMETSP0807_Transcript_9903-
m.42985_Chlorophyta_prasinophyte_CL_VII:0.2429849951,Stygamoeba-regulata_MMETSP0447-
20121125_18088_1:0.2858025086):0.0479153796):0.0635104463):0.0523015794,(((Aplanochytrium_MMETSP0957_Tra
nscript_11830-m.9960_Stramenopila_Labyrinthulomycetes:0.3588637429,Schizochytrium-aggregatum-ATCC28209-
20130930_104702_1:0.1833204754):0.2136567818,(((((((Chromera_velia_Cvel_2127.t1-
p1_Apicomplexa:0.2881774783,(((Chroomonas-cf.-mesostigmatica_MMETSP0047_2-
20121206_36962_1:0.3882454770,(Karenia-brevis-Wilson-20130916_37417_1:0.2054956124,Noctiluca-
scintillans_MMETSP0253-20130528_95621_1:0.1424027927):0.2446337876):0.1032933676,(((Mantoniella-
antarctica_MMETSP1106-20121128_25042_1:0.1538764879,Micromonas-sp-NEPCC29-
20130920_2226_1:0.1043209519):0.2926228975,Prasinoderma-singularis_MMETSP1315-
20130426_68631_1:0.3162526338):0.1435324727,Prymnesium-parvum-Texoma1-
20131001_10048_1:0.2836386530):0.0393368651):0.1201594479,Oxyrrhis_marina_MMETSP0471_Transcript_28376-
m.41957_Dinophyta_Oxyrrhinales:0.4908498574):0.0879138321):0.2077724418,Ogataea_parapolymorpha_XP_013935
847.1_hypothetical_protein_HPODL_00424:0.8557099751):0.0273330141,Sugiyamaella_lignohabitans_XP_018738120.
1_Ccp1p:0.4850102931):0.0995152706,Rozella_allomycis_EPZ35358.1_Plant_ascorbate_peroxidase_domain-
containing_protein:0.3540831235):0.0110868358,((Fusarium_oxysporum_EGU74407.1_hypothetical_protein_FOXB_15
079:0.2073878724,(Trichophyton_benhamiae_XP_003012241.1_bifunctional_catalase-
peroxidase_Cat2:0.2071127338,xxx_Botrytis_cinerea_EMR84675.1_putative_cytochrome_c_peroxidase_protein:0.1470
613752):0.0668554782):0.2145661596,xxx_Tsuchiyaea_wingfieldii_XP_019030344.1_cytochrome_c_peroxidase:0.3736
187976):0.0501142855):0.0306247847,(Synchroma_pusillum_MMETSP1452_Transcript_7936-
m.19741_Stramenopila_Synchromophyceae:0.4326000063,xxx_Nannochloropsis_gaditana_EWM29691.1_cytochrome_
c_peroxidase:0.5760381092):0.0909820651):0.0096936162,((((Cyberlindnera_fabianii_CDR36592.1:0.4262235040,Pach
ysolen_tannophilus_ODV96415.1_hypothetical_protein_PACTADRAFT_49767:0.4914934992):0.0495276575,xxx_Ascoid
ea_rubescens_XP_020047494.1_heme_peroxidase:0.4061857159):0.1423352557,(xxx_Mucor_ambiguus_GAN10078.1_

cytochrome_c_peroxidase_Ccp1:0.2768337217,xxx_Syncephalastrum_racemosum_ORY95648.1_heme_peroxidase:0.51
69282062):0.2440827336):0.1091324916,(Dactylellina_haptotyla_XP_011116536.1_hypothetical_protein_H072_11173:
0.2343327716,xxx_Aspergillus_udagawae_GAO84407.1_cytochrome_c_peroxidase__mitochondrial:0.2983428265):0.10
88861692):0.0849053495):0.0335629720):0.0170138673,((((Bigelowiella-natans_MMETSP1054-
20121227_11945_1:0.3953149117,Micromonas-pusilla_MMETSP1401-
20130829_1406_1:0.3575994003):0.0307460238,(Chroomonas_cf._mesostigmatica_MMETSP0047_Transcript_38188-
m.63215_Cryptophyta_Pyrenomonadales:0.5656322299,Hemiselmis-andersenii_MMETSP0043_2-
20121206_17536_1:0.2335066119):0.1353223330):0.1994290312,Erythrolobus-madagascarensis_MMETSP1354-
20130828_2272_1:0.3452992071):0.1533092822,Pavlova-lutheri_MMETSP1463-
20131121_85637_1:0.3584389242):0.0520222623):0.0496034107):0.0576194258,((Hemiselmis-tepida_MMETSP1355-
20121228_24535_1:0.3407153703,(SEED_Artemidia_motanka_TRINITY_DN15980_c4_g3_i1:0.2485103545,SEED_Namy
stynia_karyoxenos_TRINITY_DN77678_c0_g1_i1:0.2099694792):0.2373466782):0.0801886117,((((SEED_Diplonema_jap
onicum_TRINITY_DN10300_c4_g1_i2:0.3589844892,SEED_Diplonema_papillatum_TR110952__c20_g26_i1__m.25215:0
.5573200448):0.1164637868,SEED_Lacrimia_lanifica_TRINITY_DN7350_c0_g1_i1:0.2015149014):0.0816468150,SEED_Di
plonema_japonicum_TRINITY_DN10460_c6_g1_i1:0.6217692494):0.1784728353,SEED_Sulcionema_specki_TRINITY_DN
80007_c0_g1_i1:0.2627372732):0.1957374477):0.0359762296):0.1123872761):0.0395786311,(Hemiselmis-
andersenii_MMETSP0043_2-
20121206_19730_1:0.7115808715,xxx_Basidiobolus_meristosporus_ORX96746.1_heme_peroxidase:0.8012166188):0.2
060867501):0.0512122812,((((SEED_Artemidia_motanka_TRINITY_DN14823_c0_g2_i1:1.9799975320,SEED_Namystynia
_karyoxenos_TRINITY_DN89651_c0_g1_i1:0.5586025663):0.6449962249,((SEED_Diplonema_japonicum_TRINITY_DN10
282_c5_g3_i2:1.7089183301,SEED_Lacrimia_lanifica_TRINITY_DN4895_c0_g1_i1:0.6499795321):0.6023067963,SEED_S
ulcionema_specki_TRINITY_DN77967_c0_g1_i1:0.9835455868):0.2953448439):0.1982942712,((SEED_Artemidia_motan
ka_TRINITY_DN6375_c0_g1_i2:0.4919748733,SEED_Namystynia_karyoxenos_TRINITY_DN15097_c0_g1_i1:0.232011093
3):0.2853496216,SEED_Sulcionema_specki_TRINITY_DN23548_c0_g1_i2:0.3780959899):0.9118192544):0.1871486434,(
(((((SEED_Artemidia_motanka_TRINITY_DN24471_c0_g1_i1:0.3101525921,SEED_Namystynia_karyoxenos_TRINITY_DN5
2435_c0_g1_i1:0.3088393514):0.2525126253,SEED_Sulcionema_specki_TRINITY_DN10012_c0_g1_i1:0.5769621105):0.
2224924329,(SEED_Diplonema_japonicum_TRINITY_DN8053_c0_g1_i1:0.3152424652,SEED_Diplonema_papillatum_TR
8739__c0_g1_i1__m.830:0.3071094873):0.0752818023):0.0612374172,SEED_Lacrimia_lanifica_TRINITY_DN8638_c0_g1
_i1:0.5335790780):0.1337988577,SEED_Rhynchopus_humris_TRINITY_DN40212_c0_g1_i1:0.5475506233):0.843543048
4,(((((((SEED_Diplonema_japonicum_TRINITY_DN10642_c2_g3_i1:1.0677218664,SEED_Sulcionema_specki_TRINITY_DN
70017_c0_g1_i1:0.2780614161):0.1255612069,SEED_Diplonema_papillatum_TR45711__c0_g1_i1__m.5672:0.5595834
723):0.0000022146,SEED_Lacrimia_lanifica_TRINITY_DN11235_c0_g1_i6:1.0342145855):0.1457780245,SEED_Lacrimia_l
anifica_TRINITY_DN11643_c1_g8_i2:0.3305463756):0.2632988621,(SEED_Rhynchopus_humris_TRINITY_DN63548_c0_g
1_i1:0.0000023451,SEED_Sulcionema_specki_TRINITY_DN49103_c1_g2_i3:0.0000020772):0.5225223454):0.337125558
4,SEED_Lacrimia_lanifica_TRINITY_DN11361_c1_g9_i2:0.7822948611):0.2878423704,(SEED_Rhynchopus_humris_TRINI
TY_DN23907_c1_g1_i1:0.8269978875,SEED_Rhynchopus_humris_TRINITY_DN57024_c0_g1_i1:0.9709829852):0.88943
67916):0.4902632284):0.1452959348):0.0638294689):0.0622604622,(((Aplanochytrium_MMETSP0954_Transcript_138
63-m.13298_Stramenopila_Labyrinthulomycetes:0.2794162704,Thraustochytrium-sp-LLF1b-
20130905_75161_1:0.2664208160):0.0870635309,(Durinskia-baltica-CSIRO_CS-38-
20140214_139959_1:0.4340835557,Exanthemachrysis_gayraliae_MMETSP1464_Transcript_20828-
m.51128_Haptophyta_Pavlovales:0.3217449295):0.0770405286):0.2067844259,Tetrahymena_thermophila_EAS01806_
Ciliata_Oligohymenophorea:0.6112365599):0.2051293920):0.1398580513,(((((SEED_Euglena_gracilis_GDJR01082147.1:
0.0091837432,SEED_Euglena_gracilis_GEFR01009177.1:0.0030612601):0.0736777836,SEED_Euglena_gracilis_GDJR0108
2152.1:0.0657510340):0.0293354690,SEED_Euglena_gracilis_GEFR01013723.1:0.0702745706):0.0233927615,SEED_Eugl
ena_gracilis_GEFR01042768.1:0.0459844183):0.0716424266,SEED_Euglena_longa_Contig45548:0.0170398498):0.77295
24780):0.2784861030,(Goniomonas-pacifica-CCMP1869-
20130911_167160_1:0.6129469780,((SEED_Diplonema_japonicum_TRINITY_DN8646_c0_g2_i1:0.3901908979,SEED_Di
plonema_papillatum_TR130880__c10_g1_i1__m.31721:0.6313135691):0.1755021220,(SEED_Namystynia_karyoxenos_

TRINITY_DN26871_c0_g1_i1:0.3884999283,SEED_Sulcionema_specki_TRINITY_DN50008_c0_g1_i1:0.5064353222):0.08
65739924):0.0880238012):0.1655910784)OROOT;

File S2: Maximum-likelihood phylogenetic tree of Eulgenozoa CAT in Newick format.

(Diplonema japonicum 1604_TRINITY_DN11173_c4_g2_i1:0.0713974632,(Diplonema japonicum 1604_TRINITY_DN11173_c4_g3_i1:0.0000023247,Diplonema japonicum 1604_TRINITY_DN11173_c4_g3_i2:0.0000023247)100:0.0276455492,(Rhynchopus humris 1608_TRINITY_DN23544_c0_g1_i1:0.2733067094,((((Artemidia motanka 1610_TRINITY_DN17294_c8_g2_i1:0.0921283120,Namystynia karyoxenos 1621_TRINITY_DN57307_c0_g1_i1:0.1226241664)100:0.3398950739,Sulcionema specki 1618_TRINITY_DN284_c0_g1_i1:0.3976283462)100:0.2904343898,(((Diplonema papillatum DIPPA_00897.mRNA.1:0.0000000000,Diplonema papillatum DIPPA_00904.mRNA.1:0.0000000000):0.0000000000,Diplonema papillatum DIPPA_00901.mRNA.1:0.0000000000):0.0000022331,Diplonema papillatum DIPPA_00900.mRNA.1:0.0000028358)100:0.3735038339,((((((Fulvimarina pelagi @Fulvimarina_Aurantimonadaceae_Rhizobiales_Alphaproteobacteria_Proteobacteria_Bacteria WP_007068307:0.1613464657,Notoacmeibacter marinus @Notoacmeibacter_Notoacmeibacteraceae_Rhizobiales_Alphaproteobacteria_Proteobacteria_Bacteria WP_094075414:0.0780824844)100:0.1424227855,(Henriciella algicola @Henriciella_Hyphomonadaceae_Rhodobacterales_Alphaproteobacteria_Proteobacteria_Bacteria WP_119452489:0.0618716795,Henriciella aquimarina @Henriciella_Hyphomonadaceae_Rhodobacterales_Alphaproteobacteria_Proteobacteria_Bacteria WP_084396704:0.0746785559)41:0.0000022460)90:0.0316005220,Henriciella litoralis @Henriciella_Hyphomonadaceae_Rhodobacterales_Alphaproteobacteria_Proteobacteria_Bacteria WP_084417947:0.0994450417)100:0.0344748805,(Afifella pfennigii @Afifella_Rhodobiaceae_Rhizobiales_Alphaproteobacteria_Proteobacteria_Bacteria WP_026378988:0.0721710588,Acuticoccus kandeliae @Acuticoccus_Rhodobacteraceae_Rhodobacterales_Alphaproteobacteria_Proteobacteria_Bacteria WP_108661610:0.0648939940)54:0.0294813177)53:0.0253741086,Pararhizobium haloflavum @Pararhizobium_Rhizobium/Agrobacterium group_Rhizobiaceae_Rhizobiales_Alphaproteobacteria_Proteobacteria_Bacteria WP_099865259:0.1098414430)84:0.0144948880,Hirschia sp. @Hirschia_Hyphomonadaceae_Rhodobacterales_Alphaproteobacteria_Proteobacteria_Bacteria MBB34783:0.1173966329)95:0.0352536494)98:0.0505747318,Maritimibacter sp. @Maritimibacter_Rhodobacteraceae_Rhodobacterales_Alphaproteobacteria_Proteobacteria_Bacteria MAQ82580:0.1258301459)100:0.0750483495,(Bartonella apis @Bartonella_Bartonellaceae_Rhizobiales_Alphaproteobacteria_Proteobacteria_Bacteria WP_075870246:0.1413543555,Microbulbifer thermotolerans @Microbulbifer_Microbulbiferaceae_Cellvibrionales_Gammaproteobacteria_Proteobacteria_Bacteria WP_067149864:0.1887469458)67:0.0419655575)58:0.0203991220,((Nitratireductor sp. @Nitratireductor_Phyllobacteriaceae_Rhizobiales_Alphaproteobacteria_Proteobacteria_Bacteria MAS14710:0.0157045288,Nitratireductor aquibiodomus @Nitratireductor_Phyllobacteriaceae_Rhizobiales_Alphaproteobacteria_Proteobacteria_Bacteria WP_065816189:0.0236817282)100:0.0288127432,Nitratireductor indicus @Nitratireductor_Phyllobacteriaceae_Rhizobiales_Alphaproteobacteria_Proteobacteria_Bacteria WP_009452749:0.0337066649)100:0.0554085187)84:0.0450536975,(Aureimonas altamirensis @Aureimonas_Aurantimonadaceae_Rhizobiales_Alphaproteobacteria_Proteobacteria_Bacteria WP_039195226:0.1224910958,Ensifer adhaerens @Ensifer_Sinorhizobium/Ensifer group_Rhizobiaceae_Rhizobiales_Alphaproteobacteria_Proteobacteria_Bacteria WP_065372734:0.1563274774)54:0.0243764909)45:0.0216309904,((Actibacterium atlanticum @Actibacterium_Rhodobacteraceae_Rhodobacterales_Alphaproteobacteria_Proteobacteria_Bacteria WP_035247661:0.1683631644,Brevundimonas naejangsanensis

@Brevundimonas_Caulobacteraceae_Caulobacterales_Alphaproteobacteria_Proteobacteria_Bacteria
WP_024354401:0.1246740940)61:0.0482175958,(((((((Albimonas pacifica
@Albimonas_Rhodobacteraceae_Rhodobacterales_Alphaproteobacteria_Proteobacteria_Bacteria
WP_092861411:0.3926947441,Pseudoxanthomonas mexicana
@Pseudoxanthomonas_Xanthomonadaceae_Xanthomonadales_Gammaproteobacteria_Proteobacteria_Bacteria
WP_062358294:0.1394894861)89:0.0177976073,Roseovarius mucosus
@Roseovarius_Rhodobacteraceae_Rhodobacterales_Alphaproteobacteria_Proteobacteria_Bacteria
WP_081507981:0.1056014722)75:0.0148726621,(Mesorhizobium alhagi
@Mesorhizobium_Phyllobacteriaceae_Rhizobiales_Alphaproteobacteria_Proteobacteria_Bacteria
WP_008840002:0.0328760010,Pannonibacter phragmitetus
@Pannonibacter_Rhodobacteraceae_Rhodobacterales_Alphaproteobacteria_Proteobacteria_Bacteria
WP_094462707:0.0495648406)100:0.0437370165)95:0.0160369365,Palleronia marisminoris
@Palleronia_Rhodobacteraceae_Rhodobacterales_Alphaproteobacteria_Proteobacteria_Bacteria
SLN67837:0.0871250839)100:0.1364231304,Maritalea myrionectae
@Maritalea_Hyphomicrobiaceae_Rhizobiales_Alphaproteobacteria_Proteobacteria_Bacteria
AVX03940:0.1251679974)67:0.0335307021,(Phyllobacterium zundukense
@Phyllobacterium_Phyllobacteriaceae_Rhizobiales_Alphaproteobacteria_Proteobacteria_Bacteria
WP_099999878:0.1021084567,(Rhodobacteraceae bacterium D4M1
@Rhodobacteraceae_Rhodobacterales_Alphaproteobacteria_Proteobacteria_Bacteria
WP_138578173:0.0264006789,Oceanicella sp. SM1341
@Oceanicella_Rhodobacteraceae_Rhodobacterales_Alphaproteobacteria_Proteobacteria_Bacteria
WP_118132613:0.0129604690)100:0.1383608824)32:0.0402593038)25:0.0345914485,Pelagibacterium luteolum
@Pelagibacterium_Hyphomicrobiaceae_Rhizobiales_Alphaproteobacteria_Proteobacteria_Bacteria
WP_090594022:0.1180902513)42:0.0133034076,((((Aquamicrobium defluvii
@Aquamicrobium_Phyllobacteriaceae_Rhizobiales_Alphaproteobacteria_Proteobacteria_Bacteria
WP_035025201:0.1453188083,(Neorhizobium alkalisoli @Neorhizobium_Rhizobium/Agrobacterium
group_Rhizobiaceae_Rhizobiales_Alphaproteobacteria_Proteobacteria_Bacteria
WP_105381577:0.0856691933,Pseudorhizobium pelagicum @Pseudorhizobium_Rhizobium/Agrobacterium
group_Rhizobiaceae_Rhizobiales_Alphaproteobacteria_Proteobacteria_Bacteria
WP_037161304:0.0581967101)87:0.0076929532)93:0.0371983992,(Rhizobium rhizosphaerae
@Rhizobium_Rhizobium/Agrobacterium
group_Rhizobiaceae_Rhizobiales_Alphaproteobacteria_Proteobacteria_Bacteria
WP_081176475:0.0190918760,Sinorhizobium arboris @Sinorhizobium_Sinorhizobium/Ensifer
group_Rhizobiaceae_Rhizobiales_Alphaproteobacteria_Proteobacteria_Bacteria
WP_027999591:0.1320114517)88:0.0407858753)93:0.0491086459,Mycoplana dimorpha
@Mycoplana_Brucellaceae_Rhizobiales_Alphaproteobacteria_Proteobacteria_Bacteria
PTM98349:0.0882366766)53:0.0227753147,Martelella endophytica
@Martelella_Aurantimonadaceae_Rhizobiales_Alphaproteobacteria_Proteobacteria_Bacteria
WP_045681354:0.1123233874)78:0.0330441685)23:0.0149078799)38:0.0111150008)53:0.0057636940,(Nitratireductor
basaltis @Nitratireductor_Phyllobacteriaceae_Rhizobiales_Alphaproteobacteria_Proteobacteria_Bacteria
WP_036482110:0.0885717127,Chelativorans sp. J32
@Chelativorans_Phyllobacteriaceae_Rhizobiales_Alphaproteobacteria_Proteobacteria_Bacteria
WP_028034089:0.0499616474)100:0.0461324278)98:0.0443051224,((Acinetobacter nectaris
@Acinetobacter_Moraxellaceae_Pseudomonadales_Gammaproteobacteria_Proteobacteria_Bacteria
WP_023273437:0.2988684766,Hyphomicrobium facile
@Hyphomicrobium_Hyphomicrobiaceae_Rhizobiales_Alphaproteobacteria_Proteobacteria_Bacteria
WP_092864409:0.1408598676)78:0.0469654774,(((Bosea thiooxidans
@Bosea_Bradyrhizobiaceae_Rhizobiales_Alphaproteobacteria_Proteobacteria_Bacteria
WP_055728743:0.0569243015,((Nitratireductor pacificus

@Nitratireductor_Phyllobacteriaceae_Rhizobiales_Alphaproteobacteria_Proteobacteria_Bacteria
WP_008597406:0.0326794109,Nitratireductor soli
@Nitratireductor_Phyllobacteriaceae_Rhizobiales_Alphaproteobacteria_Proteobacteria_Bacteria
WP_048648991:0.0100758986)100:0.0371618206,((Pseudaminobacter salicylatoxidans
@Pseudaminobacter_Phyllobacteriaceae_Rhizobiales_Alphaproteobacteria_Proteobacteria_Bacteria
WP_019173069:0.0040840691,Pseudaminobacter salicylatoxidans
@Pseudaminobacter_Phyllobacteriaceae_Rhizobiales_Alphaproteobacteria_Proteobacteria_Bacteria
WP_109614061:0.0000023247)100:0.0585121647,Bosea vaviloviae
@Bosea_Bradyrhizobiaceae_Rhizobiales_Alphaproteobacteria_Proteobacteria_Bacteria
WP_054208215:0.0609191463)91:0.0460733559)60:0.0140846627)92:0.0236359935,Cucumibacter marinus
@Cucumibacter_Hyphomicrobiaceae_Rhizobiales_Alphaproteobacteria_Proteobacteria_Bacteria
WP_029040759:0.0871748788)95:0.0255384098,(Brucella canis
@Brucella_Brucellaceae_Rhizobiales_Alphaproteobacteria_Proteobacteria_Bacteria
WP_006253319:0.0535018498,Ochrobactrum rhizosphaerae
@Ochrobactrum_Brucellaceae_Rhizobiales_Alphaproteobacteria_Proteobacteria_Bacteria
WP_094573666:0.0820444762)100:0.0367769173)100:0.0524737564)57:0.0174955735)90:0.0262991194,((Sphingobiu
m yanoikuyae @Sphingobium_Sphingomonadaceae_Sphingomonadales_Alphaproteobacteria_Proteobacteria_Bacteria
WP_037518597:0.0593106297,Sphingomonas sanxanigenens
@Sphingomonas_Sphingomonadaceae_Sphingomonadales_Alphaproteobacteria_Proteobacteria_Bacteria
WP_025294563:0.1215640425)100:0.0618493149,Xanthobacter autotrophicus
@Xanthobacter_Xanthobacteraceae_Rhizobiales_Alphaproteobacteria_Proteobacteria_Bacteria
WP_012114341:0.1064632221)92:0.0280559489)99:0.0156359880,(Arboriscoccus pini
@Arboricoccus_Geminicoccaceae_Rhodospirillales_Alphaproteobacteria_Proteobacteria_Bacteria
WP_088561180:0.1831336503,Methylobrevis pamukkalensis
@Methylobrevis_Rhizobiales_Alphaproteobacteria_Proteobacteria_Bacteria
WP_069308299:0.1056308847)100:0.0684094072)100:0.0456166779,Gimesia maris
@Gimesia_Planctomycetaceae_Planctomycetales_Planctomycetia_Planctomycetes_Bacteria
WP_002644801:0.1369008039)98:0.0252989199,(Desulfonatronovibrio magnus
@Desulfonatronovibrio_Desulfohalobiaceae_Desulfovibrionales_Deltaproteobacteria_Proteobacteria_Bacteria
WP_045214586:0.0594885374,Desulfonatronum thiosulfatophilum
@Desulfonatronum_Desulfonatronaceae_Desulfovibrionales_Deltaproteobacteria_Proteobacteria_Bacteria
WP_092116947:0.1078025842)100:0.0691696922)97:0.0256110431,Parachlamydia acanthamoebae
@Parachlamydia_Parachlamydiaceae_Parachlamydiales_Chlamydiae_Bacteria
KIA76750:0.1952131212)81:0.0276169043,(Berkiella cookevillensis @Candidatus
Berkiella_Coxiellaceae_Legionellales_Gammaproteobacteria_Proteobacteria_Bacteria
WP_057624084:0.1606049457,Legionella fallonii
@Legionella_Legionellaceae_Legionellales_Gammaproteobacteria_Proteobacteria_Bacteria
WP_045095009:0.2402427574)95:0.0891080003)55:0.0176627888,(Fangia hongkongensis
@Fangia_Thiotrichales_Gammaproteobacteria_Proteobacteria_Bacteria
WP_018300546:0.2370510407,((Marinimicrobium agarilyticum
@Marinimicrobium_Cellvibrionaceae_Cellvibrionales_Gammaproteobacteria_Proteobacteria_Bacteria
WP_027328912:0.1160332050,Pseudomonas xinjiangensis
@Pseudomonas_Pseudomonadaceae_Pseudomonadales_Gammaproteobacteria_Proteobacteria_Bacteria
WP_093395539:0.1546222878)94:0.0145515273,(Pseudoalteromonas elyakovii
@Pseudoalteromonas_Pseudoalteromonadaceae_Alteromonadales_Gammaproteobacteria_Proteobacteria_Bacteria
WP_039495766:0.2609347219,Tistlia consotensis
@Tistlia_Rhodospirillaceae_Rhodospirillales_Alphaproteobacteria_Proteobacteria_Bacteria
WP_085122001:0.1146724188)95:0.0651546206)90:0.0301893441)66:0.0508571431)95:0.0229499441,((((((Acetobact
er nitrogenifigens @Acetobacter_Acetobacteraceae_Rhodospirillales_Alphaproteobacteria_Proteobacteria_Bacteria

WP_026397660:0.2217803179,(Caenispirillum salinarum
@Caenispirillum_Rhodospirillaceae_Rhodospirillales_Alphaproteobacteria_Proteobacteria_Bacteria
WP_009539914:0.2281400726,Sphingomonas haloaromaticamans
@Sphingomonas_Sphingomonadaceae_Sphingomonadales_Alphaproteobacteria_Proteobacteria_Bacteria
WP_070932012:0.1868134329)100:0.0309784187)99:0.0513132258,((Aurantimonas coralicida
@Aurantimonas_Aurantimonadaceae_Rhizobiales_Alphaproteobacteria_Proteobacteria_Bacteria
WP_024349126:0.1208714182,Aureimonas jatrophae
@Aurantimonas_Aurantimonadaceae_Rhizobiales_Alphaproteobacteria_Proteobacteria_Bacteria
WP_090671893:0.1194418525)100:0.0655362924,Loktanella cinnabarina
@Limimaricola_Rhodobacteraceae_Rhodobacterales_Alphaproteobacteria_Proteobacteria_Bacteria
WP_099273163:0.0946281617)100:0.2463568520)27:0.0148914314,((Cereibacter changlensis
@Cereibacter_Rhodobacteraceae_Rhodobacterales_Alphaproteobacteria_Proteobacteria_Bacteria
WP_107663360:0.1230281869,((Defluviimonas alba
@Defluviimonas_Rhodobacteraceae_Rhodobacterales_Alphaproteobacteria_Proteobacteria_Bacteria
WP_066812982:0.1775452904,Paracoccus tibetensis
@Paracoccus_Rhodobacteraceae_Rhodobacterales_Alphaproteobacteria_Proteobacteria_Bacteria
WP_090743354:0.1823389950)100:0.0770829860,Rhodobacter megalophilus
@Rhodobacter_Rhodobacteraceae_Rhodobacterales_Alphaproteobacteria_Proteobacteria_Bacteria
WP_089256123:0.1178760536)41:0.0215593220)100:0.0688925419,Woodsholea maritima
@Woodsholea_Caulobacteraceae_Caulobacterales_Alphaproteobacteria_Proteobacteria_Bacteria
WP_019962139:0.1919455785)100:0.0539308021)68:0.0269850862,((Asticcacaulis taihuensis
@Asticcacaulis_Caulobacteraceae_Caulobacterales_Alphaproteobacteria_Proteobacteria_Bacteria
WP_090645643:0.1983192570,Hirschia baltica
@Hirschia_Hyphomonadaceae_Rhodobacterales_Alphaproteobacteria_Proteobacteria_Bacteria
WP_015826389:0.2411430295)99:0.0451085500,(Phaeospirillum fulvum
@Phaeospirillum_Rhodospirillaceae_Rhodospirillales_Alphaproteobacteria_Proteobacteria_Bacteria
WP_021132951:0.1614643508,Rhodomicrobium vannielii
@Rhodomicrobium_Hyphomicrobiaceae_Rhizobiales_Alphaproteobacteria_Proteobacteria_Bacteria
WP_013417993:0.1845519710)20:0.0460557705)33:0.0198810041)53:0.0125111291,(((((Aliiglaciecola lipolytica
@Aliiglaciecola_Alteromonadaceae_Alteromonadales_Gammaproteobacteria_Proteobacteria_Bacteria
WP_008845795:0.1010826878,(Paraglaciecola mesophila
@Paraglaciecola_Alteromonadaceae_Alteromonadales_Gammaproteobacteria_Proteobacteria_Bacteria
WP_006992680:0.0000023268,Pseudoalteromonas atlantica
@Pseudoalteromonas_Pseudoalteromonadaceae_Alteromonadales_Gammaproteobacteria_Proteobacteria_Bacteria
WP_011573396:0.0260570425)100:0.1167988408)100:0.0442629002,Salinimonas chungwhensis
@Salinimonas_Alteromonadaceae_Alteromonadales_Gammaproteobacteria_Proteobacteria_Bacteria
WP_018982481:0.1430855784)99:0.0211093504,Alteromonas gracilis
@Alteromonas_Alteromonadaceae_Alteromonadales_Gammaproteobacteria_Proteobacteria_Bacteria
WP_105929773:0.1322950331)33:0.0210194799,Loktanella hongkongensis
@Limimaricola_Rhodobacteraceae_Rhodobacterales_Alphaproteobacteria_Proteobacteria_Bacteria
WP_017928526:0.1473881075)62:0.0125008904,((((((((Aquimixticola soesokkakensis
@Aquimixticola_Rhodobacteraceae_Rhodobacterales_Alphaproteobacteria_Proteobacteria_Bacteria
WP_085836344:0.1071600818,Yangia pacifica
@Yangia_Rhodobacteraceae_Rhodobacterales_Alphaproteobacteria_Proteobacteria_Bacteria
WP_092426298:0.0408069173)100:0.0471518874,Citreicella marina
@Salipiger_Rhodobacteraceae_Rhodobacterales_Alphaproteobacteria_Proteobacteria_Bacteria
WP_089851377:0.0719175617)87:0.0433478018,Poseidonocella pacifica
@Poseidonocella_Rhodobacteraceae_Rhodobacterales_Alphaproteobacteria_Proteobacteria_Bacteria
WP_092066816:0.0591876196)97:0.0358611002,(Celeribacter manganoxidans

@Celeribacter_Rhodobacteraceae_Rhodobacterales_Alphaproteobacteria_Proteobacteria_Bacteria
WP_097373382:0.0771942346,Sulfitobacter dubius
@Sulfitobacter_Rhodobacteraceae_Rhodobacterales_Alphaproteobacteria_Proteobacteria_Bacteria
WP_093926369:0.1737305619)100:0.0254598713)100:0.0906294998,Sagittula stellata
@Sagittula_Rhodobacteraceae_Rhodobacterales_Alphaproteobacteria_Proteobacteria_Bacteria
WP_005860934:0.2171896972)45:0.0717737676,(Puniceibacterium antarcticum
@Puniceibacterium_Rhodobacteraceae_Rhodobacterales_Alphaproteobacteria_Proteobacteria_Bacteria
WP_099912457:0.1990466455,Thioclava dalianensis
@Thioclava_Rhodobacteraceae_Rhodobacterales_Alphaproteobacteria_Proteobacteria_Bacteria
WP_038065075:0.1836294594)42:0.0196853581)44:0.0258522518,(((Citreicella thiooxidans
@Salipiger_Rhodobacteraceae_Rhodobacterales_Alphaproteobacteria_Proteobacteria_Bacteria
WP_089958747:0.0906928287,Pelagibaca bermudensis
@Salipiger_Rhodobacteraceae_Rhodobacterales_Alphaproteobacteria_Proteobacteria_Bacteria
WP_007793566:0.0288712255)100:0.0268512679,Thiobacimonas profunda
@Salipiger_Rhodobacteraceae_Rhodobacterales_Alphaproteobacteria_Proteobacteria_Bacteria
WP_093411896:0.1399862676)90:0.0361705464,(Roseovarius confluentis
@Roseovarius_Rhodobacteraceae_Rhodobacterales_Alphaproteobacteria_Proteobacteria_Bacteria
WP_103761423:0.1295631816,Sulfitobacter noctilucicola
@Sulfitobacter_Rhodobacteraceae_Rhodobacterales_Alphaproteobacteria_Proteobacteria_Bacteria
KIN63619:0.1749709080)89:0.0111685409)100:0.1503133266)98:0.0236569212,Oceaniovalibus guishaninsula JLT2003
@Oceaniovalibus_Rhodobacteraceae_Rhodobacterales_Alphaproteobacteria_Proteobacteria_Bacteria
EKE43500:0.1584551925)100:0.0128572826,Roseivivax halodurans
@Roseivivax_Rhodobacteraceae_Rhodobacterales_Alphaproteobacteria_Proteobacteria_Bacteria
WP_037257332:0.2206359192)66:0.0496674117)100:0.2711726270,(((Apibacter mensalis
@Apibacter_Flavobacteriaceae_Flavobacteriales_Flavobacteriia_Bacteroidetes_Bacteria
WP_055424863:0.2157898766,Pustulibacterium marinum
@Pustulibacterium_Flavobacteriaceae_Flavobacteriales_Flavobacteriia_Bacteroidetes_Bacteria
WP_093025324:0.1621681959)97:0.0709697709,(((Cellulophaga lytica
@Cellulophaga_Flavobacteriaceae_Flavobacteriales_Flavobacteriia_Bacteroidetes_Bacteria
WP_038506872:0.0767432070,Zobellia uliginosa
@Zobellia_Flavobacteriaceae_Flavobacteriales_Flavobacteriia_Bacteroidetes_Bacteria
WP_038236162:0.0834638026)100:0.0314402969,Leeuwenhoekiella blandensis
@Leeuwenhoekiella_Flavobacteriaceae_Flavobacteriales_Flavobacteriia_Bacteroidetes_Bacteria
WP_009781700:0.2205639119)100:0.0200931030,Crocinitomix catalasitica
@Crocinitomix_Crocinitomicaceae_Flavobacteriales_Flavobacteriia_Bacteroidetes_Bacteria
WP_027418306:0.1351278083)96:0.0178215909)82:0.0114305146,Aquimarina muelleri
@Aquimarina_Flavobacteriaceae_Flavobacteriales_Flavobacteriia_Bacteroidetes_Bacteria
WP_027411424:0.1361114414)88:0.0896716113)97:0.1382995592)95:0.0208837148,(((Azospirillum halopraeferens
@Azospirillum_Rhodospirillaceae_Rhodospirillales_Alphaproteobacteria_Proteobacteria_Bacteria
WP_035691716:0.0786992162,Niveispirillum irakense
@Niveispirillum_Rhodospirillaceae_Rhodospirillales_Alphaproteobacteria_Proteobacteria_Bacteria
WP_029012920:0.0614554142)100:0.0436721275,Nitrospirillum amazonense
@Nitrospirillum_Rhodospirillaceae_Rhodospirillales_Alphaproteobacteria_Proteobacteria_Bacteria
WP_004272833:0.1288090935)99:0.0510830817,(((Elstera cyanobacteriorum
@Elstera_Rhodospirillaceae_Rhodospirillales_Alphaproteobacteria_Proteobacteria_Bacteria
WP_094408407:0.1109074785,Rhodospirillum centenum
@Rhodospirillum_Rhodospirillaceae_Rhodospirillales_Alphaproteobacteria_Proteobacteria_Bacteria
WP_012568679:0.1326573465)84:0.0371704924,(Enhygromyxa salina
@Enhygromyxa_Myxococcales_Deltaproteobacteria_Proteobacteria_Bacteria PRP95154:0.2436671194,(Lautropia

mirabilis @Lautropia_Burkholderiaceae_Burkholderiales_Betaproteobacteria_Proteobacteria_Bacteria WP_083799446:0.1952217437,(Mitsuaria chitosanitabida @Mitsuaria_Burkholderiales_Betaproteobacteria_Proteobacteria_Bacteria WP_067066110:0.0672379133,Roseateles depolymerans @Roseateles_Burkholderiales_Betaproteobacteria_Proteobacteria_Bacteria WP_058933257:0.0615116423)100:0.1029929909)20:0.0039646424)82:0.0230733997)70:0.0208147645,(Leptonema illini @Leptonema_Leptospiraceae_Leptospirales_Spirochaetes_Bacteria WP_002775558:0.1150727527,(Tepidiphilus margaritifer @Tepidiphilus_Hydrogenophilaceae_Hydrogenophilales_Hydrogenophilalia_Proteobacteria_Bacteria WP_028874022:0.2323455736,Thorsellia anophelis @Thorsellia_Thorselliaceae_Enterobacterales_Gammaproteobacteria_Proteobacteria_Bacteria WP_093320748:0.1741141860)83:0.0206695242)98:0.0696750152)76:0.0253912516)93:0.0183130048)100:0.0397427060,(Neptunomonas japonica @Neptunomonas_Oceanospirillales_Gammaproteobacteria_Proteobacteria_Bacteria WP_028469634:0.1434916304,(Saccharophagus degradans @Saccharophagus_Cellvibrionaceae_Cellvibrionales_Gammaproteobacteria_Proteobacteria_Bacteria WP_011467313:0.0986705640,Terasakiispira papahanaumokuakeensis @Terasakiispira_unclassified Oceanospirillales_Oceanospirillales_Gammaproteobacteria_Proteobacteria_Bacteria WP_068997014:0.1585884154)79:0.0410285808)100:0.0564000229)98:0.0252864959)84:0.0209862941,Pleomorphomonas oryzae @Pleomorphomonas_Methylocystaceae_Rhizobiales_Alphaproteobacteria_Proteobacteria_Bacteria WP_026792454:0.1353188965)90:0.0250532314,(Estrella lausannensis @Estrella_Criblamydiaceae_Parachlamydiales_Chlamydiae_Bacteria WP_098038142:0.2045816356,Lysobacter spongiicola @Lysobacter_Xanthomonadaceae_Xanthomonadales_Gammaproteobacteria_Proteobacteria_Bacteria WP_078757159:0.3102800927)99:0.0425415104)87:0.0169396798,(Hyphomicrobium denitrificans @Hyphomicrobium_Hyphomicrobiaceae_Rhizobiales_Alphaproteobacteria_Proteobacteria_Bacteria WP_013216473:0.1763541728,((Hyphomicrobium sulfonivorans @Hyphomicrobium_Hyphomicrobiaceae_Rhizobiales_Alphaproteobacteria_Proteobacteria_Bacteria WP_068462615:0.3020530026,Nesiotobacter exalbescens @Nesiotobacter_Rhodobacteraceae_Rhodobacterales_Alphaproteobacteria_Proteobacteria_Bacteria WP_028480076:0.1212641002)97:0.0826810638,Mongoliimonas terrestris @Mongoliimonas_Rhizobiales_Alphaproteobacteria_Proteobacteria_Bacteria WP_075214554:0.1706506079)90:0.0388043015)85:0.0150738582)100:0.0744740379,Kaistia soli @Kaistia_Rhizobiaceae_Rhizobiales_Alphaproteobacteria_Proteobacteria_Bacteria WP_073052405:0.1387744950)100:0.0715206398,((((((Acetobacter indonesiensis @Acetobacter_Acetobacteraceae_Rhodospirillales_Alphaproteobacteria_Proteobacteria_Bacteria WP_048844948:0.0825424587,((((Acidomonas methanolica @Acidomonas_Acetobacteraceae_Rhodospirillales_Alphaproteobacteria_Proteobacteria_Bacteria WP_042055227:0.1602272467,Neoasaia chiangmaiensis @Neoasaia_Acetobacteraceae_Rhodospirillales_Alphaproteobacteria_Proteobacteria_Bacteria WP_077808070:0.1626696438)92:0.0192876327,((Bombella intestini @Bombella_Acetobacteraceae_Rhodospirillales_Alphaproteobacteria_Proteobacteria_Bacteria WP_077396864:0.0368832555,Parasaccharibacter apium @Parasaccharibacter_Acetobacteraceae_Rhodospirillales_Alphaproteobacteria_Proteobacteria_Bacteria WP_099026622:0.0442033696)100:0.1487417897,Saccharibacter floricola @Saccharibacter_Acetobacteraceae_Rhodospirillales_Alphaproteobacteria_Proteobacteria_Bacteria WP_018979905:0.1137026535)100:0.1173498522)97:0.0232310989,Gluconobacter frateurii @Gluconobacter_Acetobacteraceae_Rhodospirillales_Alphaproteobacteria_Proteobacteria_Bacteria WP_010504439:0.0977653602)97:0.0257512956,Tanticharoenia sakaeratensis @Tanticharoenia_Acetobacteraceae_Rhodospirillales_Alphaproteobacteria_Proteobacteria_Bacteria WP_048849766:0.1117188195)100:0.1165100170)100:0.1216298662,Granulibacter bethesdensis @Granulibacter_Acetobacteraceae_Rhodospirillales_Alphaproteobacteria_Proteobacteria_Bacteria AHJ63856:0.0816112810)100:0.1169938268,Roseomonas deserti

@Roseomonas_Acetobacteraceae_Rhodospirillales_Alphaproteobacteria_Proteobacteria_Bacteria
WP_076959326:0.1057579036)90:0.0321249258,((Geminicoccus roseus
@Geminicoccus_Geminicoccaceae_Rhodospirillales_Alphaproteobacteria_Proteobacteria_Bacteria
WP_027133691:0.1773181498,(Microvirga vignae
@Microvirga_Methylobacteriaceae_Rhizobiales_Alphaproteobacteria_Proteobacteria_Bacteria
WP_047188237:0.0925246327,Prosthecomicrobium hirschii
@Prosthecomicrobium_Hyphomicrobiaceae_Rhizobiales_Alphaproteobacteria_Proteobacteria_Bacteria
WP_054360216:0.0778440183)99:0.0240114456)100:0.0393140249,Methyloversatilis thermotolerans
@Methyloversatilis_Sterolibacteriaceae_Nitrosomonadales_Betaproteobacteria_Proteobacteria_Bacteria
WP_018411871:0.2346371946)93:0.0668599289)98:0.0571096675,Caulobacter henricii
@Caulobacter_Caulobacteraceae_Caulobacterales_Alphaproteobacteria_Proteobacteria_Bacteria
WP_062149950:0.2120705067)99:0.0769091272,(((((Arcobacter canalis @Arcobacter_Arcobacter
group_Campylobacteraceae_Campylobacterales_Epsilonproteobacteria_Proteobacteria_Bacteria
WP_099333861:0.2246720927,(Campylobacter fetus
@Campylobacter_Campylobacteraceae_Campylobacterales_Epsilonproteobacteria_Proteobacteria_Bacteria
WP_065842202:0.2373597164,Helicobacter rodentium
@Helicobacter_Helicobacteraceae_Campylobacterales_Epsilonproteobacteria_Proteobacteria_Bacteria
WP_026943187:0.2418216093)100:0.1398818527)100:0.1327647041,Sulfurospirillum barnesii
@Sulfurospirillum_Campylobacteraceae_Campylobacterales_Epsilonproteobacteria_Proteobacteria_Bacteria
WP_014770651:0.1658082988)100:0.0673935685,Pseudoxanthobacter soli
@Pseudoxanthobacter_Xanthobacteraceae_Rhizobiales_Alphaproteobacteria_Proteobacteria_Bacteria
WP_073625790:0.2075769168)99:0.0661690294,(Prosthecobacter debontii
@Prosthecobacter_Verrucomicrobiaceae_Verrucomicrobiales_Verrucomicrobiae_Verrucomicrobia_Bacteria
WP_078812524:0.0781204925,Rubritalea squalenifaciens DSM 18772
@Rubritalea_Rubritaleaceae_Verrucomicrobiales_Verrucomicrobiae_Verrucomicrobia_Bacteria
SHI66560:0.1496174649)100:0.0913410885)99:0.0350893446,(Methylocella silvestris
@Methylocella_Beijerinckiaceae_Rhizobiales_Alphaproteobacteria_Proteobacteria_Bacteria
WP_012591463:0.1822753455,Oceanibaculum pacificum
@Oceanibaculum_Rhodospirillaceae_Rhodospirillales_Alphaproteobacteria_Proteobacteria_Bacteria
WP_067551728:0.0920990229)67:0.0388565737)61:0.0196023442)98:0.0338014356)99:0.0466332105,((Anaerolinea
thermolimosa @Anaerolinea_Anaerolineaceae_Anaerolineales_Anaerolineae_Chloroflexi_Bacteria
WP_062192507:0.0532741226,Ignavibacteria bacterium @Ignavibacteria_Ignavibacteriae_Bacteria
PIS45166:0.2473021924)100:0.0632233417,(Herpetosiphon geysericola
@Herpetosiphon_Herpetosiphonaceae_Herpetosiphonales_Chloroflexia_Chloroflexi_Bacteria
WP_054536643:0.1231415357,Spirochaeta lutea
@Spirochaeta_Spirochaetaceae_Spirochaetales_Spirochaetes_Bacteria
WP_081942209:0.2824452818)100:0.0465996520)100:0.0674143224)67:0.0232939474,(((Alcanivorax dieselolei
@Alcanivorax_Alcanivoracaceae_Oceanospirillales_Gammaproteobacteria_Proteobacteria_Bacteria
WP_014996582:0.2100889727,((Dyella japonica
@Dyella_Rhodanobacteraceae_Xanthomonadales_Gammaproteobacteria_Proteobacteria_Bacteria
WP_046971866:0.1592866557,Rhodanobacter denitrificans
@Rhodanobacter_Rhodanobacteraceae_Xanthomonadales_Gammaproteobacteria_Proteobacteria_Bacteria
KZC21690:0.1298918186)100:0.0633115706,(Frateuria aurantia
@Frateuria_Rhodanobacteraceae_Xanthomonadales_Gammaproteobacteria_Proteobacteria_Bacteria
WP_014402021:0.1726691660,Kerstersia gyiorum
@Kerstersia_Alcaligenaceae_Burkholderiales_Betaproteobacteria_Proteobacteria_Bacteria
WP_068372127:0.1955028720)89:0.0537732315)89:0.0406607652)100:0.0722590092,Salpingoeca rosetta
@Salpingoeca_Salpingoecidae_Craspedida_Choanoflagellida_Eukaryota
XP_004990707:0.6233918526)82:0.0202512037,(Bradyrhizobium ottawaense

@Bradyrhizobium_Bradyrhizobiaceae_Rhizobiales_Alphaproteobacteria_Proteobacteria_Bacteria
SDJ18903:0.1768650508,Lutibaculum baratangense
@Lutibaculum_Rhodobiaceae_Rhizobiales_Alphaproteobacteria_Proteobacteria_Bacteria
WP_023434261:0.2442457939)100:0.1097729051)78:0.0365718830)68:0.0355993567,(Tepidicaulis marinus
@Rhodobiaceae_Rhizobiales_Alphaproteobacteria_Proteobacteria_Bacteria WP_045442100:0.3256447440,Tistrella
mobilis @Tistrella_Rhodospirillaceae_Rhodospirillales_Alphaproteobacteria_Proteobacteria_Bacteria
WP_014743774:0.4041658958)93:0.0489399938)78:0.0397443947,((((((((((((((((((((((((Blastocrithidia sp. p57
@Blastocrithidia_Trypanosomatidae_Kinetoplastida_Euglenozoa_Eukaryota p57:0.0000028988,Blastocrithidia
triatomae @Blastocrithidia_Trypanosomatidae_Kinetoplastida_Euglenozoa_Eukaryota
triat:0.0040932462)100:0.2238484945,jaculum @Jaculum_Trypanosomatidae_Kinetoplastida_Euglenozoa_Eukaryota
jac:0.3122261238)100:0.0778034010,(Taylorella asinigenitalis
@Taylorella_Alcaligenaceae_Burkholderiales_Betaproteobacteria_Proteobacteria_Bacteria
WP_014111929:0.0250347262,Taylorella equigenitalis
@Taylorella_Alcaligenaceae_Burkholderiales_Betaproteobacteria_Proteobacteria_Bacteria
WP_013521500:0.0318887682)100:0.1708793003)100:0.2338595704,(Capnocytophaga stomatis
@Capnocytophaga_Flavobacteriaceae_Flavobacteriales_Flavobacteriia_Bacteroidetes_Bacteria
WP_095894878:0.1891134948,Sulfurimonas hongkongensis
@Sulfurimonas_Helicobacteraceae_Campylobacterales_Epsilonproteobacteria_Proteobacteria_Bacteria
WP_021286763:0.1566576725)96:0.0143172098)55:0.0165889341,((((Aequorivita vladivostokensis
@Aequorivita_Flavobacteriaceae_Flavobacteriales_Flavobacteriia_Bacteroidetes_Bacteria
WP_045080179:0.0039741798,Vitellibacter aquimaris
@Aequorivita_Flavobacteriaceae_Flavobacteriales_Flavobacteriia_Bacteroidetes_Bacteria
WP_062619759:0.0000029503)100:0.0450666232,(Arenibacter certesii
@Arenibacter_Flavobacteriaceae_Flavobacteriales_Flavobacteriia_Bacteroidetes_Bacteria
WP_026815260:0.0444340687,((Bizionia argentinensis
@Bizionia_Flavobacteriaceae_Flavobacteriales_Flavobacteriia_Bacteroidetes_Bacteria
WP_008639015:0.0291914642,Xanthomarina gelatinilytica
@Xanthomarina_Flavobacteriaceae_Flavobacteriales_Flavobacteriia_Bacteroidetes_Bacteria
WP_007649750:0.0541186531)100:0.0297716218,Gelidibacter mesophilus
@Gelidibacter_Flavobacteriaceae_Flavobacteriales_Flavobacteriia_Bacteroidetes_Bacteria
WP_027126534:0.0447644404)100:0.0225184370)99:0.0215101354)99:0.0506582522,Chryseobacterium antarcticum
@Chryseobacterium_Flavobacteriaceae_Flavobacteriales_Flavobacteriia_Bacteroidetes_Bacteria
WP_034719812:0.1131102570)100:0.1116262666,Flavobacterium chilense
@Flavobacterium_Flavobacteriaceae_Flavobacteriales_Flavobacteriia_Bacteroidetes_Bacteria
WP_068843279:0.1786308328)99:0.0850431304)76:0.0240625632,((((((((((Aliicoccus persicus
@Aliicoccus_Staphylococcaceae_Bacillales_Bacilli_Firmicutes_Bacteria WP_091475516:0.0497224735,Jeotgalicoccus
halophilus @Jeotgalicoccus_Staphylococcaceae_Bacillales_Bacilli_Firmicutes_Bacteria
WP_092599508:0.0459142468)100:0.0212121395,Nosocomiicoccus massiliensis
@Nosocomiicoccus_Staphylococcaceae_Bacillales_Bacilli_Firmicutes_Bacteria
WP_102167606:0.1391986624)99:0.0270181566,Salinicoccus kekensis
@Salinicoccus_Staphylococcaceae_Bacillales_Bacilli_Firmicutes_Bacteria
SOC42587:0.0722364716)100:0.0650543874,(Macrococcus canis
@Macrococcus_Staphylococcaceae_Bacillales_Bacilli_Firmicutes_Bacteria
WP_086041522:0.0733340775,(Mycobacterium abscessus @Mycobacteroides
abscessus_Mycobacteriaceae_Corynebacteriales_Actinobacteria_Bacteria SKR87466:0.0655106771,Staphylococcus
aureus @Staphylococcus_Staphylococcaceae_Bacillales_Bacilli_Firmicutes_Bacteria
WP_031870206:0.0223762354)100:0.1176482749)100:0.0560614891)63:0.0261561834,Auricoccus indicus
@Bacillales_Bacilli_Firmicutes_Bacteria WP_077141107:0.1814075237)100:0.1335929611,((((Bacillus cecembensis
@Bacillus_Bacillaceae_Bacillales_Bacilli_Firmicutes_Bacteria WP_057988386:0.0588964964,Kurthia huakuii

@Kurthia_Planococcaceae_Bacillales_Bacilli_Firmicutes_Bacteria
WP_029497661:0.0717010704)100:0.0231807256,(Caryophanon tenue
@Caryophanon_Planococcaceae_Bacillales_Bacilli_Firmicutes_Bacteria WP_066546585:0.0705148005,Solibacillus
silvestris @Solibacillus_Planococcaceae_Bacillales_Bacilli_Firmicutes_Bacteria
WP_014824979:0.0000021535)100:0.0280276381)100:0.1247344940,((Bacillus ndiopicus
@Bacillus_Bacillaceae_Bacillales_Bacilli_Firmicutes_Bacteria WP_042470300:0.1032039410,Lysinibacillus contaminans
@Lysinibacillus_Bacillaceae_Bacillales_Bacilli_Firmicutes_Bacteria
WP_053585603:0.0531496339)59:0.0095521803,Ureibacillus thermosphaericus
@Ureibacillus_Planococcaceae_Bacillales_Bacilli_Firmicutes_Bacteria
WP_016837596:0.1666978400)100:0.0222074143)95:0.0208371435,Rummeliibacillus pycnus
@Rummeliibacillus_Planococcaceae_Bacillales_Bacilli_Firmicutes_Bacteria
WP_102693306:0.1378232970)87:0.0562264795)89:0.0255124715,(((Bacillus coagulans
@Bacillus_Bacillaceae_Bacillales_Bacilli_Firmicutes_Bacteria KYC65640:0.0542178945,Tuberibacillus calidus
@Tuberibacillus_Sporolactobacillaceae_Bacillales_Bacilli_Firmicutes_Bacteria
WP_027724119:0.0446898284)100:0.0397601163,Oceanobacillus caeni @Bacillales_Bacilli_Firmicutes_Bacteria
WP_047183650:0.1120592425)92:0.0230132842,(Ornithinibacillus scapharcae
@Ornithinibacillus_Bacillaceae_Bacillales_Bacilli_Firmicutes_Bacteria WP_010098093:0.0342634360,Paucisalibacillus
globulus @Paucisalibacillus_Bacillaceae_Bacillales_Bacilli_Firmicutes_Bacteria
WP_096270964:0.0663081481)100:0.0493635513)100:0.1048910366)91:0.0263141674,(Bacillus massilioanorexius
@Bacillus_Bacillaceae_Bacillales_Bacilli_Firmicutes_Bacteria WP_019240222:0.0765093605,Veillonella dispar
@Veillonella_Veillonellaceae_Veillonellales_Negativicutes_Firmicutes_Bacteria
WP_005385023:0.3224277245)97:0.0666996282)32:0.0048052985,Natribacillus halophilus
@Natribacillus_Bacillaceae_Bacillales_Bacilli_Firmicutes_Bacteria
SDI81044:0.2046119084)79:0.0370044326,(((Lysinibacillus sphaericus
@Lysinibacillus_Bacillaceae_Bacillales_Bacilli_Firmicutes_Bacteria WP_075527803:0.1452537207,Psychrobacillus
psychrotolerans @Psychrobacillus_Bacillaceae_Bacillales_Bacilli_Firmicutes_Bacteria
WP_093534569:0.1256578936)70:0.0195290734,Virgibacillus halodenitrificans
@Virgibacillus_Bacillaceae_Bacillales_Bacilli_Firmicutes_Bacteria
WP_060681902:0.0426012043)100:0.0287452146,Sporosarcina ureae
@Sporosarcina_Planococcaceae_Bacillales_Bacilli_Firmicutes_Bacteria
WP_037561528:0.1404570178)100:0.0355584923)81:0.0208512552,(((((Fermentimonas caenicola
@Fermentimonas_Porphyromonadaceae_Bacteroidales_Bacteroidia_Bacteroidetes_Bacteria
WP_045088851:0.0000023247,Lascolabacillus massiliensis
@Lascolabacillus_Porphyromonadaceae_Bacteroidales_Bacteroidia_Bacteroidetes_Bacteria
WP_053826857:0.0000028226)100:0.0257559679,Proteiniphilum acetatigenes
@Proteiniphilum_Dysgonamonadaceae_Bacteroidales_Bacteroidia_Bacteroidetes_Bacteria
WP_019538794:0.0495137978)100:0.0236433782,Petrimonas mucosa
@Petrimonas_Porphyromonadaceae_Bacteroidales_Bacteroidia_Bacteroidetes_Bacteria
WP_071137235:0.0183553953)100:0.0462853092,Ignatzschineria larvae
@Ignatzschineria_Gammaproteobacteria_Proteobacteria_Bacteria
WP_026879052:0.1925459453)100:0.0264707717,Sphaerochaeta globosa
@Sphaerochaeta_Spirochaetaceae_Spirochaetales_Spirochaetes_Bacteria
WP_013607196:0.3518358754)95:0.0363971268)43:0.0194027437)44:0.0133175733,((((((((Actinomyces oris
@Actinomyces_Actinomycetaceae_Actinomycetales_Actinobacteria_Bacteria
WP_065362056:0.1888646444,Luteococcus japonicus
@Luteococcus_Propionibacteriaceae_Propionibacteriales_Actinobacteria_Bacteria
WP_094765957:0.1790738189)100:0.1377253790,(((Anaerococcus prevotii
@Anaerococcus_Peptoniphilaceae_Tissierellales_Tissierellia_Firmicutes_Bacteria
WP_015777503:0.0581699185,Peptoniphilus timonensis

@Peptoniphilus_Peptoniphilaceae_Tissierellales_Tissierellia_Firmicutes_Bacteria
WP_019138835:0.0774951900)100:0.1890738436,Ignavigranum ruoffiae
@Ignavigranum_Aerococcaceae_Lactobacillales_Bacilli_Firmicutes_Bacteria
WP_092571913:0.1873574510)98:0.0521424552,Porphyromonas bennonis
@Porphyromonas_Porphyromonadaceae_Bacteroidales_Bacteroidia_Bacteroidetes_Bacteria
WP_018107270:0.2444562847)93:0.0468876446)67:0.0305251558,Finegoldia magna
@Finegoldia_Peptoniphilaceae_Tissierellales_Tissierellia_Firmicutes_Bacteria
WP_094206890:0.2234343980)74:0.0480511215,(((Anaerococcus vaginalis
@Anaerococcus_Peptoniphilaceae_Tissierellales_Tissierellia_Firmicutes_Bacteria
WP_004839131:0.0432226427,Neofamilia massiliensis @Neofamilia_Clostridiales_Clostridia_Firmicutes_Bacteria
WP_054253000:0.0429381743)72:0.0134681656,Lagierella massiliensis
@Peptoniphilaceae_Tissierellales_Tissierellia_Firmicutes_Bacteria
WP_055078472:0.0194247783)71:0.0130180417,Ezakiella peruensis @Ezakiella_Tissierellia_Firmicutes_Bacteria
WP_099950882:0.0694442759)100:0.2429533798)73:0.0185793036,((Aerococcus suis
@Aerococcus_Aerococcaceae_Lactobacillales_Bacilli_Firmicutes_Bacteria
WP_084099345:0.1696847992,Tetragenococcus muriaticus
@Tetragenococcus_Enterococcaceae_Lactobacillales_Bacilli_Firmicutes_Bacteria
WP_028789464:0.0764336662)100:0.0830307568,Alloiococcus otitis
@Alloiococcus_Carnobacteriaceae_Lactobacillales_Bacilli_Firmicutes_Bacteria
WP_003778169:0.1873921111)95:0.0899730660)62:0.0155607089,Dehalobacterium formicoaceticum
@Dehalobacterium_Peptococcaceae_Clostridiales_Clostridia_Firmicutes_Bacteria
WP_089609705:0.1451609584)94:0.0381900632,Streptococcus didelphis
@Streptococcus_Streptococcaceae_Lactobacillales_Bacilli_Firmicutes_Bacteria
WP_018366408:0.1872860825)99:0.0296960912,(Methanosarcina flavescens
@Methanosarcina_Methanosarcinaceae_Methanosarcinales_Methanomicrobia_Euryarchaeota_Archaea
WP_054298813:0.0825639797,(Proteocatella sphenisci
@Proteocatella_Peptostreptococcaceae_Clostridiales_Clostridia_Firmicutes_Bacteria
WP_051188457:0.0526408873,Tissierella creatinophila
@Tissierella_Tissierellaceae_Tissierellales_Tissierellia_Firmicutes_Bacteria
WP_075726643:0.1134675803)100:0.0790421079)100:0.0435043558)84:0.0324943108,Peptococcus niger
@Peptococcus_Peptococcaceae_Clostridiales_Clostridia_Firmicutes_Bacteria
WP_091791229:0.2110739892)100:0.0901339581)95:0.0589399317,Mesotoga prima
@Mesotoga_Kosmotogaceae_Kosmotogales_Thermotogae_Bacteria
WP_014729999:0.1032517841)80:0.0130116113,Brevibacillus panacihumi
@Brevibacillus_Paenibacillaceae_Bacillales_Bacilli_Firmicutes_Bacteria
WP_023558005:0.1369335102)81:0.0071833380,(((Desulfitobacterium dichloroeliminans
@Desulfitobacterium_Peptococcaceae_Clostridiales_Clostridia_Firmicutes_Bacteria
WP_015261510:0.0735559860,Methanomethylovorans hollandica
@Methanomethylovorans_Methanosarcinaceae_Methanosarcinales_Methanomicrobia_Euryarchaeota_Archaea
WP_015325093:0.0906532156)79:0.0151135135,Miniphocibacter massiliensis
@Miniphocibacter_Peptoniphilaceae_Tissierellales_Tissierellia_Firmicutes_Bacteria
WP_100066157:0.3610929681)53:0.0196180261,(Desulfotomaculum aeronauticum
@Desulfotomaculum_Peptococcaceae_Clostridiales_Clostridia_Firmicutes_Bacteria
WP_072914854:0.0497930409,Succinispira mobilis
@Succinispira_Acidaminococcaceae_Acidaminococcales_Negativicutes_Firmicutes_Bacteria
WP_019879827:0.1353894537)79:0.0091346530)66:0.0129339511)66:0.0143134398,((Brevefilum fermentans
@Brevefilum_Anaerolineaceae_Anaerolineales_Anaerolineae_Chloroflexi_Bacteria
WP_087861573:0.1085535000,Levilinea saccharolytica
@Levilinea_Anaerolineaceae_Anaerolineales_Anaerolineae_Chloroflexi_Bacteria

WP_062419119:0.0564209093)100:0.0283675863,Methanofollis liminatans
@Methanofollis_Methanomicrobiaceae_Methanomicrobiales_Methanomicrobia_Euryarchaeota_Archaea
WP_004039435:0.0988908401)100:0.0647177833)97:0.0233600023,(((((Novymonas esmeraldas
@Novymonas_Trypanosomatidae_Kinetoplastida_Euglenozoa_Eukaryota ANJ89233:0.0582480944,(((Leptomonas
pyrrhocoris @Leptomonas_Leishmaniinae_Trypanosomatidae_Kinetoplastida_Euglenozoa_Eukaryota
XP_015656182:0.0378107848,Crithidia bombi
@Crithidia_Leishmaniinae_Trypanosomatidae_Kinetoplastida_Euglenozoa_Eukaryota
cbom:0.0879193281)95:0.0142676505,Leptomonas seymouri
@Leptomonas_Leishmaniinae_Trypanosomatidae_Kinetoplastida_Euglenozoa_Eukaryota
KPI89353:0.0885137957)92:0.0197396085,((((Lotmaria passim
@Lotmaria_Leishmaniinae_Trypanosomatidae_Kinetoplastida_Euglenozoa_Eukaryota lpas:0.0553465761,(Crithidia
brevicula @Crithidia_Leishmaniinae_Trypanosomatidae_Kinetoplastida_Euglenozoa_Eukaryota
ANJ89235:0.0333763363,Crithidia thermophila
@Crithidia_Leishmaniinae_Trypanosomatidae_Kinetoplastida_Euglenozoa_Eukaryota
ANJ89234:0.0664633266)100:0.0317160545)100:0.0271287706,Crithidia mellificae
@Crithidia_Leishmaniinae_Trypanosomatidae_Kinetoplastida_Euglenozoa_Eukaryota
cmel:0.0207400574)93:0.0103341547,Crithidia fasciculata
@Crithidia_Leishmaniinae_Trypanosomatidae_Kinetoplastida_Euglenozoa_Eukaryota
cfas:0.0270345862)89:0.0112766145,Crithidia acanthocephali
@Crithidia_Leishmaniinae_Trypanosomatidae_Kinetoplastida_Euglenozoa_Eukaryota
caca:0.0000024489)96:0.0297215541)100:0.0969569255)100:0.0892873903,((((Brachyspira alvinipulli
@Brachyspira_Brachyspiraceae_Brachyspirales_Spirochaetes_Bacteria WP_028329494:0.0245952951,Brachyspira
murdochii @Brachyspira_Brachyspiraceae_Brachyspirales_Spirochaetes_Bacteria
WP_104619231:0.0088163209)100:0.0166880855,Brachyspira innocens
@Brachyspira_Brachyspiraceae_Brachyspirales_Spirochaetes_Bacteria
WP_020003575:0.0048580455)100:0.0226507990,(Brachyspira intermedia
@Brachyspira_Brachyspiraceae_Brachyspirales_Spirochaetes_Bacteria AEM21566:0.0058558510,Brachyspira pilosicoli
@Brachyspira_Brachyspiraceae_Brachyspirales_Spirochaetes_Bacteria
WP_013243239:0.0108092900)100:0.0084074423)99:0.0044311768,Brachyspira hampsonii
@Brachyspira_Brachyspiraceae_Brachyspirales_Spirochaetes_Bacteria
WP_008726147:0.0058844101)100:0.1639591067)100:0.1283309581,(((Bacteroides barnesiae
@Bacteroides_Bacteroidaceae_Bacteroidales_Bacteroidia_Bacteroidetes_Bacteria
WP_025892754:0.0310153121,Mediterranea massiliensis
@Mediterranea_Bacteroidaceae_Bacteroidales_Bacteroidia_Bacteroidetes_Bacteria
WP_072546537:0.0276883909)100:0.0684985759,Porphyromonas cangingivalis
@Porphyromonas_Porphyromonadaceae_Bacteroidales_Bacteroidia_Bacteroidetes_Bacteria
WP_036850591:0.0609312032)100:0.0521346478,(Chromobacterium vaccinii
@Chromobacterium_Chromobacteriaceae_Neisseriales_Betaproteobacteria_Proteobacteria_Bacteria
WP_046166820:0.0070125286,Pseudogulbenkiania ferrooxidans
@Pseudogulbenkiania_Chromobacteriaceae_Neisseriales_Betaproteobacteria_Proteobacteria_Bacteria
WP_021478981:0.0100361174)100:0.1415099621)94:0.0193643755)74:0.0298723847,Akkermansia muciniphila
@Akkermansia_Akkermansiaceae_Verrucomicrobiales_Verrucomicrobiae_Verrucomicrobia_Bacteria
WP_102712127:0.1751904834)26:0.0098258769,((((((Alistipes obesi
@Rikenellaceae_Bacteroidales_Bacteroidia_Bacteroidetes_Bacteria WP_019129622:0.1530478834,((Coprobacter
secundus @Coprobacter_Barnesiellaceae_Bacteroidales_Bacteroidia_Bacteroidetes_Bacteria
WP_044229047:0.0000028009,Gabonia massiliensis
@Gabonia_Porphyromonadaceae_Bacteroidales_Bacteroidia_Bacteroidetes_Bacteria
WP_055100169:0.0040461564)100:0.1199006360,(Gabonibacter massiliensis
@Gabonibacter_Porphyromonadaceae_Bacteroidales_Bacteroidia_Bacteroidetes_Bacteria

WP_059026437:0.0040711272,Sanguibacteroides justesenii
@Sanguibacteroides_Porphyromonadaceae_Bacteroidales_Bacteroidia_Bacteroidetes_Bacteria
WP_041504213:0.0000029332)100:0.0916053116)100:0.0647932103)100:0.0584378031,Bacteroides fragilis
@Bacteroides_Bacteroidaceae_Bacteroidales_Bacteroidia_Bacteroidetes_Bacteria
WP_032543326:0.0504711844)96:0.0385563829,Parabacteroides distasonis
@Parabacteroides_Tannerellaceae_Bacteroidales_Bacteroidia_Bacteroidetes_Bacteria
WP_057328702:0.0780843792)100:0.0486343854,(Faecalibacterium prausnitzii
@Faecalibacterium_Ruminococcaceae_Clostridiales_Clostridia_Firmicutes_Bacteria
WP_055205053:0.1407572687,(Millionella massiliensis
@Millionella_Rikenellaceae_Bacteroidales_Bacteroidia_Bacteroidetes_Bacteria WP_071133620:0.0264252289,Rikenella microfusus @Rikenella_Rikenellaceae_Bacteroidales_Bacteroidia_Bacteroidetes_Bacteria
WP_037291894:0.0662140890)100:0.0821638153)100:0.0321927420)100:0.0671598840,(Desulfuromonas acetexigens
@Desulfuromonas_Desulfuromonadaceae_Desulfuromonadales_Deltaproteobacteria_Proteobacteria_Bacteria
WP_092054230:0.0610222887,Pelobacter acetylenicus
@Pelobacter_Desulfuromonadaceae_Desulfuromonadales_Deltaproteobacteria_Proteobacteria_Bacteria
WP_072285874:0.1032701455)100:0.1031280705)98:0.0317292030,(((((((((((Anaerocolumna xylanovorans
@Anaerocolumna_Lachnospiraceae_Clostridiales_Clostridia_Firmicutes_Bacteria
WP_073587377:0.0499408195,(Cellulosilyticum lentocellum
@Cellulosilyticum_Lachnospiraceae_Clostridiales_Clostridia_Firmicutes_Bacteria
WP_013655203:0.0132188903,Lachnoclostridium phytofermentans
@Lachnospiraceae_Clostridiales_Clostridia_Firmicutes_Bacteria
WP_029505074:0.0327077123)95:0.0320226931)87:0.0327833505,((Anaerosphaera aminiphila
@Anaerosphaera_Peptoniphilaceae_Tissierellales_Tissierellia_Firmicutes_Bacteria
WP_073183221:0.1134603818,((Catabacter hongkongensis
@Catabacter_Catabacteriaceae_Clostridiales_Clostridia_Firmicutes_Bacteria
WP_046443954:0.0624594923,Robinsoniella peoriensis
@Robinsoniella_Lachnospiraceae_Clostridiales_Clostridia_Firmicutes_Bacteria
WP_047832716:0.0735570771)96:0.0365053220,(Clostridium termitidis
@Ruminiclostridium_Hungateiclostridiaceae_Clostridiales_Clostridia_Firmicutes_Bacteria
WP_004624845:0.0711250052,Sphaerochaeta coccoides
@Sphaerochaeta_Spirochaetaceae_Spirochaetales_Spirochaetes_Bacteria
WP_013738892:0.0742088349)72:0.0000023554)94:0.0215103094)86:0.0369416267,Treponema brennaborense
@Treponema_Spirochaetaceae_Spirochaetales_Spirochaetes_Bacteria
WP_013757552:0.0605935407)100:0.0500758940)100:0.0598305179,Blautia hominis
@Blautia_Lachnospiraceae_Clostridiales_Clostridia_Firmicutes_Bacteria
WP_095174123:0.0684137447)91:0.0345485395,Bittarella massiliensis
@Bittarella_Ruminococcaceae_Clostridiales_Clostridia_Firmicutes_Bacteria
WP_059004798:0.0917002776)97:0.0302452492,((Eisenbergiella massiliensis
@Eisenbergiella_Lachnospiraceae_Clostridiales_Clostridia_Firmicutes_Bacteria WP_102288670:0.0454387430,Neglecta timonensis @Neglecta_Ruminococcaceae_Clostridiales_Clostridia_Firmicutes_Bacteria
WP_066864481:0.0273940205)100:0.0227510028,Hungatella hathewayi
@Hungatella_Clostridiaceae_Clostridiales_Clostridia_Firmicutes_Bacteria
WP_055652667:0.1408260547)97:0.0174589979)99:0.0519957534,(Anaeromassilibacillus senegalensis
@Anaeromassilibacillus_Ruminococcaceae_Clostridiales_Clostridia_Firmicutes_Bacteria
WP_050699064:0.0531842157,Bariatricus massiliensis
@Bariatricus_Lachnospiraceae_Clostridiales_Clostridia_Firmicutes_Bacteria
WP_066733821:0.0974098959)83:0.0000027895)99:0.0456685735,Methanomassiliicoccus intestinalis
@Methanomassiliicoccus_Methanomassiliicoccaceae_Methanomassiliicoccales_Thermoplasmata_Euryarchaeota_Archaea WP_102030401:0.0595347747)100:0.1209332811,(Christensenella timonensis

@Christensenella_Christensenellaceae_Clostridiales_Clostridia_Firmicutes_Bacteria
WP_066649157:0.1303966906,Pygmaiobacter massiliensis
@Pygmaiobacter_Ruminococcaceae_Clostridiales_Clostridia_Firmicutes_Bacteria
WP_102051103:0.3006047994)99:0.0159777718)100:0.1013731024,Anaerorhabdus furcosa
@Anaerorhabdus_Bacteroidaceae_Bacteroidales_Bacteroidia_Bacteroidetes_Bacteria
WP_078712294:0.2375950392)100:0.0700766100,Methanocorpusculum parvum
@Methanocorpusculum_Methanocorpusculaceae_Methanomicrobiales_Methanomicrobia_Euryarchaeota_Archaea
WP_095642576:0.1679164003)84:0.0358579694,((((Arabia massiliensis
@Arabia_Eggerthellaceae_Eggerthellales_Coriobacteriia_Actinobacteria_Bacteria
WP_080800673:0.0287778645,Gordonibacter massiliensis
@Gordonibacter_Eggerthellaceae_Eggerthellales_Coriobacteriia_Actinobacteria_Bacteria
WP_080144252:0.0353819253)85:0.0257405238,Clostridioides difficile
@Clostridioides_Peptostreptococcaceae_Clostridiales_Clostridia_Firmicutes_Bacteria
EQI16178:0.0220557788)48:0.0117882714,Eggerthella timonensis
@Eggerthella_Eggerthellaceae_Eggerthellales_Coriobacteriia_Actinobacteria_Bacteria
WP_101721206:0.0267580178)61:0.0378441084,Raoultibacter massiliensis
@Raoultibacter_Eggerthellaceae_Eggerthellales_Coriobacteriia_Actinobacteria_Bacteria
WP_102375673:0.0902431237)100:0.2269796465)74:0.0431248077,Methanomethylophilus alvus @Candidatus
Methanomethylophilus_Methanomassiliicoccaceae_Methanomassiliicoccales_Thermoplasmata_Euryarchaeota_Archae
a WP_048097703:0.2183142471)100:0.1683990615)26:0.0094722523)90:0.0189718260)97:0.0297587789,Chrysiogenes
arsenatis @Chrysiogenes_Chrysiogenaceae_Chrysiogenales_Chrysiogenetes_Bacteria
WP_027389929:0.0908786495)86:0.0167453002,Methanothrix soehngenii
@Methanothrix_Methanotrichaceae_Methanosarcinales_Methanomicrobia_Stenosarchaea
group_Euryarchaeota_Archaea WP_013718126:0.1417812823)94:0.0118257070,(((Brocadia sinica @Candidatus
Brocadia_Candidatus Brocadiaceae_Candidatus Brocadiales_Planctomycetia_Planctomycetes_Bacteria
KXK25293:0.1591455729,(Desulfofaba hansenii
@Desulfofaba_Desulfobacteraceae_Desulfobacterales_Deltaproteobacteria_Proteobacteria_Bacteria
WP_100390614:0.0748697274,Desulfovibrio frigidus
@Desulfovibrio_Desulfovibrionaceae_Desulfovibrionales_Deltaproteobacteria_Proteobacteria_Bacteria
WP_031485507:0.1345249352)100:0.0315876121)100:0.0093179534,(Desulfobacula phenolica
@Desulfobacula_Desulfobacteraceae_Desulfobacterales_Deltaproteobacteria_Proteobacteria_Bacteria
WP_092236874:0.1169165506,((Desulforegula conservatrix
@Desulforegula_Desulfobacteraceae_Desulfobacterales_Deltaproteobacteria_Proteobacteria_Bacteria
WP_027358937:0.0771083760,Geobacter anodireducens
@Geobacter_Geobacteraceae_Desulfuromonadales_Deltaproteobacteria_Proteobacteria_Bacteria
WP_066357887:0.1347745299)94:0.0255804397,(Desulfotalea psychrophila
@Desulfotalea_Desulfobulbaceae_Desulfobacterales_Deltaproteobacteria_Proteobacteria_Bacteria
WP_011190244:0.1712737130,Scalindua brodae @Candidatus Scalindua_Candidatus Brocadiaceae_Candidatus
Brocadiales_Planctomycetia_Planctomycetes_Bacteria
KHE93022:0.1331331398)97:0.0716252481)86:0.0156030603)81:0.0318845223)76:0.0169489042,(Pelobacter
propionicus @Pelobacter_Desulfuromonadaceae_Desulfuromonadales_Deltaproteobacteria_Proteobacteria_Bacteria
WP_011735807:0.0838350540,Syntrophobacter fumaroxidans
@Syntrophobacter_Syntrophobacteraceae_Syntrophobacterales_Deltaproteobacteria_Proteobacteria_Bacteria
WP_011698578:0.0653045150)100:0.0659612465)81:0.0114912278)85:0.0208548563,((Bilophila wadsworthia
@Bilophila_Desulfovibrionaceae_Desulfovibrionales_Deltaproteobacteria_Proteobacteria_Bacteria
WP_029436121:0.1008569506,Desulfovibrio aminophilus
@Desulfovibrio_Desulfovibrionaceae_Desulfovibrionales_Deltaproteobacteria_Proteobacteria_Bacteria
WP_027177109:0.1265318920)85:0.0501628979,(((Desulfobacter postgatei
@Desulfobacter_Desulfobacteraceae_Desulfobacterales_Deltaproteobacteria_Proteobacteria_Bacteria

WP_004071151:0.1197526589,Syntrophus aciditrophicus
@Syntrophus_Syntrophaceae_Syntrophobacterales_Deltaproteobacteria_Proteobacteria_Bacteria
WP_041585147:0.0572652042)98:0.0573152891,(Desulfobulbus elongatus
@Desulfobulbus_Desulfobulbaceae_Desulfobacterales_Deltaproteobacteria_Proteobacteria_Bacteria
WP_028317960:0.0871289918,Desulfomicrobium norvegicum
@Desulfomicrobium_Desulfomicrobiaceae_Desulfovibrionales_Deltaproteobacteria_Proteobacteria_Bacteria
WP_092194216:0.0283657498)98:0.0310470850)100:0.0197958532,Syntrophorhabdus aromaticivorans
@Syntrophorhabdus_Syntrophorhabdaceae_Syntrophobacterales_Deltaproteobacteria_Proteobacteria_Bacteria
WP_028895679:0.1414318537)54:0.0114210969)80:0.0141707612)89:0.0415026917,((((Achromobacter denitrificans
@Achromobacter_Alcaligenaceae_Burkholderiales_Betaproteobacteria_Proteobacteria_Bacteria
WP_088447726:0.2399774924,((((((((Acinetobacter albensis
@Acinetobacter_Moraxellaceae_Pseudomonadales_Gammaproteobacteria_Proteobacteria_Bacteria
WP_092718430:0.1463077446,(((Acinetobacter populi
@Acinetobacter_Moraxellaceae_Pseudomonadales_Gammaproteobacteria_Proteobacteria_Bacteria
WP_087619460:0.0679780296,Gallibacterium anatis
@Gallibacterium_Pasteurellaceae_Pasteurellales_Gammaproteobacteria_Proteobacteria_Bacteria
WP_039150242:0.0450682935)36:0.0183137508,Avibacterium gallinarum
@Avibacterium_Pasteurellaceae_Pasteurellales_Gammaproteobacteria_Proteobacteria_Bacteria
WP_103853672:0.0327509804)38:0.0167790680,(Chelonobacter oris
@Chelonobacter_Pasteurellaceae_Pasteurellales_Gammaproteobacteria_Proteobacteria_Bacteria
WP_034616271:0.0057264712,Pasteurella testudinis
@Pasteurella_Pasteurellaceae_Pasteurellales_Gammaproteobacteria_Proteobacteria_Bacteria
WP_084256723:0.0197880381)100:0.0321501365)82:0.0187414742)59:0.0031821477,(((((Idiomarina indica
@Idiomarina_Idiomarinaceae_Alteromonadales_Gammaproteobacteria_Proteobacteria_Bacteria
WP_092592176:0.0956982226,Pseudoalteromonas haloplanktis
@Pseudoalteromonas_Pseudoalteromonadaceae_Alteromonadales_Gammaproteobacteria_Proteobacteria_Bacteria
WP_011328413:0.0695753150)100:0.0366531684,Psychrobacter urativorans
@Psychrobacter_Moraxellaceae_Pseudomonadales_Gammaproteobacteria_Proteobacteria_Bacteria
WP_062535327:0.0925148126)99:0.0310203509,Klebsiella quasipneumoniae
@Klebsiella_Enterobacteriaceae_Enterobacterales_Gammaproteobacteria_Proteobacteria_Bacteria
WP_101862728:0.1267731366)70:0.0156596476,Moraxella boevrei
@Moraxella_Moraxellaceae_Pseudomonadales_Gammaproteobacteria_Proteobacteria_Bacteria
WP_019519938:0.0956623562)58:0.0147608165,(Paenalcaligenes hominis
@Paenalcaligenes_Alcaligenaceae_Burkholderiales_Betaproteobacteria_Proteobacteria_Bacteria
WP_077733848:0.2134404487,(Snodgrassella alvi
@Snodgrassella_Neisseriaceae_Neisseriales_Betaproteobacteria_Proteobacteria_Bacteria
WP_100100401:0.2052617691,Suttonella ornithocola
@Suttonella_Cardiobacteriaceae_Cardiobacteriales_Gammaproteobacteria_Proteobacteria_Bacteria
WP_072577180:0.0767211595)57:0.0109309709)57:0.0076053670)83:0.0212647910)44:0.0045524694,((((Actinobacillu
s capsulatus @Actinobacillus_Pasteurellaceae_Pasteurellales_Gammaproteobacteria_Proteobacteria_Bacteria
WP_018652786:0.0214742112,Simonsiella muelleri
@Simonsiella_Neisseriaceae_Neisseriales_Betaproteobacteria_Proteobacteria_Bacteria
WP_002641686:0.0355852339)100:0.0717719673,((Conchiformibius kuhniae
@Conchiformibius_Neisseriaceae_Neisseriales_Betaproteobacteria_Proteobacteria_Bacteria
WP_027009529:0.0464356570,Mannheimia varigena
@Mannheimia_Pasteurellaceae_Pasteurellales_Gammaproteobacteria_Proteobacteria_Bacteria
WP_025248258:0.0401503967)90:0.0211313211,Neisseria weaveri
@Neisseriaceae_Neisseriales_Betaproteobacteria_Proteobacteria_Bacteria
WP_036494282:0.0379176937)34:0.0093934219)91:0.0318054598,Moraxella caviae

@Moraxella_Moraxellaceae_Pseudomonadales_Gammaproteobacteria_Proteobacteria_Bacteria
WP_078276912:0.0646690874)43:0.0049880800,(((Aggregatibacter segnis
@Aggregatibacter_Pasteurellaceae_Pasteurellales_Gammaproteobacteria_Proteobacteria_Bacteria
WP_048750208:0.0175729757,Haemophilus influenzae
@Haemophilus_Pasteurellaceae_Pasteurellales_Gammaproteobacteria_Proteobacteria_Bacteria
AAA20441:0.0238222884)100:0.0198915440,((Morococcus cerebrosus
@Neisseriales_Betaproteobacteria_Proteobacteria_Bacteria WP_049225874:0.0000020787,Porphyrobacter
donghaensis @Neisseria_Neisseriaceae_Neisseriales_Betaproteobacteria_Proteobacteria_Bacteria
WP_060975515:0.0085904331)100:0.0273290529,Rodentibacter ratti
@Rodentibacter_Pasteurellaceae_Pasteurellales_Gammaproteobacteria_Proteobacteria_Bacteria
WP_077435008:0.0266385245)62:0.0130604495)98:0.0242656212,(Bergeriella denitrificans
@Bergeriella_Neisseriaceae_Neisseriales_Betaproteobacteria_Proteobacteria_Bacteria
WP_066077770:0.0350567666,Moraxella cuniculi
@Moraxella_Moraxellaceae_Pseudomonadales_Gammaproteobacteria_Proteobacteria_Bacteria
WP_076555797:0.0683554105)98:0.0343602493)87:0.0172463264)54:0.0082892006)89:0.0223757053,Stenoxybacter
acetivorans @Stenoxybacter_Neisseriaceae_Neisseriales_Betaproteobacteria_Proteobacteria_Bacteria
WP_037587031:0.1093140333)44:0.0106716658,(Haemophilus massiliensis
@Haemophilus_Pasteurellaceae_Pasteurellales_Gammaproteobacteria_Proteobacteria_Bacteria
WP_032108847:0.0258562600,Necropsobacter rosorum
@Pasteurellaceae_Pasteurellales_Gammaproteobacteria_Proteobacteria_Bacteria
WP_032092002:0.0391795618)100:0.0934981399)84:0.0314414979,Lysobacter tolerans
@Lysobacter_Xanthomonadaceae_Xanthomonadales_Gammaproteobacteria_Proteobacteria_Bacteria
WP_076585075:0.1502531165)65:0.0000029980,(((((Brachymonas chironomi
@Brachymonas_Comamonadaceae_Burkholderiales_Betaproteobacteria_Proteobacteria_Bacteria
WP_018715399:0.0330937967,Ottowia thiooxydans
@Ottowia_Comamonadaceae_Burkholderiales_Betaproteobacteria_Proteobacteria_Bacteria
WP_028601395:0.0992156329)94:0.0330980375,(Comamonas badia
@Comamonas_Comamonadaceae_Burkholderiales_Betaproteobacteria_Proteobacteria_Bacteria
WP_024539421:0.1118955930,(Methylobacillus rhizosphaerae
@Methylobacillus_Methylophilaceae_Nitrosomonadales_Betaproteobacteria_Proteobacteria_Bacteria
WP_089374667:0.0409031731,Thauera butanivorans
@Thauera_Zoogloeaceae_Rhodocyclales_Betaproteobacteria_Proteobacteria_Bacteria
WP_068639643:0.0464917459)57:0.0049035177)61:0.0153645753)75:0.0453605902,Halomonas halocynthiae
@Halomonas_Halomonadaceae_Oceanospirillales_Gammaproteobacteria_Proteobacteria_Bacteria
WP_027966272:0.1775214463)100:0.0563834136,((((Marinobacter aromaticivorans
@Marinobacter_Alteromonadaceae_Alteromonadales_Gammaproteobacteria_Proteobacteria_Bacteria
WP_100688184:0.0183015274,Marinospirillum insulare
@Marinospirillum_Oceanospirillales_Gammaproteobacteria_Proteobacteria_Bacteria
WP_027851800:0.0277898974)100:0.0212946209,Pseudorhodobacter aquimaris
@Pseudorhodobacter_Rhodobacteraceae_Rhodobacterales_Alphaproteobacteria_Proteobacteria_Bacteria
WP_050525797:0.0470436244)100:0.1509855146,Sphingomonas histidinilytica
@Sphingomonadaceae_Sphingomonadales_Alphaproteobacteria_Proteobacteria_Bacteria
WP_079646465:0.0564248510)100:0.0294067779,(Sphingobium cloacae
@Sphingobium_Sphingomonadaceae_Sphingomonadales_Alphaproteobacteria_Proteobacteria_Bacteria
WP_066515242:0.0303662278,Sphingopyxis granuli
@Sphingopyxis_Sphingomonadaceae_Sphingomonadales_Alphaproteobacteria_Proteobacteria_Bacteria
WP_076074723:0.0230732006)98:0.0155765616)99:0.0309179230)99:0.0273331323,(((Lampropedia cohaerens
@Lampropedia_Comamonadaceae_Burkholderiales_Betaproteobacteria_Proteobacteria_Bacteria
WP_046740293:0.0459990358,Thermomonas fusca

@Thermomonas_Xanthomonadaceae_Xanthomonadales_Gammaproteobacteria_Proteobacteria_Bacteria
WP_028838828:0.0526876340)100:0.0308060872,Luteimonas mephitis
@Luteimonas_Xanthomonadaceae_Xanthomonadales_Gammaproteobacteria_Proteobacteria_Bacteria
WP_027081272:0.0332822989)96:0.0184660990,Xenophilus azovorans
@Xenophilus_Comamonadaceae_Burkholderiales_Betaproteobacteria_Proteobacteria_Bacteria
WP_038216207:0.0357593568)100:0.0290982405)72:0.0153958369)99:0.0354456132,(((((Bdellovibrio exovorus
@Bdellovibrio_Bdellovibrionaceae_Bdellovibrionales_Oligoflexia_Proteobacteria_Bacteria
WP_015468947:0.0831651284,Berkiella aquae @Candidatus
Berkiella_Coxiellaceae_Legionellales_Gammaproteobacteria_Proteobacteria_Bacteria
WP_075066858:0.0302546872)100:0.1083726533,(Pseudomonas geniculata
@Xanthomonadaceae_Xanthomonadales_Gammaproteobacteria_Proteobacteria_Bacteria
WP_057502581:0.0404393846,Stenotrophomonas humi
@Stenotrophomonas_Xanthomonadaceae_Xanthomonadales_Gammaproteobacteria_Proteobacteria_Bacteria
WP_057635794:0.0692896434)100:0.1796830798)86:0.0176324232,Lysobacter enzymogenes
@Lysobacter_Xanthomonadaceae_Xanthomonadales_Gammaproteobacteria_Proteobacteria_Bacteria
WP_096377497:0.1230338317)81:0.0314383401,Novosphingobium rosa
@Novosphingobium_Sphingomonadaceae_Sphingomonadales_Alphaproteobacteria_Proteobacteria_Bacteria
WP_068090466:0.1627545114)77:0.0026067918,Bordetella bronchialis
@Bordetella_Alcaligenaceae_Burkholderiales_Betaproteobacteria_Proteobacteria_Bacteria
WP_066669734:0.1183152103)31:0.0058071929)98:0.0393730745)100:0.0722822968,((((((((Acidovorax caeni
@Acidovorax_Comamonadaceae_Burkholderiales_Betaproteobacteria_Proteobacteria_Bacteria
WP_054257882:0.0420516808,Melaminivora alkalimesophila
@Melaminivora_Comamonadaceae_Burkholderiales_Betaproteobacteria_Proteobacteria_Bacteria
WP_019373065:0.0131720436)100:0.0092731700,Diaphorobacter polyhydroxybutyrativorans
@Diaphorobacter_Comamonadaceae_Burkholderiales_Betaproteobacteria_Proteobacteria_Bacteria
WP_088886811:0.0303306751)100:0.0355367604,Comamonas composti
@Comamonas_Comamonadaceae_Burkholderiales_Betaproteobacteria_Proteobacteria_Bacteria
WP_027014979:0.0595141421)100:0.0621103181,Delftia acidovorans
@Delftia_Comamonadaceae_Burkholderiales_Betaproteobacteria_Proteobacteria_Bacteria
WP_012206071:0.0594795215)100:0.1179711972,Lampropedia hyalina
@Lampropedia_Comamonadaceae_Burkholderiales_Betaproteobacteria_Proteobacteria_Bacteria
WP_073356838:0.0723775111)100:0.1142543937,(((((((Actinosynnema mirum
@Pseudonocardiaceae_Pseudonocardiales_Actinobacteria_Bacteria WP_015803151:0.1904733910,Umezawaea
tangerina @Umezawaea_Pseudonocardiaceae_Pseudonocardiales_Actinobacteria_Bacteria
WP_106189435:0.0759253006)74:0.0255279362,(Cryptosporangium arvum
@Cryptosporangium_Cryptosporangiaceae_Frankiales_Actinobacteria_Bacteria WP_035858008:0.1247797982,Lentzea
albida @Lentzea_Pseudonocardiaceae_Pseudonocardiales_Actinobacteria_Bacteria
WP_089920021:0.1173654935)74:0.0420986371)100:0.0461531456,(Amycolatopsis jejuensis
@Amycolatopsis_Pseudonocardiaceae_Pseudonocardiales_Actinobacteria_Bacteria
WP_033288641:0.1221834266,Mycobacterium aquaticum
@Mycobacterium_Mycobacteriaceae_Corynebacteriales_Actinobacteria_Bacteria
WP_083165279:0.1254877821)100:0.0440695765)100:0.0449790325,(Knoellia sinensis
@Knoellia_Intrasporangiaceae_Micrococcales_Actinobacteria_Bacteria WP_035918038:0.0557577049,Nocardioides
jensenii @Nocardioides_Nocardioidaceae_Propionibacteriales_Actinobacteria_Bacteria
WP_067429896:0.0871581532)100:0.0426110921)100:0.0493825591,(Glycomyces fuscus
@Glycomyces_Glycomycetaceae_Glycomycetales_Actinobacteria_Bacteria WP_097637414:0.0215303266,Nocardiopsis
halotolerans @Nocardiopsis_Nocardiopsaceae_Streptosporangiales_Actinobacteria_Bacteria
WP_017571648:0.0497987867)100:0.0493527173)98:0.0121588361,Rhodococcus aetherivorans
@Rhodococcus_Nocardiaceae_Corynebacteriales_Actinobacteria_Bacteria

WP_029546750:0.1605815430)99:0.0251267668,((Nakamurella lactea
@Nakamurella_Nakamurellaceae_Nakamurellales_Actinobacteria_Bacteria
WP_029135493:0.0544420481,Streptomyces flavovirens
@Streptomyces_Streptomycetaceae_Streptomycetales_Actinobacteria_Bacteria
WP_030638643:0.0880250927)100:0.0502710640,Ruania albidiflava
@Ruania_Ruaniaceae_Micrococcales_Actinobacteria_Bacteria
WP_022916619:0.2389986871)100:0.0633921173)100:0.0432628513)100:0.0438244627,(((Marinobacter salexigens
@Marinobacter_Alteromonadaceae_Alteromonadales_Gammaproteobacteria_Proteobacteria_Bacteria
WP_100641148:0.0347997028,Marinospirillum minutulum
@Marinospirillum_Oceanospirillales_Gammaproteobacteria_Proteobacteria_Bacteria
WP_027848265:0.0271056516)100:0.0358988968,(Oblitimonas alkaliphila
@Oblitimonas_Pseudomonadaceae_Pseudomonadales_Gammaproteobacteria_Proteobacteria_Bacteria
WP_053103017:0.0232942146,Thiopseudomonas denitrificans
@Thiopseudomonas_Pseudomonadaceae_Pseudomonadales_Gammaproteobacteria_Proteobacteria_Bacteria
WP_101497933:0.0489792709)100:0.0208112063)100:0.1226958023,Pseudomonas flexibilis
@Pseudomonas_Pseudomonadaceae_Pseudomonadales_Gammaproteobacteria_Proteobacteria_Bacteria
WP_039607332:0.1429870335)63:0.0147850970)63:0.0156870352,((((((Arthrobacter castelli
@Arthrobacter_Micrococcaceae_Micrococcales_Actinobacteria_Bacteria
WP_026817909:0.1113771958,Pseudonocardia ammonioxydans
@Pseudonocardia_Pseudonocardiaceae_Pseudonocardiales_Actinobacteria_Bacteria
WP_093339306:0.0319206997)99:0.0294700375,(Demetria terragena
@Demetria_Dermacoccaceae_Micrococcales_Actinobacteria_Bacteria WP_018157335:0.1847880646,Streptomyces
antibioticus @Streptomyces_Streptomycetaceae_Streptomycetales_Actinobacteria_Bacteria
WP_059197686:0.0386545772)96:0.0271255079)68:0.0148178155,(Saccharomonospora azurea
@Saccharomonospora_Pseudonocardiaceae_Pseudonocardiales_Actinobacteria_Bacteria
WP_005438011:0.0258574210,Saccharopolyspora antimicrobica
@Saccharopolyspora_Pseudonocardiaceae_Pseudonocardiales_Actinobacteria_Bacteria
WP_093155793:0.0577320591)72:0.0243598463)74:0.0203530560,Pseudonocardia asaccharolytica
@Pseudonocardia_Pseudonocardiaceae_Pseudonocardiales_Actinobacteria_Bacteria
WP_084795917:0.0715551250)49:0.0102417636,(((Intrasporangium calvum
@Intrasporangium_Intrasporangiaceae_Micrococcales_Actinobacteria_Bacteria
WP_013494200:0.0653666904,(Kineosphaera limosa
@Kineosphaera_Dermatophilaceae_Micrococcales_Actinobacteria_Bacteria WP_006590879:0.1183798882,Piscicoccus
intestinalis @Piscicoccus_Dermatophilaceae_Micrococcales_Actinobacteria_Bacteria
WP_068428505:0.0545591938)100:0.0645736213)100:0.0266949663,Tetrasphaera jenkinsii
@Tetrasphaera_Intrasporangiaceae_Micrococcales_Actinobacteria_Bacteria
WP_048546513:0.2362648686)75:0.0460965321,(Rhodococcus maanshanensis
@Rhodococcus_Nocardiaceae_Corynebacteriales_Actinobacteria_Bacteria WP_072751139:0.0446896387,Tomitella
biformata @Tomitella_Corynebacteriales_Actinobacteria_Bacteria
WP_024793701:0.0764320260)100:0.0752329370)66:0.0313239734)100:0.0199766331,(Lysobacter defluvii
@Lysobacter_Xanthomonadaceae_Xanthomonadales_Gammaproteobacteria_Proteobacteria_Bacteria
WP_052106504:0.0935592571,Mycobacterium agri
@Mycolicibacterium_Mycobacteriaceae_Corynebacteriales_Actinobacteria_Bacteria
WP_097940642:0.1922620570)100:0.0575274815)98:0.0333603725)63:0.0135297169)96:0.0319721242,(Amantichitinu
m ursilacus @Amantichitinum_Neisseriaceae_Neisseriales_Betaproteobacteria_Proteobacteria_Bacteria
WP_053936438:0.1539577350,Vibrio shilonii
@Vibrio_Vibrionaceae_Vibrionales_Gammaproteobacteria_Proteobacteria_Bacteria
WP_088878057:0.2958517304)97:0.0700493547)88:0.0254003566,((((Cohaesibacter gelatinilyticus
@Cohaesibacteraceae_Rhizobiales_Alphaproteobacteria_Proteobacteria_Bacteria

WP_097153957:0.0958174961,Kiloniella majae
@Kiloniella_Kiloniellaceae_Kiloniellales_Alphaproteobacteria_Proteobacteria_Bacteria
WP_085908742:0.1158703809)100:0.0283481395,((Phaeobacter gallaeciensis
@Phaeobacter_Rhodobacteraceae_Rhodobacterales_Alphaproteobacteria_Proteobacteria_Bacteria
WP_024096627:0.0226753140,Pseudovibrio ascidiaceicola
@Pseudovibrio_Rhodobacteraceae_Rhodobacterales_Alphaproteobacteria_Proteobacteria_Bacteria
WP_093521375:0.1519933348)93:0.0239035016,Ruegeria conchae
@Ruegeria_Rhodobacteraceae_Rhodobacterales_Alphaproteobacteria_Proteobacteria_Bacteria
WP_029622082:0.0664318471)93:0.0321625193)100:0.0412550829,(Dyadobacter fermentans
@Dyadobacter_Cytophagaceae_Cytophagales_Cytophagia_Bacteroidetes_Bacteria
WP_015811294:0.2169805854,Mucilaginibacter gossypiicola
@Mucilaginibacter_Sphingobacteriaceae_Sphingobacteriales_Sphingobacteriia_Bacteroidetes_Bacteria
WP_091214188:0.0884028249)99:0.1347139920)85:0.0379465190,(Plesiomonas shigelloides
@Plesiomonas_unclassified Enterobacterales_Enterobacterales_Gammaproteobacteria_Proteobacteria_Bacteria
WP_039044974:0.1111944904,Shewanella woodyi
@Shewanella_Shewanellaceae_Alteromonadales_Gammaproteobacteria_Proteobacteria_Bacteria
WP_012326469:0.2865055125)96:0.1334403329)41:0.0061430968)51:0.0139859100)3:0.0086608080,((((((((Aequorivit
a viscosa @Aequorivita_Flavobacteriaceae_Flavobacteriales_Flavobacteriia_Bacteroidetes_Bacteria
WP_073219245:0.1217042923,(Brumimicrobium mesophilum
@Brumimicrobium_Crocinitomicaceae_Flavobacteriales_Flavobacteriia_Bacteroidetes_Bacteria
WP_107039984:0.0501030084,Salegentibacter echinorum
@Salegentibacter_Flavobacteriaceae_Flavobacteriales_Flavobacteriia_Bacteroidetes_Bacteria
WP_072875679:0.2968461594)100:0.0310321589)100:0.0822767299,Flavobacterium columnare
@Flavobacterium_Flavobacteriaceae_Flavobacteriales_Flavobacteriia_Bacteroidetes_Bacteria
WP_088399596:0.2066725364)100:0.0701379893,(Polaromonas naphthalenivorans
@Polaromonas_Comamonadaceae_Burkholderiales_Betaproteobacteria_Proteobacteria_Bacteria
WP_011802041:0.1004453352,Ramlibacter tataouinensis
@Ramlibacter_Comamonadaceae_Burkholderiales_Betaproteobacteria_Proteobacteria_Bacteria
WP_061502514:0.1901072262)100:0.0723850726)91:0.0210224889,(((Magnetofaba australis
@Magnetofaba_Magnetococcaceae_Magnetococcales_Alphaproteobacteria_Proteobacteria_Bacteria
WP_085442659:0.0978743931,Thioploca ingrica
@Thioploca_Thiotrichaceae_Thiotrichales_Gammaproteobacteria_Proteobacteria_Bacteria
WP_045474072:0.0737261314)100:0.0217226444,Sedimenticola sp.
@Sedimenticola_Gammaproteobacteria_Proteobacteria_Bacteria
PLY16119:0.0449271506)100:0.0479225397,(Nitrosomonas marina
@Nitrosomonas_Nitrosomonadaceae_Nitrosomonadales_Betaproteobacteria_Proteobacteria_Bacteria
WP_090627803:0.1689538870,Thauera terpenica
@Thauera_Zoogloeaceae_Rhodocyclales_Betaproteobacteria_Proteobacteria_Bacteria
WP_021249038:0.0816542454)98:0.0516219482)97:0.0442386624)81:0.0056130627,((Kangiella geojedonensis
@Kangiella_Kangiellaceae_Oceanospirillales_Gammaproteobacteria_Proteobacteria_Bacteria
WP_046561806:0.1861639372,Methylophaga muralis
@Methylophaga_Piscirickettsiaceae_Thiotrichales_Gammaproteobacteria_Proteobacteria_Bacteria
WP_069295827:0.1358664686)81:0.0338631175,Psychromonas arctica
@Psychromonas_Psychromonadaceae_Alteromonadales_Gammaproteobacteria_Proteobacteria_Bacteria
WP_028867180:0.1522812826)88:0.0190607178)99:0.0427981582,Nitrosomonas europaea
@Nitrosomonas_Nitrosomonadaceae_Nitrosomonadales_Betaproteobacteria_Proteobacteria_Bacteria
KXK47190:0.1036252430)26:0.0099523251,(Colwellia aestuarii
@Colwellia_Colwelliaceae_Alteromonadales_Gammaproteobacteria_Proteobacteria_Bacteria
WP_077288174:0.1829761799,Oceanisphaera profunda

@Oceanisphaera_Aeromonadaceae_Aeromonadales_Gammaproteobacteria_Proteobacteria_Bacteria
WP_087037535:0.0846280461)94:0.0677940014)21:0.0114808953,((((Agarilytica rhodophyticola
@Agarilytica_Cellvibrionaceae_Cellvibrionales_Gammaproteobacteria_Proteobacteria_Bacteria
WP_086930740:0.1120906354,(Amphritea japonica
@Amphritea_Oceanospirillales_Gammaproteobacteria_Proteobacteria_Bacteria
WP_019623314:0.1200668237,Marinobacterium jannaschii
@Marinobacterium_Oceanospirillales_Gammaproteobacteria_Proteobacteria_Bacteria
WP_027857710:0.1327047135)99:0.0149638208)100:0.0585299291,((Methylotenera versatilis
@Methylotenera_Methylophilaceae_Nitrosomonadales_Betaproteobacteria_Proteobacteria_Bacteria
WP_013146757:0.0563639487,Propionivibrio
@Propionivibrio_Rhodocyclaceae_Rhodocyclales_Betaproteobacteria_Proteobacteria_Bacteria
SBT06867:0.0742997570)99:0.0283736329,Novimethylophilus kurashikiensis
@Novimethylophilus_Methylophilaceae_Nitrosomonadales_Betaproteobacteria_Proteobacteria_Bacteria
GBG15006:0.0599783599)98:0.0388395340)93:0.0048108067,(((((Aliivibrio fischeri
@Aliivibrio_Vibrionaceae_Vibrionales_Gammaproteobacteria_Proteobacteria_Bacteria
WP_065604460:0.0086384669,(((Moritella viscosa
@Moritella_Moritellaceae_Alteromonadales_Gammaproteobacteria_Proteobacteria_Bacteria
WP_075476635:0.0562517807,(Photobacterium swingsii
@Photobacterium_Vibrionaceae_Vibrionales_Gammaproteobacteria_Proteobacteria_Bacteria
WP_048900285:0.0128868812,Vibrio tapetis
@Vibrio_Vibrionaceae_Vibrionales_Gammaproteobacteria_Proteobacteria_Bacteria
WP_102522428:0.0455707755)99:0.0048159783)100:0.0604714868,Vibrio splendidus
@Vibrio_Vibrionaceae_Vibrionales_Gammaproteobacteria_Proteobacteria_Bacteria
WP_060980655:0.0188110160)32:0.0092937666,Photobacterium iliopiscarium
@Photobacterium_Vibrionaceae_Vibrionales_Gammaproteobacteria_Proteobacteria_Bacteria
WP_107237855:0.0415796717)100:0.0172976533)100:0.0632109085,(Photobacterium rosenbergii
@Photobacterium_Vibrionaceae_Vibrionales_Gammaproteobacteria_Proteobacteria_Bacteria
WP_107298421:0.0886286856,Vibrio rotiferianus
@Vibrio_Vibrionaceae_Vibrionales_Gammaproteobacteria_Proteobacteria_Bacteria
WP_071235047:0.0672190793)100:0.0912303562)100:0.0253167372,(Colwellia echini
@Colwellia_Colwelliaceae_Alteromonadales_Gammaproteobacteria_Proteobacteria_Bacteria
WP_101343171:0.1014776867,Pseudoalteromonas denitrificans
@Pseudoalteromonas_Pseudoalteromonadaceae_Alteromonadales_Gammaproteobacteria_Proteobacteria_Bacteria
WP_091984046:0.1177106776)100:0.0348120749)100:0.0329327239,(Contendobacter odensis @Candidatus
Contendobacter_Competibacteraceae_Gammaproteobacteria_Proteobacteria_Bacteria
WP_034437035:0.0552343441,Nitrospira japonica @Nitrospira_Nitrospiraceae_Nitrospirales_Nitrospirae_Bacteria
WP_080886730:0.1042100763)100:0.0445431056)58:0.0061550303,Insolitispirillum peregrinum
@Insolitispirillum_Rhodospirillaceae_Rhodospirillales_Alphaproteobacteria_Proteobacteria_Bacteria
WP_076400359:0.1147315775)95:0.0206760835)94:0.0275503007,Nitrosospira multiformis
@Nitrosomonadaceae_Nitrosomonadales_Betaproteobacteria_Proteobacteria_Bacteria
WP_074631285:0.0640176131)89:0.0106300270)64:0.0191312617)55:0.0099176356,Herminiimonas arsenicoxydans
@Herminiimonas_Oxalobacteraceae_Burkholderiales_Betaproteobacteria_Proteobacteria_Bacteria
WP_011870712:0.1470256402)12:0.0100569064,(((Aromatoleum aromaticum
@Aromatoleum_Rhodocyclaceae_Rhodocyclales_Betaproteobacteria_Proteobacteria_Bacteria
WP_011237006:0.0877144107,Azotobacter chroococcum
@Azotobacter_Pseudomonadaceae_Pseudomonadales_Gammaproteobacteria_Proteobacteria_Bacteria
WP_039804619:0.0944003809)65:0.0480219642,Paraburkholderia oxyphila
@Paraburkholderia_Burkholderiaceae_Burkholderiales_Betaproteobacteria_Proteobacteria_Bacteria
WP_028220834:0.1212696257)37:0.0087790606,Sterolibacterium denitrificans

@Sterolibacterium_Sterolibacteriaceae_Nitrosomonadales_Betaproteobacteria_Proteobacteria_Bacteria
SMB24385:0.1201993481)25:0.0067596281)97:0.0341203573,(((((((((((((((Acidiphilium cryptum
@Acidiphilium_Acetobacteraceae_Rhodospirillales_Alphaproteobacteria_Proteobacteria_Bacteria
WP_011942027:0.0571008921,Acidisphaera rubrifaciens
@Acidisphaera_Acetobacteraceae_Rhodospirillales_Alphaproteobacteria_Proteobacteria_Bacteria
WP_048863196:0.0839736585)100:0.0514674304,Acidobacterium ailaaui
@Acidobacterium_Acidobacteriaceae_Acidobacteriales_Acidobacteria_Bacteria
WP_026442340:0.1270396641)86:0.0281321175,Acidocella aminolytica
@Acidocella_Acetobacteraceae_Rhodospirillales_Alphaproteobacteria_Proteobacteria_Bacteria
WP_048877987:0.1671379517)64:0.0181607562,Afipia broomeae
@Afipia_Bradyrhizobiaceae_Rhizobiales_Alphaproteobacteria_Proteobacteria_Bacteria
PJJ38015:0.0990360238)76:0.0216245462,Sphingopyxis fribergensis
@Sphingopyxis_Sphingomonadaceae_Sphingomonadales_Alphaproteobacteria_Proteobacteria_Bacteria
WP_039575327:0.1790619425)100:0.0473266276,(Pseudoxanthomonas dokdonensis
@Pseudoxanthomonas_Xanthomonadaceae_Xanthomonadales_Gammaproteobacteria_Proteobacteria_Bacteria
WP_057657654:0.2586117192,Sphingomonas fennica
@Sphingomonas_Sphingomonadaceae_Sphingomonadales_Alphaproteobacteria_Proteobacteria_Bacteria
WP_107395276:0.1757569417)74:0.0782789327)74:0.0194824539,(Burkholderia cepacia @Burkholderia cepacia complex_Burkholderia_Burkholderiaceae_Burkholderiales_Betaproteobacteria_Proteobacteria_Bacteria
WP_060341185:0.0586602498,Paraburkholderia bannensis
@Paraburkholderia_Burkholderiaceae_Burkholderiales_Betaproteobacteria_Proteobacteria_Bacteria
WP_027820559:0.0256111114)100:0.1337346128)50:0.0137713988,(((((Agrobacterium rhizogenes
@Agrobacterium_Rhizobium/Agrobacterium group_Rhizobiaceae_Rhizobiales_Alphaproteobacteria_Proteobacteria_Bacteria
WP_034483796:0.0549961550,Rhizobium tropici @Rhizobium/Agrobacterium group_Rhizobiaceae_Rhizobiales_Alphaproteobacteria_Proteobacteria_Bacteria
WP_047630520:0.0391106114)100:0.0676635251,Bordetella flabilis
@Bordetella_Alcaligenaceae_Burkholderiales_Betaproteobacteria_Proteobacteria_Bacteria
WP_066657172:0.0671695211)100:0.0139499685,Bradyrhizobium japonicum
@Bradyrhizobium_Bradyrhizobiaceae_Rhizobiales_Alphaproteobacteria_Proteobacteria_Bacteria
WP_049812976:0.1015482264)97:0.0710596791,Caulobacter flavus
@Caulobacter_Caulobacteraceae_Caulobacterales_Alphaproteobacteria_Proteobacteria_Bacteria
WP_101713452:0.1384525788)97:0.0362135011,(Niveispirillum cyanobacteriorum
@Niveispirillum_Rhodospirillaceae_Rhodospirillales_Alphaproteobacteria_Proteobacteria_Bacteria
WP_102114272:0.0840504620,Sorangium cellulosum
@Sorangium_Polyangiaceae_Sorangiineae_Myxococcales_Deltaproteobacteria_Proteobacteria_Bacteria
WP_104985532:0.1330801502)99:0.0258609935)91:0.0190834150)59:0.0164386632,(Agrobacterium tumefaciens
@Agrobacterium tumefaciens complex_Agrobacterium_Rhizobium/Agrobacterium group_Rhizobiaceae_Rhizobiales_Alphaproteobacteria_Proteobacteria_Bacteria
WP_060726440:0.1686229736,Rhizobium leguminosarum @Rhizobium_Rhizobium/Agrobacterium group_Rhizobiaceae_Rhizobiales_Alphaproteobacteria_Proteobacteria_Bacteria
WP_027691023:0.1317446115)100:0.2139326057)7:0.0110766687,Pseudomonas marincola
@Pseudomonas_Pseudomonadaceae_Pseudomonadales_Gammaproteobacteria_Proteobacteria_Bacteria
WP_090510702:0.1857904301)7:0.0158146624,(Aquincola tertiaricarbonis
@Aquincola_Burkholderiales_Betaproteobacteria_Proteobacteria_Bacteria
WP_083440319:0.1904606805,((Burkholderia stagnalis @Burkholderia cepacia complex_Burkholderia_Burkholderiaceae_Burkholderiales_Betaproteobacteria_Proteobacteria_Bacteria
WP_060159306:0.0185878010,Cupriavidus necator
@Cupriavidus_Burkholderiaceae_Burkholderiales_Betaproteobacteria_Proteobacteria_Bacteria

WP_011617236:0.0366985554)100:0.1510883581,Pelomonas puraquae
@Pelomonas_Comamonadaceae_Burkholderiales_Betaproteobacteria_Proteobacteria_Bacteria
WP_088482193:0.1861640283)100:0.0451403605)91:0.0631680655)7:0.0239392772,Sphingobium amiense
@Sphingobium_Sphingomonadaceae_Sphingomonadales_Alphaproteobacteria_Proteobacteria_Bacteria
WP_066697727:0.3158829240)95:0.0720039280,(Caballeronia glathei
@Caballeronia_Burkholderiaceae_Burkholderiales_Betaproteobacteria_Proteobacteria_Bacteria
CDY78178:0.1660803499,((Candidimonas bauzanensis
@Candidimonas_Alcaligenaceae_Burkholderiales_Betaproteobacteria_Proteobacteria_Bacteria
WP_073103036:0.0660811852,Pusillimonas noertemannii
@Pusillimonas_Alcaligenaceae_Burkholderiales_Betaproteobacteria_Proteobacteria_Bacteria
WP_017523010:0.1175078688)100:0.0490261928,Castellaniella defragrans
@Castellaniella_Alcaligenaceae_Burkholderiales_Betaproteobacteria_Proteobacteria_Bacteria
WP_043684708:0.0873451095)100:0.0830486239)95:0.0298764910)44:0.0205793449,(Pseudomonas citronellolis
@Pseudomonas_Pseudomonadaceae_Pseudomonadales_Gammaproteobacteria_Proteobacteria_Bacteria
KRV64091:0.1356738689,Sandaracinus amylolyticus
@Sandaracinus_Sandaracinaceae_Sorangiineae_Myxococcales_Deltaproteobacteria_Proteobacteria_Bacteria
AKF04401:0.1980588906)46:0.0438040263)67:0.0211481941,((Bradyrhizobium erythrophlei
@Bradyrhizobium_Bradyrhizobiaceae_Rhizobiales_Alphaproteobacteria_Proteobacteria_Bacteria
WP_079572443:0.1019330590,Luteibacter rhizovicinus
@Luteibacter_Rhodanobacteraceae_Xanthomonadales_Gammaproteobacteria_Proteobacteria_Bacteria
WP_063573683:0.1650341759)100:0.1057260785,((Burkholderia stabilis @Burkholderia cepacia
complex_Burkholderia_Burkholderiaceae_Burkholderiales_Betaproteobacteria_Proteobacteria_Bacteria
WP_065501452:0.0851884648,Cupriavidus metallidurans
@Cupriavidus_Burkholderiaceae_Burkholderiales_Betaproteobacteria_Proteobacteria_Bacteria
WP_011520006:0.0791296968)100:0.0789669440,Pseudomonas lutea
@Pseudomonas_Pseudomonadaceae_Pseudomonadales_Gammaproteobacteria_Proteobacteria_Bacteria
PTU61493:0.1977154705)97:0.0513170586)57:0.0133438898)62:0.0208408420,((Caballeronia sordidicola
@Caballeronia_Burkholderiaceae_Burkholderiales_Betaproteobacteria_Proteobacteria_Bacteria
WP_031357941:0.0915902489,Paraburkholderia diazotrophica
@Paraburkholderia_Burkholderiaceae_Burkholderiales_Betaproteobacteria_Proteobacteria_Bacteria
WP_090864016:0.0677019626)100:0.0820674102,Janthinobacterium lividum
@Janthinobacterium_Oxalobacteraceae_Burkholderiales_Betaproteobacteria_Proteobacteria_Bacteria
WP_034787392:0.1012360768)73:0.0326648135)85:0.0198405838,(Herbaspirillum lusitanum
@Herbaspirillum_Oxalobacteraceae_Burkholderiales_Betaproteobacteria_Proteobacteria_Bacteria
WP_016835371:0.1459333669,Pseudoduganella violaceinigra
@Pseudoduganella_Oxalobacteraceae_Burkholderiales_Betaproteobacteria_Proteobacteria_Bacteria
WP_028101340:0.2347637900)81:0.0300839860)94:0.0164837310)100:0.0434911116,(((((((((Arsenophonus nasoniae
@Arsenophonus_Morganellaceae_Enterobacterales_Gammaproteobacteria_Proteobacteria_Bacteria
WP_026822031:0.1656110088,(Cosenzaea myxofaciens
@Cosenzaea_Morganellaceae_Enterobacterales_Gammaproteobacteria_Proteobacteria_Bacteria
WP_066746698:0.0675221638,(((Moellerella wisconsensis
@Moellerella_Morganellaceae_Enterobacterales_Gammaproteobacteria_Proteobacteria_Bacteria
WP_053908085:0.1030296477,Morganella morganii
@Morganella_Morganellaceae_Enterobacterales_Gammaproteobacteria_Proteobacteria_Bacteria
KLO02150:0.0567118108)100:0.0181163605,Providencia sneebia
@Providencia_Morganellaceae_Enterobacterales_Gammaproteobacteria_Proteobacteria_Bacteria
WP_008916067:0.0921739099)100:0.0122385225,Proteus alimentorum
@Morganellaceae_Enterobacterales_Gammaproteobacteria_Proteobacteria_Bacteria
WP_099073822:0.0608516721)100:0.0354921206)100:0.0217684637)97:0.0199489025,Photorhabdus luminescens

@Photorhabdus_Morganellaceae_Enterobacterales_Gammaproteobacteria_Proteobacteria_Bacteria
WP_105395381:0.1442724435)89:0.0361785855,(Brenneria goodwinii
@Brenneria_Pectobacteriaceae_Enterobacterales_Gammaproteobacteria_Proteobacteria_Bacteria
WP_095834591:0.0545393341,Lonsdalea populi
@Lonsdalea_Pectobacteriaceae_Enterobacterales_Gammaproteobacteria_Proteobacteria_Bacteria
WP_085687100:0.1064005215)95:0.0340578410)73:0.0068083942,(((Chania multitudinisentens
@Chania_Yersiniaceae_Enterobacterales_Gammaproteobacteria_Proteobacteria_Bacteria
WP_024911385:0.0794074369,Gibbsiella quercinecans
@Gibbsiella_Enterobacteriaceae_Enterobacterales_Gammaproteobacteria_Proteobacteria_Bacteria
WP_095847996:0.0247987705)100:0.0117282577,Yersinia pestis
@Yersinia_Yersiniaceae_Enterobacterales_Gammaproteobacteria_Proteobacteria_Bacteria
WP_076638651:0.0900140054)89:0.0120252987,Serratia odorifera
@Serratia_Yersiniaceae_Enterobacterales_Gammaproteobacteria_Proteobacteria_Bacteria
WP_088499746:0.0731653019)89:0.0202469747)74:0.0000021265,(Endozoicomonas atrinae
@Endozoicomonas_Hahellaceae_Oceanospirillales_Gammaproteobacteria_Proteobacteria_Bacteria
WP_066017362:0.2483402677,((Hafnia alvei
@Hafnia_Hafniaceae_Enterobacterales_Gammaproteobacteria_Proteobacteria_Bacteria
WP_072309884:0.0088703349,Obesumbacterium proteus
@Obesumbacterium_Hafniaceae_Enterobacterales_Gammaproteobacteria_Proteobacteria_Bacteria
WP_061554737:0.0167348597)100:0.0253145767,Salmonella enterica
@Salmonella_Enterobacteriaceae_Enterobacterales_Gammaproteobacteria_Proteobacteria_Bacteria
WP_058344462:0.1526254151)100:0.0472489828)100:0.0209180737)86:0.0283705966,(Budvicia aquatica
@Budvicia_Budviciaceae_Enterobacterales_Gammaproteobacteria_Proteobacteria_Bacteria
WP_099044354:0.0801735050,((Leminorella grimontii
@Leminorella_Budviciaceae_Enterobacterales_Gammaproteobacteria_Proteobacteria_Bacteria
WP_027274394:0.0378952091,Metakosakonia massiliensis
@Metakosakonia_Enterobacteriaceae_Enterobacterales_Gammaproteobacteria_Proteobacteria_Bacteria
WP_044175758:0.1442030028)100:0.0742143628,Pragia fontium
@Pragia_Budviciaceae_Enterobacterales_Gammaproteobacteria_Proteobacteria_Bacteria
WP_074822707:0.0470804682)100:0.0490040838)91:0.0712535637)96:0.0238223606,((((Buttiauxella ferragutiae
@Enterobacteriaceae_Enterobacterales_Gammaproteobacteria_Proteobacteria_Bacteria
WP_064548608:0.0847679164,((Citrobacter koseri
@Citrobacter_Enterobacteriaceae_Enterobacterales_Gammaproteobacteria_Proteobacteria_Bacteria
CDZ82466:0.0643553371,(((Enterobacter cancerogenus @Enterobacter cloacae
complex_Enterobacter_Enterobacteriaceae_Enterobacterales_Gammaproteobacteria_Proteobacteria_Bacteria
WP_102889877:0.2138464121,Franconibacter helveticus
@Franconibacter_Enterobacteriaceae_Enterobacterales_Gammaproteobacteria_Proteobacteria_Bacteria
WP_024552497:0.0815417220)96:0.0442930340,(Enterobacter kobei @Enterobacter cloacae
complex_Enterobacter_Enterobacteriaceae_Enterobacterales_Gammaproteobacteria_Proteobacteria_Bacteria
WP_088219249:0.0434530197,Siccibacter colletis
@Siccibacter_Enterobacteriaceae_Enterobacterales_Gammaproteobacteria_Proteobacteria_Bacteria
WP_031520614:0.0753490417)93:0.0143612132)95:0.0359330259,(Klebsiella grimontii
@Klebsiella_Enterobacteriaceae_Enterobacterales_Gammaproteobacteria_Proteobacteria_Bacteria
WP_098140983:0.0718033180,Shimwellia blattae
@Shimwellia_Enterobacteriaceae_Enterobacterales_Gammaproteobacteria_Proteobacteria_Bacteria
WP_002439805:0.1944782541)95:0.0453051802)81:0.0107713802)89:0.0149677331,Trabulsiella odontotermitis
@Trabulsiella_Enterobacteriaceae_Enterobacterales_Gammaproteobacteria_Proteobacteria_Bacteria
WP_054179270:0.0500045530)94:0.0239445167)99:0.0564105973,Cedecea neteri
@Cedecea_Enterobacteriaceae_Enterobacterales_Gammaproteobacteria_Proteobacteria_Bacteria

WP_061274983:0.0969601594)96:0.0426936450,Nissabacter archeti
@Nissabacter_Yersiniaceae_Enterobacterales_Gammaproteobacteria_Proteobacteria_Bacteria
WP_072930498:0.1054886343)77:0.0234376728,((Erwinia typographi
@Erwinia_Erwiniaceae_Enterobacterales_Gammaproteobacteria_Proteobacteria_Bacteria
WP_034895082:0.0708458346,Pantoea theicola
@Mixta_Erwiniaceae_Enterobacterales_Gammaproteobacteria_Proteobacteria_Bacteria
WP_103059469:0.0633763488)100:0.0601507896,(Ewingella americana
@Ewingella_Yersiniaceae_Enterobacterales_Gammaproteobacteria_Proteobacteria_Bacteria
WP_034791731:0.0444839018,Rahnella victoriana
@Rahnella_Yersiniaceae_Enterobacterales_Gammaproteobacteria_Proteobacteria_Bacteria
WP_095922824:0.0359678398)100:0.0557556411)85:0.0060600499)87:0.0266378142)100:0.1058005004,Pseudomona
s aeruginosa @Pseudomonas_Pseudomonadaceae_Pseudomonadales_Gammaproteobacteria_Proteobacteria_Bacteria
WP_034022207:0.1277649374)65:0.0000025621,(Desulfovibrio cuneatus
@Desulfovibrio_Desulfovibrionaceae_Desulfovibrionales_Deltaproteobacteria_Proteobacteria_Bacteria
WP_027187275:0.2848630508,Haematospirillum jordaniae
@Haematospirillum_Rhodospirillaceae_Rhodospirillales_Alphaproteobacteria_Proteobacteria_Bacteria
WP_082811990:0.2117880124)89:0.0714497038)88:0.0218911671,Microvirgula aerodenitrificans
@Microvirgula_Chromobacteriaceae_Neisseriales_Betaproteobacteria_Proteobacteria_Bacteria
WP_028499216:0.0902482955)100:0.0894814704)100:0.0874842714,Helicobacter pylori
@Helicobacter_Helicobacteraceae_Campylobacterales_Epsilonproteobacteria_Proteobacteria_Bacteria
WP_000247356:0.5952343338)96:0.0175236457,(((((Achromobacter arsenitoxydans
@Achromobacter_Alcaligenaceae_Burkholderiales_Betaproteobacteria_Proteobacteria_Bacteria
WP_008157526:0.0818109107,Bordetella pertussis
@Bordetella_Alcaligenaceae_Burkholderiales_Betaproteobacteria_Proteobacteria_Bacteria
WP_019248026:0.0788056371)100:0.0527490905,Orrella dioscoreae
@Orrella_Alcaligenaceae_Burkholderiales_Betaproteobacteria_Proteobacteria_Bacteria
WP_067758050:0.1404922352)100:0.0779893343,(Alcaligenes faecalis
@Alcaligenes_Alcaligenaceae_Burkholderiales_Betaproteobacteria_Proteobacteria_Bacteria
WP_088451656:0.2703912460,Dokdonella koreensis
@Dokdonella_Rhodanobacteraceae_Xanthomonadales_Gammaproteobacteria_Proteobacteria_Bacteria
WP_067647590:0.2261295043)72:0.0705780781)64:0.0281022625,((((((((Actinobacillus seminis
@Actinobacillus_Pasteurellaceae_Pasteurellales_Gammaproteobacteria_Proteobacteria_Bacteria
WP_094946227:0.0871329888,(Glaesserella parasuis
@Glaesserella_Pasteurellaceae_Pasteurellales_Gammaproteobacteria_Proteobacteria_Bacteria
WP_005710577:0.0158862556,(Muribacter muris
@Muribacter_Pasteurellaceae_Pasteurellales_Gammaproteobacteria_Proteobacteria_Bacteria
WP_047975913:0.0187802103,Rodentibacter heylii
@Rodentibacter_Pasteurellaceae_Pasteurellales_Gammaproteobacteria_Proteobacteria_Bacteria
WP_077462335:0.0145585642)100:0.0211531457)100:0.0397352243)100:0.0278172292,Aggregatibacter
actinomycetemcomitans
@Aggregatibacter_Pasteurellaceae_Pasteurellales_Gammaproteobacteria_Proteobacteria_Bacteria
WP_005547635:0.1110382443)100:0.0437492242,(Haemophilus felis
@Pasteurellaceae_Pasteurellales_Gammaproteobacteria_Proteobacteria_Bacteria
WP_078240429:0.0155467958,Pasteurella dagmatis
@Pasteurella_Pasteurellaceae_Pasteurellales_Gammaproteobacteria_Proteobacteria_Bacteria
WP_005763114:0.0187651985)100:0.0607107053)100:0.0573459234,Gallibacterium salpingitidis
@Gallibacterium_Pasteurellaceae_Pasteurellales_Gammaproteobacteria_Proteobacteria_Bacteria
WP_066421667:0.0835985459)100:0.1780603709,Photobacterium ganghwense
@Photobacterium_Vibrionaceae_Vibrionales_Gammaproteobacteria_Proteobacteria_Bacteria

WP_047884927:0.2056723477)100:0.1247485024,Aeromonas allosaccharophila
@Aeromonas_Aeromonadaceae_Aeromonadales_Gammaproteobacteria_Proteobacteria_Bacteria
WP_042655611:0.1859115282)100:0.1451817562,Basilea psittacipulmonis
@Basilea_Alcaligenaceae_Burkholderiales_Betaproteobacteria_Proteobacteria_Bacteria
WP_038497733:0.1567740241)95:0.0658676081,Uliginosibacterium gangwonense
@Uliginosibacterium_Zoogloeaceae_Rhodocyclales_Betaproteobacteria_Proteobacteria_Bacteria
WP_018608794:0.2083100860)68:0.0592602100,((((((Burkholderia plantarii
@Burkholderia_Burkholderiaceae_Burkholderiales_Betaproteobacteria_Proteobacteria_Bacteria
WP_055138049:0.1224867479,Pseudomonas acidophila
@Pseudomonas_Pseudomonadaceae_Pseudomonadales_Gammaproteobacteria_Proteobacteria_Bacteria
WP_096721039:0.0839555176)100:0.0289411436,Paraburkholderia aromaticivorans
@Paraburkholderia_Burkholderiaceae_Burkholderiales_Betaproteobacteria_Proteobacteria_Bacteria
WP_095417440:0.0969788078)100:0.0275560598,Caballeronia udeis
@Caballeronia_Burkholderiaceae_Burkholderiales_Betaproteobacteria_Proteobacteria_Bacteria
WP_062091720:0.1101424004)100:0.1992129227,Ralstonia insidiosa
@Ralstonia_Burkholderiaceae_Burkholderiales_Betaproteobacteria_Proteobacteria_Bacteria
WP_064802752:0.1546691982)98:0.0258836722,(Cupriavidus basilensis
@Cupriavidus_Burkholderiaceae_Burkholderiales_Betaproteobacteria_Proteobacteria_Bacteria
WP_043349474:0.1326143183,Pandoraea pulmonicola
@Pandoraea_Burkholderiaceae_Burkholderiales_Betaproteobacteria_Proteobacteria_Bacteria
WP_039408455:0.1833295879)94:0.0327692523)62:0.0079799135,Laribacter hongkongensis
@Laribacter_Chromobacteriaceae_Neisseriales_Betaproteobacteria_Proteobacteria_Bacteria
WP_027824489:0.2208961941)95:0.0217626947)66:0.0302792456)98:0.0435409581,(Alistipes indistinctus
@Alistipes_Rikenellaceae_Bacteroidales_Bacteroidia_Bacteroidetes_Bacteria
WP_040294199:0.1944362134,((Butyricimonas synergistica
@Butyricimonas_Odoribacteraceae_Bacteroidales_Bacteroidia_Bacteroidetes_Bacteria
OKZ19188:0.0854709119,(Culturomica massiliensis
@Culturomica_Odoribacteraceae_Bacteroidales_Bacteroidia_Bacteroidetes_Bacteria
WP_068690703:0.0768469197,Odoribacter splanchnicus
@Odoribacter_Odoribacteraceae_Bacteroidales_Bacteroidia_Bacteroidetes_Bacteria
WP_087381414:0.0718372680)100:0.1860468520)100:0.0745893965,(Coprobacter fastidiosus
@Bacteroidia_Bacteroidetes_Bacteria WP_022391309:0.1669576352,(Massilibacteroides vaginae
@Massilibacteroides_Porphyromonadaceae_Bacteroidales_Bacteroidia_Bacteroidetes_Bacteria
WP_085537457:0.1804624821,Parabacteroides chartae
@Parabacteroides_Tannerellaceae_Bacteroidales_Bacteroidia_Bacteroidetes_Bacteria
WP_079684249:0.0834416637)89:0.0566501250)90:0.0193058193)100:0.0974492982)100:0.1536501326)95:0.041610
5801)100:0.0777815175)100:0.0644147186,(Denitrovibrio acetiphilus
@Denitrovibrio_Deferribacteraceae_Deferribacterales_Deferribacteres_Bacteria
WP_013010999:0.2597445370,Kuenenia stuttgartiensis @Candidatus Kuenenia_Candidatus Brocadiaceae_Candidatus
Brocadiales_Planctomycetia_Planctomycetes_Bacteria
CAJ72046:0.2478472881)100:0.2385100389)93:0.0276858514,(Bdellovibrio bacteriovorus
@Bdellovibrio_Bdellovibrionaceae_Bdellovibrionales_Oligoflexia_Proteobacteria_Bacteria
WP_011163349:0.1993780132,Leptospira alexanderi
@Leptospira_Leptospiraceae_Leptospirales_Spirochaetes_Bacteria
WP_020983784:0.3455894552)100:0.2924190854)15:0.0207935195,((((Adhaeribacter aquaticus
@Adhaeribacter_Hymenobacteraceae_Cytophagales_Cytophagia_Bacteroidetes_Bacteria
WP_026462307:0.1720066766,Pontibacter ramchanderi
@Pontibacter_Hymenobacteraceae_Cytophagales_Cytophagia_Bacteroidetes_Bacteria
WP_101445558:0.0720040745)97:0.0676092370,Chitinophaga pinensis

@Chitinophaga_Chitinophagaceae_Chitinophagales_Chitinophagia_Bacteroidetes_Bacteria
WP_012793430:0.1404802973)98:0.0382315662,(((((Apibacter adventoris
@Apibacter_Flavobacteriaceae_Flavobacteriales_Flavobacteriia_Bacteroidetes_Bacteria
WP_105193136:0.4049607333,Sphingobacterium paucimobilis
@Sphingobacterium_Sphingobacteriaceae_Sphingobacteriales_Sphingobacteriia_Bacteroidetes_Bacteria
WP_021068502:0.1583636437)95:0.1710638008,Solitalea canadensis
@Solitalea_Sphingobacteriaceae_Sphingobacteriales_Sphingobacteriia_Bacteroidetes_Bacteria
WP_014681870:0.1542745960)11:0.0205358448,(Pedobacter oryzae
@Pedobacter_Sphingobacteriaceae_Sphingobacteriales_Sphingobacteriia_Bacteroidetes_Bacteria
WP_026897284:0.0990553698,Solirubrum puertoriconensis @Cytophagales_Cytophagia_Bacteroidetes_Bacteria
WP_059066974:0.2918179149)70:0.0411635405)10:0.0395662459,((((((Arachidicoccus rhizosphaerae
@Arachidicoccus_Chitinophagaceae_Chitinophagales_Chitinophagia_Bacteroidetes_Bacteria
WP_091399765:0.3412873236,Mucilaginibacter polytrichastri
@Mucilaginibacter_Sphingobacteriaceae_Sphingobacteriales_Sphingobacteriia_Bacteroidetes_Bacteria
WP_074489366:0.2792851145)90:0.0533606551,Pseudosphingobacterium domesticum
@Pseudosphingobacterium_Sphingobacteriaceae_Sphingobacteriales_Sphingobacteriia_Bacteroidetes_Bacteria
WP_093332811:0.4339525662)99:0.0986282084,(((((Arsenicibacter rosenii
@Arsenicibacter_Cytophagaceae_Cytophagales_Cytophagia_Bacteroidetes_Bacteria
WP_071502926:0.1771255527,((((((Bergeyella zoohelcum
@Bergeyella_Flavobacteriaceae_Flavobacteriales_Flavobacteriia_Bacteroidetes_Bacteria
WP_002686866:0.2331239722,Cloacibacterium normanense
@Flavobacteriaceae_Flavobacteriales_Flavobacteriia_Bacteroidetes_Bacteria
WP_069800498:0.0672547994)96:0.0126099143,(Riemerella anatipestifer
@Riemerella_Flavobacteriaceae_Flavobacteriales_Flavobacteriia_Bacteroidetes_Bacteria
WP_052911601:0.1388173879,Riemerella columbina
@Riemerella_Flavobacteriaceae_Flavobacteriales_Flavobacteriia_Bacteroidetes_Bacteria
WP_018675423:0.1128605860)97:0.0256984240)99:0.0278551861,Soonwooa buanensis
@Soonwooa_Flavobacteriaceae_Flavobacteriales_Flavobacteriia_Bacteroidetes_Bacteria
WP_079667659:0.0881030959)100:0.0751791890,Elizabethkingia anophelis
@Elizabethkingia_Flavobacteriaceae_Flavobacteriales_Flavobacteriia_Bacteroidetes_Bacteria
WP_058878340:0.1475256652)100:0.1492265924,(Marinilabilia salmonicolor
@Marinilabilia_Marinilabiliaceae_Marinilabiliales_Bacteroidia_Bacteroidetes_Bacteria
WP_106152690:0.1460131190,Thermophagus xiamenensis
@Thermophagus_Marinilabiliaceae_Marinilabiliales_Bacteroidia_Bacteroidetes_Bacteria
WP_010527356:0.1269427941)100:0.2568852945)100:0.0644071961,Hymenobacter gelipurpurascens
@Hymenobacter_Hymenobacteraceae_Cytophagales_Cytophagia_Bacteroidetes_Bacteria
SNC76491:0.3073107276)98:0.0429560738)95:0.0511775890,Pedobacter steynii
@Pedobacter_Sphingobacteriaceae_Sphingobacteriales_Sphingobacteriia_Bacteroidetes_Bacteria
WP_074605928:0.2069497911)89:0.0254740917,Fluviicola taffensis
@Fluviicola_Crocinitomicaceae_Flavobacteriales_Flavobacteriia_Bacteroidetes_Bacteria
WP_013686124:0.1848223214)80:0.0215248144,(Myroides profundi
@Myroides_Flavobacteriaceae_Flavobacteriales_Flavobacteriia_Bacteroidetes_Bacteria
WP_041893026:0.2977808396,Saprospira grandis
@Saprospira_Saprospiraceae_Saprospirales_Saprospiria_Bacteroidetes_Bacteria
WP_002660980:0.2343748435)94:0.1283795112)74:0.0054425702,Dyadobacter koreensis
@Dyadobacter_Cytophagaceae_Cytophagales_Cytophagia_Bacteroidetes_Bacteria
WP_090334539:0.2520378558)94:0.0252866320)86:0.0156333368,Mucilaginibacter gotjawali
@Mucilaginibacter_Sphingobacteriaceae_Sphingobacteriales_Sphingobacteriia_Bacteroidetes_Bacteria
WP_096355717:0.1640194203)73:0.0217937874,(Cyclobacterium marinum

@Cyclobacterium_Cyclobacteriaceae_Cytophagales_Cytophagia_Bacteroidetes_Bacteria
WP_014021067:0.2529944129,Flavobacterium granuli
@Flavobacterium_Flavobacteriaceae_Flavobacteriales_Flavobacteriia_Bacteroidetes_Bacteria
WP_072938503:0.1684726139)99:0.0817981528)75:0.0243795948,Confluentibacter citreus
@Confluentibacter_Flavobacteriaceae_Flavobacteriales_Flavobacteriia_Bacteroidetes_Bacteria
WP_100616065:0.2631952364)52:0.0166206520)28:0.0316556674,(Fibrella aestuarina
@Fibrella_Cytophagaceae_Cytophagales_Cytophagia_Bacteroidetes_Bacteria WP_015056675:0.2538076186,(Gillisia marina @Gillisia_Flavobacteriaceae_Flavobacteriales_Flavobacteriia_Bacteroidetes_Bacteria
WP_029331552:0.1113158376,(Mesonia mobilis
@Mesonia_Flavobacteriaceae_Flavobacteriales_Flavobacteriia_Bacteroidetes_Bacteria
WP_027886099:0.0621820898,Zunongwangia mangrovi
@Zunongwangia_Flavobacteriaceae_Flavobacteriales_Flavobacteriia_Bacteroidetes_Bacteria
WP_092541642:0.0372395252)100:0.1781385242)100:0.0914046408)99:0.0962512044)32:0.0284696787)33:0.0571698285,Arcticibacter svalbardensis
@Arcticibacter_Sphingobacteriaceae_Sphingobacteriales_Sphingobacteriia_Bacteroidetes_Bacteria
WP_016195343:0.2325608257)100:0.3043504551)14:0.0216745543,((((Andreprevotia chitinilytica
@Andreprevotia_Chromobacteriaceae_Neisseriales_Betaproteobacteria_Proteobacteria_Bacteria
WP_035053934:0.1324791093,Chitiniphilus shinanonensis
@Chitiniphilus_Chromobacteriaceae_Neisseriales_Betaproteobacteria_Proteobacteria_Bacteria
WP_018746178:0.1512749303)100:0.1884220509,(((Commensalibacter intestini
@Acetobacteraceae_Rhodospirillales_Alphaproteobacteria_Proteobacteria_Bacteria
WP_040363793:0.2451934928,Gilliamella bombicola
@Gilliamella_Orbaceae_Orbales_Gammaproteobacteria_Proteobacteria_Bacteria
WP_091346053:0.1437817000)100:0.0462682296,Wohlfahrtiimonas larvae
@Wohlfahrtiimonas_Gammaproteobacteria_Proteobacteria_Bacteria
WP_077926859:0.2626978000)100:0.1160807935,((Izhakiella australiensis
@Izhakiella_Enterobacteriaceae_Enterobacterales_Gammaproteobacteria_Proteobacteria_Bacteria
WP_078001728:0.0985546956,Sodalis praecaptivus
@Sodalis_Pectobacteriaceae_Enterobacterales_Gammaproteobacteria_Proteobacteria_Bacteria
WP_025420757:0.1393254010)100:0.1780944008,(Pseudomonas rhodesiae
@Pseudomonas_Pseudomonadaceae_Pseudomonadales_Gammaproteobacteria_Proteobacteria_Bacteria
WP_040265615:0.0689803111,Stenotrophomonas rhizophila
@Pseudomonas_Pseudomonadaceae_Pseudomonadales_Gammaproteobacteria_Proteobacteria_Bacteria
KWW18992:0.0761841462)100:0.1130938960)79:0.0142948718)100:0.0922698529)100:0.0566465179,Chitinimonas koreensis @Chitinimonas_Burkholderiaceae_Burkholderiales_Betaproteobacteria_Proteobacteria_Bacteria
WP_028447063:0.2011205204)93:0.0301096014,((((Derxia lacustris
@Derxia_Alcaligenaceae_Burkholderiales_Betaproteobacteria_Proteobacteria_Bacteria
WP_085316700:0.2319103701,Rhizobacter gummiphilus
@Rhizobacter_Burkholderiales_Betaproteobacteria_Proteobacteria_Bacteria
WP_085753626:0.2028071614)67:0.0406514030,Hydrocarboniphaga daqingensis
@Hydrocarboniphaga_Sinobacteraceae_Nevskiales_Gammaproteobacteria_Proteobacteria_Bacteria
WP_072894211:0.2217501403)67:0.0295639520,(Duganella sacchari
@Duganella_Oxalobacteraceae_Burkholderiales_Betaproteobacteria_Proteobacteria_Bacteria
WP_072780888:0.1413448874,(Massilia timonae
@Massilia_Oxalobacteraceae_Burkholderiales_Betaproteobacteria_Proteobacteria_Bacteria
WP_005669980:0.1528163255,Rugamonas rubra
@Rugamonas_Pseudomonadaceae_Pseudomonadales_Gammaproteobacteria_Proteobacteria_Bacteria
WP_093388005:0.1122905041)98:0.0391087594)98:0.0783955436)100:0.1685025336,Polyangium brachysporum
@Burkholderiales_Betaproteobacteria_Proteobacteria_Bacteria

WP_047196780:0.2727124106)93:0.0390815670)98:0.0378912096)13:0.0418804557,(((Edaphobacter aggregans
@Edaphobacter_Acidobacteriaceae_Acidobacteriales_Acidobacteria_Bacteria
WP_051978768:0.2719314761,Granulicella mallensis
@Granulicella_Acidobacteriaceae_Acidobacteriales_Acidobacteria_Bacteria
WP_014266585:0.3357420436)99:0.1073053955,(Methylobacterium populi
@Methylorubrum_Methylobacteriaceae_Rhizobiales_Alphaproteobacteria_Proteobacteria_Bacteria
WP_012452302:0.2870771143,Rhodopirellula europaea
@Rhodopirellula_Planctomycetaceae_Planctomycetales_Planctomycetia_Planctomycetes_Bacteria
WP_008661361:0.3858800961)100:0.4622527905)21:0.0487185905,Sulfurovum lithotrophicum
@Sulfurovum_Epsilonproteobacteria_Proteobacteria_Bacteria
WP_046551572:0.5654169803)11:0.0283094399)90:0.0369463821,(((((((Acidithiobacillus ferrivorans
@Acidithiobacillus_Acidithiobacillaceae_Acidithiobacillales_Acidithiobacillia_Proteobacteria_Bacteria
WP_041649679:0.2353968676,Solimonas aquatica
@Solimonas_Sinobacteraceae_Nevskiales_Gammaproteobacteria_Proteobacteria_Bacteria
WP_093283025:0.2502734202)100:0.1179783869,Sulfurifustis variabilis
@Sulfurifustis_Acidiferrobacteraceae_Acidiferrobacterales_Gammaproteobacteria_Proteobacteria_Bacteria
WP_096462460:0.1659881844)100:0.1269800523,Deinococcus grandis
@Deinococcus_Deinococcaceae_Deinococcales_Deinococci_Deinococcus-Thermus_Bacteria
WP_083524049:0.8007076821)94:0.0482934072,((Arhodomonas aquaeolei
@Arhodomonas_Ectothiorhodospiraceae_Chromatiales_Gammaproteobacteria_Proteobacteria_Bacteria
WP_018719441:0.2320592751,(Klebsormidium nitens
@Klebsormidium_Klebsormidiaceae_Klebsormidiales_Klebsormidiophyceae_Streptophyta_Viridiplantae_Eukaryota
GAQ88150:1.3027969130,Phormidesmis priestleyi Ana
@Phormidesmis_Leptolyngbyaceae_Synechococcales_Cyanobacteria_Bacteria
KPQ35711:0.8717156632)62:0.1366594424)54:0.0750397270,(Halococcus hamelinensis
@Halococcus_Halococcaceae_Halobacteriales_Halobacteria_Euryarchaeota_Archaea
WP_007692361:0.2713665916,Methanonatronarchaeum thermophilum
@Methanonatronarchaeum_Methanonatronarchaeia_Euryarchaeota_Archaea
WP_086636648:0.2978293795)100:0.3595704173)53:0.0809836687)61:0.1132145054,((((((Actinomadura hibisca
@Actinomadura_Thermomonosporaceae_Streptosporangiales_Actinobacteria_Bacteria
WP_067488747:0.2410567826,(Microtetraspora glauca
@Microtetraspora_Streptosporangiaceae_Streptosporangiales_Actinobacteria_Bacteria
WP_030496405:0.0559193067,Streptomyces curacoi
@Streptomyces_Streptomycetaceae_Streptomycetales_Actinobacteria_Bacteria
WP_062149900:0.0156746300)100:0.1138610231)98:0.0698094941,(Kitasatospora azatica
@Kitasatospora_Streptomycetaceae_Streptomycetales_Actinobacteria_Bacteria
WP_035841078:0.1394763216,Streptacidiphilus jeojiense
@Streptacidiphilus_Streptomycetaceae_Streptomycetales_Actinobacteria_Bacteria
WP_030248080:0.1296224437)100:0.0481284414)100:0.1404361845,(((((Actinospica acidiphila
@Actinospica_Actinospicaceae_Catenulisporales_Actinobacteria_Bacteria WP_033272079:0.0860495013,Kitasatospora aureofaciens @Kitasatospora_Streptomycetaceae_Streptomycetales_Actinobacteria_Bacteria
WP_052840784:0.0686100721)94:0.0286596146,(Kitasatospora albolonga
@Kitasatospora_Streptomycetaceae_Streptomycetales_Actinobacteria_Bacteria
WP_084749156:0.0694502448,Streptomyces roseus
@Streptomyces_Streptomycetaceae_Streptomycetales_Actinobacteria_Bacteria
WP_048477492:0.0472491419)100:0.0346925626)94:0.0349268950,Streptosporangium nondiastaticum
@Streptosporangium_Streptosporangiaceae_Streptosporangiales_Actinobacteria_Bacteria
WP_106682140:0.0673628501)94:0.0278294922,(Glycomyces sambucus
@Glycomyces_Glycomycetaceae_Glycomycetales_Actinobacteria_Bacteria

WP_091049111:0.1734183268,Haloglycomyces albus
@Haloglycomyces_Glycomycetaceae_Glycomycetales_Actinobacteria_Bacteria
WP_025273429:0.0774707696)100:0.0624887007)100:0.1186237662,Streptomyces gilvigriseus
@Streptomyces_Streptomycetaceae_Streptomycetales_Actinobacteria_Bacteria
WP_071656840:0.0916826942)100:0.1568637070)100:0.1106514295,Variovorax paradoxus
@Variovorax_Comamonadaceae_Burkholderiales_Betaproteobacteria_Proteobacteria_Bacteria
WP_012745931:0.2928737524)93:0.0646848457,(Corallococcus coralloides
@Corallococcus_Myxococcaceae_Cystobacterineae_Myxococcales_Deltaproteobacteria_Proteobacteria_Bacteria
WP_014399525:0.0754684723,((Cystobacter fuscus
@Cystobacter_Archangiaceae_Cystobacterineae_Myxococcales_Deltaproteobacteria_Proteobacteria_Bacteria
WP_095990067:0.0730974098,Melittangium boletus
@Melittangium_Archangiaceae_Cystobacterineae_Myxococcales_Deltaproteobacteria_Proteobacteria_Bacteria
WP_095976680:0.0707224850)100:0.1088759209,Stigmatella erecta
@Stigmatella_Archangiaceae_Cystobacterineae_Myxococcales_Deltaproteobacteria_Proteobacteria_Bacteria
WP_093515652:0.0452054668)100:0.0130259063)100:0.2548653343)100:0.1892787740,(((Allonocardiopsis opalescens
@Allonocardiopsis_Streptosporangiales_Actinobacteria_Bacteria WP_106243912:0.1999259634,((Herbidospora
cretacea @Herbidospora_Streptosporangiaceae_Streptosporangiales_Actinobacteria_Bacteria
WP_030456182:0.0942228667,Streptosporangium subroseum
@Streptosporangium_Streptosporangiaceae_Streptosporangiales_Actinobacteria_Bacteria
WP_089208948:0.1302162777)85:0.0440757771,Thermobispora bispora @Thermobispora_Actinobacteria incertae
sedis_Actinobacteria_Bacteria WP_013130501:0.1463240175)60:0.0252814853)73:0.0493970052,Nonomuraea
maritima @Nonomuraea_Streptosporangiaceae_Streptosporangiales_Actinobacteria_Bacteria
WP_090771307:0.0955944584)100:0.3678657492,((((((Amycolatopsis orientalis
@Pseudonocardiaceae_Pseudonocardiales_Actinobacteria_Bacteria WP_043833518:0.1950231010,Gilvimarinus
agarilyticus @Gilvimarinus_Cellvibrionaceae_Cellvibrionales_Gammaproteobacteria_Proteobacteria_Bacteria
WP_041521924:0.1597603655)100:0.0525494397,Pseudomonas pachastrellae
@Pseudomonas_Pseudomonadaceae_Pseudomonadales_Gammaproteobacteria_Proteobacteria_Bacteria
SFM12811:0.1289166074)100:0.0222696399,((Desulfonatronovibrio hydrogenovorans
@Desulfonatronovibrio_Desulfohalobiaceae_Desulfovibrionales_Deltaproteobacteria_Proteobacteria_Bacteria
WP_028575723:0.0859858678,Geoalkalibacter subterraneus
@Geoalkalibacter_Geobacteraceae_Desulfuromonadales_Deltaproteobacteria_Proteobacteria_Bacteria
WP_040198876:0.0827969061)100:0.1345447494,Salinicola tamaricis
@Salinicola_Halomonadaceae_Oceanospirillales_Gammaproteobacteria_Proteobacteria_Bacteria
WP_106419582:0.2078145028)100:0.0999924794)100:0.1190811780,(Brackiella oedipodis
@Brackiella_Alcaligenaceae_Burkholderiales_Betaproteobacteria_Proteobacteria_Bacteria
WP_028357436:0.3458250201,((Pseudoclavibacter helvolus
@Pseudoclavibacter_Microbacteriaceae_Micrococcales_Actinobacteria_Bacteria
WP_068479326:0.3770110309,Saccharomonospora viridis
@Saccharomonospora_Pseudonocardiaceae_Pseudonocardiales_Actinobacteria_Bacteria
WP_015786373:0.0558282411)89:0.0173509210,Thermobifida fusca
@Thermobifida_Nocardiopsaceae_Streptosporangiales_Actinobacteria_Bacteria
WP_081430450:0.0289718874)100:0.1413795806)100:0.1593966821)100:0.0998071329,((((((Cellulomonas gilvus
@Cellulomonas_Cellulomonadaceae_Micrococcales_Actinobacteria_Bacteria
WP_013883987:0.0540771579,Cellulosimicrobium aquatile
@Cellulosimicrobium_Promicromonosporaceae_Micrococcales_Actinobacteria_Bacteria
WP_076406559:0.0806260798)100:0.0738502953,Microbacterium humi
@Microbacterium_Microbacteriaceae_Micrococcales_Actinobacteria_Bacteria
WP_091184477:0.2066750516)58:0.0544896207,Rathayibacter tritici
@Rathayibacter_Microbacteriaceae_Micrococcales_Actinobacteria_Bacteria

WP_104272064:0.0999219580)28:0.0257527599,Curtobacterium luteum
@Curtobacterium_Microbacteriaceae_Micrococcales_Actinobacteria_Bacteria
WP_058726032:0.1449617595)45:0.0306874166,Clavibacter capsici
@Clavibacter_Microbacteriaceae_Micrococcales_Actinobacteria_Bacteria
WP_053773749:0.2036839036)100:0.1459255471,Nocardia amikacinitolerans
@Nocardia_Nocardiaceae_Corynebacteriales_Actinobacteria_Bacteria
WP_067790259:0.3345804006)47:0.0260686612)97:0.1065645127,Conexibacter woesei
@Conexibacter_Conexibacteraceae_Solirubrobacterales_Thermoleophilia_Actinobacteria_Bacteria
WP_081690720:0.4714140242)85:0.1189088061)100:0.2614935498)96:0.1037155249)95:0.0807279026,Zymomonas mobilis @Zymomonas_Sphingomonadaceae_Sphingomonadales_Alphaproteobacteria_Proteobacteria_Bacteria
WP_087379504:0.6323178663)65:0.0236882803,((((((((Algicola sagamiensis
@Algicola_Pseudoalteromonadaceae_Alteromonadales_Gammaproteobacteria_Proteobacteria_Bacteria
WP_018693143:0.2837018154,Wenyingzhuangia fucanilytica
@Wenyingzhuangia_Flavobacteriaceae_Flavobacteriales_Flavobacteriia_Bacteroidetes_Bacteria
WP_068826875:0.1892620184)100:0.0912296221,Endozoicomonas arenosclerae
@Endozoicomonas_Hahellaceae_Oceanospirillales_Gammaproteobacteria_Proteobacteria_Bacteria
WP_062266047:0.2487149603)69:0.0697059798,(Gallaecimonas xiamenensis
@Gallaecimonas_Gammaproteobacteria_Proteobacteria_Bacteria WP_008486616:0.2866378977,Zymobacter palmae
@Zymobacter_Zymobacter group_Halomonadaceae_Oceanospirillales_Gammaproteobacteria_Proteobacteria_Bacteria
WP_027705947:0.5246639136)64:0.0704718093)90:0.0402084438,Francisella philomiragia
@Francisella_Francisellaceae_Thiotrichales_Gammaproteobacteria_Proteobacteria_Bacteria
WP_012280943:0.3047168712)89:0.0228237700,(((Alteromonas marina
@Alteromonas_Alteromonadaceae_Alteromonadales_Gammaproteobacteria_Proteobacteria_Bacteria
WP_039219242:0.2073530845,Gilvimarinus chinensis
@Gilvimarinus_Cellvibrionaceae_Cellvibrionales_Gammaproteobacteria_Proteobacteria_Bacteria
WP_020210257:0.1242071277)60:0.0253621108,(Cellvibrio mixtus
@Cellvibrio_Cellvibrionaceae_Cellvibrionales_Gammaproteobacteria_Proteobacteria_Bacteria
WP_039911959:0.1798528952,((Methylobacillus glycogenes
@Methylobacillus_Methylophilaceae_Nitrosomonadales_Betaproteobacteria_Proteobacteria_Bacteria
WP_025870350:0.0771968607,Tenderia electrophaga @Candidatus
Tenderia_Chromatiaceae_Chromatiales_Gammaproteobacteria_Proteobacteria_Bacteria
ALP53021:0.1440756193)100:0.0720292653,Methylovorus glucosotrophus
@Methylovorus_Methylophilaceae_Nitrosomonadales_Betaproteobacteria_Proteobacteria_Bacteria
WP_015829419:0.0690403979)100:0.0534302882)92:0.0279303297)62:0.0561300859,Microbulbifer mangrovi
@Microbulbifer_Microbulbiferaceae_Cellvibrionales_Gammaproteobacteria_Proteobacteria_Bacteria
WP_078084362:0.2861939907)100:0.0340126954)100:0.3120206795,Piscirickettsia salmonis
@Piscirickettsia_Piscirickettsiaceae_Thiotrichales_Gammaproteobacteria_Proteobacteria_Bacteria
WP_017378214:0.3087829980)79:0.0803040743,((Enterovibrio calviensis
@Enterovibrio_Vibrionaceae_Vibrionales_Gammaproteobacteria_Proteobacteria_Bacteria
WP_017007766:0.1174548873,Shewanella morhuae
@Shewanella_Shewanellaceae_Alteromonadales_Gammaproteobacteria_Proteobacteria_Bacteria
PTA51134:0.0673008495)100:0.1827949676,Ferrimonas balearica
@Ferrimonas_Ferrimonadaceae_Alteromonadales_Gammaproteobacteria_Proteobacteria_Bacteria
WP_013344162:0.1863112088)100:0.1496328045)100:0.2293863606,((((((Calothrix brevissima
@Calothrix_Rivulariaceae_Nostocales_Cyanobacteria_Bacteria WP_096642416:0.0554772017,Cylindrospermum stagnale @Cylindrospermum_Nostocaceae_Nostocales_Cyanobacteria_Bacteria
WP_015205685:0.0543193798)99:0.0221942665,Nostoc calcicola
@Nostoc_Nostocaceae_Nostocales_Cyanobacteria_Bacteria
WP_073641506:0.0369179119)99:0.0258575535,Mastigocladopsis repens

@Mastigocladopsis_Symphyonemataceae_Nostocales_Cyanobacteria_Bacteria
WP_017316322:0.0483428661)100:0.0752645295,((Fischerella muscicola
@Fischerella_Hapalosiphonaceae_Nostocales_Cyanobacteria_Bacteria WP_016858454:0.0000022994,Mastigocladus laminosus @Mastigocladus_Hapalosiphonaceae_Nostocales_Cyanobacteria_Bacteria
WP_044448815:0.0000025164)100:0.0401597577,Oscillatoria nigro-viridis
@Oscillatoria_Oscillatoriaceae_Oscillatoriales_Oscillatoriophycideae_Cyanobacteria_Bacteria
WP_015179116:0.1448512054)89:0.0320971273)100:0.0918834829,Mastigocoleus testarum
@Mastigocoleus_Hapalosiphonaceae_Nostocales_Cyanobacteria_Bacteria
WP_027844204:0.2399689815)100:0.0789937397,((Chthonomonas calidirosea
@Chthonomonas_Chthonomonadaceae_Chthonomonadales_Chthonomonadetes_Armatimonadetes_Bacteria
WP_075163171:0.1404483636,(Rhodothermus marinus @Rhodothermus_Rhodothermaceae_Bacteroidetes Order II. Incertae sedis_Bacteroidetes_Bacteria WP_012845258:0.1035457863,Rubricoccus marinus
@Rubricoccus_Rubricoccaceae_Rhodothermales_Rhodothermia_Rhodothermaeota_Bacteria
WP_094550067:0.4048114881)91:0.0387099972)93:0.0718464658,Tolypothrix campylonemoides
@Tolypothrix_Tolypothrichaceae_Nostocales_Cyanobacteria_Bacteria
WP_041039549:0.2024643348)100:0.1691631685)93:0.1039468967)71:0.0275837844)84:0.0228582897)100:0.0946246913,((Azospirillum oryzae
@Azospirillum_Rhodospirillaceae_Rhodospirillales_Alphaproteobacteria_Proteobacteria_Bacteria
WP_085085866:0.1338319095,Inquilinus limosus
@Inquilinus_Rhodospirillaceae_Rhodospirillales_Alphaproteobacteria_Proteobacteria_Bacteria
WP_026871538:0.1028369170)91:0.0301908191,Skermanella aerolata
@Skermanella_Rhodospirillaceae_Rhodospirillales_Alphaproteobacteria_Proteobacteria_Bacteria
WP_044431318:0.1406166391)100:0.2173128543)100:0.1111325829,(((Acidibacillus ferrooxidans
@Acidibacillus_Bacillales_Bacilli_Firmicutes_Bacteria WP_067560846:0.2015930340,Paenibacillus naphthalenovorans
@Paenibacillus_Paenibacillaceae_Bacillales_Bacilli_Firmicutes_Bacteria
WP_062410260:0.1003858020)100:0.0684858177,Alicyclobacillus acidiphilus
@Alicyclobacillus_Alicyclobacillaceae_Bacillales_Bacilli_Firmicutes_Bacteria
WP_067618653:0.1763857277)100:0.0884688534,((((((Aeribacillus pallidus
@Aeribacillus_Bacillaceae_Bacillales_Bacilli_Firmicutes_Bacteria WP_094244441:0.0445794873,(Anoxybacillus tepidamans @Anoxybacillus_Bacillaceae_Bacillales_Bacilli_Firmicutes_Bacteria
WP_027409719:0.0466361488,((Geobacillus galactosidasius
@Geobacillus_Bacillaceae_Bacillales_Bacilli_Firmicutes_Bacteria WP_089098080:0.0000028719,Thermicanus aegyptius
@Thermicanus_Bacillales Family X. Incertae Sedis_Bacillales_Bacilli_Firmicutes_Bacteria
WP_005586697:0.0774645706)100:0.0041965177,Parageobacillus toebii @Bacillales_Bacilli_Firmicutes_Bacteria
WP_062678358:0.0000027704)100:0.0398880433)100:0.0825248993)100:0.0725023112,(((((((((((((Alkalibacterium pelagium @Alkalibacterium_Carnobacteriaceae_Lactobacillales_Bacilli_Firmicutes_Bacteria
WP_091480231:0.2438701386,Gracilibacillus halophilus
@Gracilibacillus_Bacillaceae_Bacillales_Bacilli_Firmicutes_Bacteria
WP_003470096:0.0839430137)70:0.0179960207,Paraliobacillus ryukyuensis
@Paraliobacillus_Bacillaceae_Bacillales_Bacilli_Firmicutes_Bacteria
WP_079707871:0.1154439446)100:0.1207800131,(((Alteribacillus iranensis
@Alteribacillus_Bacillaceae_Bacillales_Bacilli_Firmicutes_Bacteria SFE82728:0.0948078697,Virgibacillus necropolis
@Virgibacillus_Bacillaceae_Bacillales_Bacilli_Firmicutes_Bacteria
WP_089534071:0.1478069136)97:0.0312868127,(((Bacillus aidingensis
@Bacillus_Bacillaceae_Bacillales_Bacilli_Firmicutes_Bacteria WP_026702567:0.0000022398,Salibacterium qingdaonense @Bacillaceae_Bacillales_Bacilli_Firmicutes_Bacteria
WP_090925054:0.0212531378)100:0.1233384235,Marinococcus luteus
@Marinococcus_Bacillaceae_Bacillales_Bacilli_Firmicutes_Bacteria
WP_091613962:0.0921087927)100:0.0369762034,((((((Kroppenstedtia eburnea

@Kroppenstedtia_Thermoactinomycetaceae_Bacillales_Bacilli_Firmicutes_Bacteria
SIS42085:0.0284965213,(Melghirimyces thermohalophilus
@Melghirimyces_Thermoactinomycetaceae_Bacillales_Bacilli_Firmicutes_Bacteria
WP_091572558:0.0276276213,Paludifilum halophilum
@Paludifilum_Thermoactinomycetaceae_Bacillales_Bacilli_Firmicutes_Bacteria
WP_094264887:0.0999036032)72:0.0166248791)72:0.0000020845,Numidum massiliense
@Numidum_Bacillaceae_Bacillales_Bacilli_Firmicutes_Bacteria
WP_054949484:0.0311603001)100:0.0772211081,Lentibacillus persicus
@Lentibacillus_Bacillaceae_Bacillales_Bacilli_Firmicutes_Bacteria
WP_090087065:0.0544547812)100:0.0994340496,Tenuibacillus multivorans
@Tenuibacillus_Bacillaceae_Bacillales_Bacilli_Firmicutes_Bacteria
WP_093856636:0.0635170021)100:0.0795046318,Piscibacillus halophilus
@Piscibacillus_Bacillaceae_Bacillales_Bacilli_Firmicutes_Bacteria
WP_091773624:0.0278369835)99:0.0474005727,Oceanobacillus iheyensis
@Oceanobacillus_Bacillaceae_Bacillales_Bacilli_Firmicutes_Bacteria
WP_011065038:0.0996507680)45:0.0100905172)70:0.0352883315)99:0.0616372268,Salinibacillus kushneri
@Salinibacillus_Bacillaceae_Bacillales_Bacilli_Firmicutes_Bacteria
WP_093137128:0.1830178975)95:0.0461479057)100:0.0774728896,Quasibacillus thermotolerans
@Quasibacillus_Bacillaceae_Bacillales_Bacilli_Firmicutes_Bacteria
WP_039237146:0.0623686921)95:0.0122414874,Virgibacillus soli
@Virgibacillus_Bacillaceae_Bacillales_Bacilli_Firmicutes_Bacteria
WP_057986261:0.0877834727)89:0.0345516323,(Chlamydia abortus @Chlamydia_Chlamydia/Chlamydophila
group_Chlamydiaceae_Chlamydiales_Chlamydiae_Bacteria SHE15245:0.0846784708,((Cohnella kolymensis
@Cohnella_Paenibacillaceae_Bacillales_Bacilli_Firmicutes_Bacteria WP_041058476:0.0873586300,Paenibacillus
camerounensis @Paenibacillus_Paenibacillaceae_Bacillales_Bacilli_Firmicutes_Bacteria
WP_042203871:0.0640962395)99:0.0167375270,Domibacillus aminovorans
@Domibacillus_Bacillaceae_Bacillales_Bacilli_Firmicutes_Bacteria
WP_063965304:0.1301490890)7:0.0102164757)51:0.0190283503)2:0.0062438062,(Brevibacterium frigoritolerans
@Bacillus_Bacillaceae_Bacillales_Bacilli_Firmicutes_Bacteria WP_063589805:0.1519171814,Risungbinella massiliensis
@Risungbinella_Thermoactinomycetaceae_Bacillales_Bacilli_Firmicutes_Bacteria
WP_044640681:0.0458382134)2:0.0056580235)82:0.0236292890,Bacillus soli
@Bacillus_Bacillaceae_Bacillales_Bacilli_Firmicutes_Bacteria
WP_066064705:0.1536417001)94:0.0114538353,((Fictibacillus arsenicus
@Fictibacillus_Bacillaceae_Bacillales_Bacilli_Firmicutes_Bacteria WP_066290043:0.0543252076,Thalassobacillus
devorans @Thalassobacillus_Bacillaceae_Bacillales_Bacilli_Firmicutes_Bacteria
WP_028783654:0.0965677267)98:0.0280139607,((Gorillibacterium massiliense
@Gorillibacterium_Paenibacillaceae_Bacillales_Bacilli_Firmicutes_Bacteria WP_040949987:0.1123212846,Paenibacillus
alginolyticus @Paenibacillus_Paenibacillaceae_Bacillales_Bacilli_Firmicutes_Bacteria
WP_029195217:0.0701029191)97:0.0425251652,Streptococcus pneumoniae
@Streptococcus_Streptococcaceae_Lactobacillales_Bacilli_Firmicutes_Bacteria
CJF61396:0.0334741107)31:0.0123200186)36:0.0200369092)25:0.0095916997,Terribacillus aidingensis
@Terribacillus_Bacillaceae_Bacillales_Bacilli_Firmicutes_Bacteria
WP_038564789:0.0926031711)23:0.0102130196,(Bacillus testis
@Bacillus_Bacillaceae_Bacillales_Bacilli_Firmicutes_Bacteria WP_050616318:0.1379062580,Sporosarcina globispora
@Sporosarcina_Planococcaceae_Bacillales_Bacilli_Firmicutes_Bacteria
WP_053436003:0.0450690077)55:0.0413220013)81:0.0156352295,(Jeotgalibacillus soli
@Jeotgalibacillus_Planococcaceae_Bacillales_Bacilli_Firmicutes_Bacteria WP_041087390:0.0519156002,(Planococcus
kocurii @Planococcus_Planococcaceae_Bacillales_Bacilli_Firmicutes_Bacteria
WP_058386576:0.0613620680,Planomicrobium glaciei

@Planomicrobium_Planococcaceae_Bacillales_Bacilli_Firmicutes_Bacteria
WP_036802832:0.0247134560)100:0.1285137485)100:0.0814261674)63:0.0080232334,(Brevibacillus borstelensis
@Brevibacillus_Paenibacillaceae_Bacillales_Bacilli_Firmicutes_Bacteria WP_003389645:0.0268324724,Seinonella
peptonophila @Seinonella_Thermoactinomycetaceae_Bacillales_Bacilli_Firmicutes_Bacteria
WP_073152289:0.1004661902)99:0.0327072297)86:0.0156037531,(Sediminibacillus halophilus
@Sediminibacillus_Bacillaceae_Bacillales_Bacilli_Firmicutes_Bacteria WP_074600731:0.0159788713,Virgibacillus
senegalensis @Virgibacillus_Bacillaceae_Bacillales_Bacilli_Firmicutes_Bacteria
WP_053217524:0.0109062914)100:0.1225590153)86:0.0295954853,((Brochothrix campestris
@Brochothrix_Listeriaceae_Bacillales_Bacilli_Firmicutes_Bacteria WP_035314486:0.3682049191,Sporolactobacillus
vineae @Sporolactobacillus_Sporolactobacillaceae_Bacillales_Bacilli_Firmicutes_Bacteria
WP_010630572:0.1326396441)72:0.0890939203,(Carnobacterium divergens
@Carnobacterium_Carnobacteriaceae_Lactobacillales_Bacilli_Firmicutes_Bacteria
SPC41601:0.1613343862,(((Enterococcus plantarum
@Enterococcus_Enterococcaceae_Lactobacillales_Bacilli_Firmicutes_Bacteria
WP_069654240:0.1789346098,Lactobacillus curvatus
@Lactobacillus_Lactobacillaceae_Lactobacillales_Bacilli_Firmicutes_Bacteria
WP_089557236:0.3424157930)100:0.2520726003,(Floricoccus tropicus
@Floricoccus_Streptococcaceae_Lactobacillales_Bacilli_Firmicutes_Bacteria WP_070791474:0.2129574312,Weissella
minor @Weissella_Leuconostocaceae_Lactobacillales_Bacilli_Firmicutes_Bacteria
KRN77299:0.0750883038)100:0.3843287907)100:0.1255739581,Enterococcus sulfureus
@Enterococcus_Enterococcaceae_Lactobacillales_Bacilli_Firmicutes_Bacteria
WP_016186031:0.2790658747)94:0.1328714246)61:0.0430184924)69:0.0091464944)90:0.0265998056)100:0.0319440
357,Pontibacillus yanchengensis @Pontibacillus_Bacillaceae_Bacillales_Bacilli_Firmicutes_Bacteria
WP_036822790:0.2610180860)100:0.0210804361,Halobacillus hunanensis
@Halobacillus_Bacillaceae_Bacillales_Bacilli_Firmicutes_Bacteria
WP_079525393:0.1795451681)100:0.0972310647,Caenibacillus caldisaponilyticus
@Caenibacillus_Sporolactobacillaceae_Bacillales_Bacilli_Firmicutes_Bacteria
WP_077615303:0.1004900830)100:0.0748704059,Novibacillus thermophilus
@Novibacillus_Thermoactinomycetaceae_Bacillales_Bacilli_Firmicutes_Bacteria
WP_077720394:0.1372792485)98:0.0540288863)100:0.0462688098)99:0.0605376542,((Calothrix parietina
@Calothrix_Rivulariaceae_Nostocales_Cyanobacteria_Bacteria WP_015196241:0.3439169707,Rubritalea profundi
@Rubritalea_Rubritaleaceae_Verrucomicrobiales_Verrucomicrobiae_Verrucomicrobia_Bacteria
WP_105043638:0.6185465823)85:0.0318162085,Ktedonobacter racemifer
@Ktedonobacter_Ktedonobacteraceae_Ktedonobacterales_Ktedonobacteria_Chloroflexi_Bacteria
WP_007915547:0.3644547654)100:0.0886017795)100:0.0737477816,(((((Akkermansia glycaniphila
@Akkermansia_Akkermansiaceae_Verrucomicrobiales_Verrucomicrobiae_Verrucomicrobia_Bacteria
WP_067777657:0.3134417742,Mucispirillum schaedleri
@Mucispirillum_Deferribacteraceae_Deferribacterales_Deferribacteres_Bacteria
WP_023275781:0.3564461331)100:0.1027477185,Methanobrevibacter cuticularis
@Methanobrevibacter_Methanobacteriaceae_Methanobacteriales_Methanobacteria_Euryarchaeota_Archaea
WP_067259161:0.3341699529)100:0.1027281731,(Desulfotomaculum ruminis
@Desulfotomaculum_Peptococcaceae_Clostridiales_Clostridia_Firmicutes_Bacteria
WP_013841441:0.3353210611,(Dysgonomonas gadei
@Dysgonomonas_Dysgonamonadaceae_Bacteroidales_Bacteroidia_Bacteroidetes_Bacteria
WP_006800148:0.3006348031,Methanomassiliicoccus luminyensis
@Methanomassiliicoccus_Methanomassiliicoccaceae_Methanomassiliicoccales_Thermoplasmata_Euryarchaeota_Archa
ea WP_019178123:0.4353218904)68:0.0436525285)68:0.0204507479)98:0.0514134482,Deinococcus misasensis
@Deinococcus_Deinococcaceae_Deinococcales_Deinococci_Deinococcus-Thermus_Bacteria
WP_034342422:0.6367869587)98:0.0268903460,((((Desulfarculus baarsii

@Desulfarculus_Desulfarculaceae_Desulfarculales_Deltaproteobacteria_Proteobacteria_Bacteria WP_013257625:0.0715096167,(Desulfovibrio alkalitolerans @Desulfohalovibrio_Desulfovibrionaceae_Desulfovibrionales_Deltaproteobacteria_Proteobacteria_Bacteria WP_020887383:0.1206489193,(Methanococcoides vulcani @Methanococcoides_Methanosarcinaceae_Methanosarcinales_Methanomicrobia_Euryarchaeota_Archaea WP_091690036:0.0594081814,((Methanolobus psychrophilus @Methanolobus_Methanosarcinaceae_Methanosarcinales_Methanomicrobia_Euryarchaeota_Archaea WP_015053150:0.0839971537,Methanosarcina barkeri @Methanosarcina_Methanosarcinaceae_Methanosarcinales_Methanomicrobia_Stenosarchaea group_Euryarchaeota_Archaea WP_011305841:0.0515268592)100:0.0115627219,Methanosalsum zhilinae @Methanosalsum_Methanosarcinaceae_Methanosarcinales_Methanomicrobia_Stenosarchaea group_Euryarchaeota_Archaea WP_013898966:0.0702327219)100:0.0134080826)100:0.0797215416)100:0.0249982493)86:0.0125948497,Desulfobacca acetoxidans @Desulfobacca_Syntrophaceae_Syntrophobacterales_Deltaproteobacteria_Proteobacteria_Bacteria WP_013706143:0.1495177408)96:0.0297736858,Desulfocarbo indianensis @Desulfocarbo_Desulfarculaceae_Desulfarculales_Deltaproteobacteria_Proteobacteria_Bacteria WP_049673636:0.1206356591)96:0.0364488381,(Desulfocurvus vexinensis @Desulfocurvus_Desulfovibrionaceae_Desulfovibrionales_Deltaproteobacteria_Proteobacteria_Bacteria WP_028588583:0.1191967434,(Halodesulfovibrio aestuarii @Halodesulfovibrio_Desulfovibrionaceae_Desulfovibrionales_Deltaproteobacteria_Proteobacteria_Bacteria WP_027362245:0.1468686499,Pseudodesulfovibrio aespoeensis @Desulfovibrionaceae_Desulfovibrionales_Deltaproteobacteria_Proteobacteria_Bacteria WP_013513775:0.0278071054)100:0.0762862138)100:0.0871504100)100:0.2055232586)99:0.1004485690)78:0.0206452436,((((((((((((((((Acanthisitta chloris @Acanthisitta_Acanthisittidae_Passeriformes_Neognathae_Aves_Coelurosauria_Theropoda_Saurischia_Dinosauria_Archosauria_Archelosauria_Euteleostomi_Vertebrata_Craniata_Chordata_Metazoa_Eukaryota KFP73266:0.0089028638,((Corvus cornix cornix @Corvus_Corvidae_Corvoidea_Passeriformes_Neognathae_Aves_Coelurosauria_Theropoda_Saurischia_Dinosauria_Archosauria_Archelosauria_Euteleostomi_Vertebrata_Craniata_Chordata_Metazoa_Eukaryota XP_019149262:0.0048449075,(Lonchura striata domestica @Lonchura_Estrildinae_Estrildidae_Passeroidea_Passeriformes_Neognathae_Aves_Coelurosauria_Theropoda_Saurischia_Dinosauria_Archosauria_Archelosauria_Euteleostomi_Vertebrata_Craniata_Chordata_Metazoa_Eukaryota XP_021411590:0.0170761407,Manacus vitellinus @Manacus_Pipridae_Passeriformes_Neognathae_Aves_Coelurosauria_Theropoda_Saurischia_Dinosauria_Archosauria_Archelosauria_Euteleostomi_Vertebrata_Craniata_Chordata_Metazoa_Eukaryota KFW74074:0.0359000355)42:0.0004525154)50:0.0044425480,Cyanistes caeruleus @Cyanistes_Paridae_Passeriformes_Neognathae_Aves_Coelurosauria_Theropoda_Saurischia_Dinosauria_Archosauria_Archelosauria_Euteleostomi_Vertebrata_Craniata_Chordata_Metazoa_Eukaryota XP_023784204:0.0085444082)100:0.0271669924)58:0.0088669165,(Columba livia @Columba_Columbidae_Columbiformes_Neognathae_Aves_Coelurosauria_Theropoda_Saurischia_Dinosauria_Archosauria_Archelosauria_Euteleostomi_Vertebrata_Craniata_Chordata_Metazoa_Eukaryota XP_005511099:0.0000027612,Patagioenas fasciata monilis @Patagioenas_Columbidae_Columbiformes_Neognathae_Aves_Coelurosauria_Theropoda_Saurischia_Dinosauria_Archosauria_Archelosauria_Euteleostomi_Vertebrata_Craniata_Chordata_Metazoa_Eukaryota OPJ74063:0.0086820364)100:0.0355591168)59:0.0046765949,(Buceros rhinoceros silvestris @Buceros_Bucerotidae_Bucerotiformes_Neognathae_Aves_Coelurosauria_Theropoda_Saurischia_Dinosauria_Archosauria_Archelosauria_Euteleostomi_Vertebrata_Craniata_Chordata_Metazoa_Eukaryota KFO89341:0.0368773449,Nestor notabilis @Nestor_Psittacidae_Psittaciformes_Neognathae_Aves_Coelurosauria_Theropoda_Saurischia_Dinosauria_Archosauria

_Archelosauria_Euteleostomi_Vertebrata_Craniata_Chordata_Metazoa_Eukaryota
KFQ41818:0.0070359618)99:0.0102983828)97:0.0047657527,(Antrostomus carolinensis
@Antrostomus_Caprimulgidae_Caprimulgiformes_Neognathae_Aves_Coelurosauria_Theropoda_Saurischia_Dinosauria
_Archosauria_Archelosauria_Euteleostomi_Vertebrata_Craniata_Chordata_Metazoa_Eukaryota
KFZ50498:0.0080311418,Egretta garzetta
@Egretta_Ardeidae_Pelecaniformes_Neognathae_Aves_Coelurosauria_Theropoda_Saurischia_Dinosauria_Archosauria_
Archelosauria_Euteleostomi_Vertebrata_Craniata_Chordata_Metazoa_Eukaryota
KFP14470:0.0134170787)92:0.0085840526)92:0.0037929531,(Haliaeetus albicilla
@Haliaeetus_Accipitrinae_Accipitridae_Falconiformes_Neognathae_Aves_Coelurosauria_Theropoda_Saurischia_Dinosa
uria_Archosauria_Archelosauria_Euteleostomi_Vertebrata_Craniata_Chordata_Metazoa_Eukaryota
KFQ09747:0.0042457198,Leptosomus discolor
@Leptosomus_Leptosomidae_Coraciiformes_Neognathae_Aves_Coelurosauria_Theropoda_Saurischia_Dinosauria_Arch
osauria_Archelosauria_Euteleostomi_Vertebrata_Craniata_Chordata_Metazoa_Eukaryota
KFQ13842:0.0128297177)87:0.0000029698)95:0.0000024625,Eurypyga helias
@Eurypyga_Eurypygidae_Gruiformes_Neognathae_Aves_Coelurosauria_Theropoda_Saurischia_Dinosauria_Archosauria
_Archelosauria_Euteleostomi_Vertebrata_Craniata_Chordata_Metazoa_Eukaryota
KFV95075:0.0042851582)94:0.0042617550,(((Aptenodytes forsteri
@Aptenodytes_Spheniscidae_Sphenisciformes_Neognathae_Aves_Coelurosauria_Theropoda_Saurischia_Dinosauria_Ar
chosauria_Archelosauria_Euteleostomi_Vertebrata_Craniata_Chordata_Metazoa_Eukaryota
KFM01548:0.0000023247,Pygoscelis adeliae
@Pygoscelis_Spheniscidae_Sphenisciformes_Neognathae_Aves_Coelurosauria_Theropoda_Saurischia_Dinosauria_Arch
osauria_Archelosauria_Euteleostomi_Vertebrata_Craniata_Chordata_Metazoa_Eukaryota
KFW62695:0.0000023247)100:0.0085812396,Balearica regulorum gibbericeps
@Balearica_Gruidae_Gruiformes_Neognathae_Aves_Coelurosauria_Theropoda_Saurischia_Dinosauria_Archosauria_Arc
helosauria_Euteleostomi_Vertebrata_Craniata_Chordata_Metazoa_Eukaryota
KFO10016:0.0000022151)96:0.0042496764,(Fulmarus glacialis
@Fulmarus_Procellariidae_Procellariiformes_Neognathae_Aves_Coelurosauria_Theropoda_Saurischia_Dinosauria_Arch
osauria_Archelosauria_Euteleostomi_Vertebrata_Craniata_Chordata_Metazoa_Eukaryota
KFV90292:0.0400369529,Podiceps cristatus
@Podiceps_Podicipedidae_Podicipediformes_Neognathae_Aves_Coelurosauria_Theropoda_Saurischia_Dinosauria_Arc
hosauria_Archelosauria_Euteleostomi_Vertebrata_Craniata_Chordata_Metazoa_Eukaryota
KFZ69041:0.0085341006)79:0.0000021381)71:0.0000022662)97:0.0042534196,Nipponia nippon
@Nipponia_Threskiornithidae_Pelecaniformes_Neognathae_Aves_Coelurosauria_Theropoda_Saurischia_Dinosauria_Ar
chosauria_Archelosauria_Euteleostomi_Vertebrata_Craniata_Chordata_Metazoa_Eukaryota
KFQ94150:0.0085964217)97:0.0079036046,(Gallus gallus
@Gallus_Phasianinae_Phasianidae_Galliformes_Galloanserae_Neognathae_Aves_Coelurosauria_Theropoda_Saurischia
_Dinosauria_Archosauria_Archelosauria_Euteleostomi_Vertebrata_Craniata_Chordata_Metazoa_Eukaryota
NP_001026386:0.0121294910,Numida meleagris
@Numida_Numididae_Galliformes_Galloanserae_Neognathae_Aves_Coelurosauria_Theropoda_Saurischia_Dinosauria_
Archosauria_Archelosauria_Euteleostomi_Vertebrata_Craniata_Chordata_Metazoa_Eukaryota
XP_021258124:0.0136434621)100:0.0407287842)100:0.0415305027,Pelodiscus sinensis
@Pelodiscus_Trionychidae_Trionychia_Cryptodira_Testudines_Archelosauria_Euteleostomi_Vertebrata_Craniata_Chord
ata_Metazoa_Eukaryota NP_001273863:0.0774860047)61:0.0256744834,((Carassius auratus ssp. Pengze
@Carassius_Cyprinidae_Cypriniformes_Ostariophysi_Teleostei_Neopterygii_Actinopterygii_Euteleostomi_Vertebrata_C
raniata_Chordata_Metazoa_Eukaryota AGC50801:0.1365754981,Protopterus annectens
@Protopterus_Protopteridae_Lepidosireniformes_Dipnoi_Euteleostomi_Vertebrata_Craniata_Chordata_Metazoa_Euka
ryota AGV06209:0.1059244432)93:0.0190611740,Xenopus laevis
@Xenopus_Xenopus_Xenopodinae_Pipidae_Pipoidea_Anura_Batrachia_Amphibia_Euteleostomi_Vertebrata_Craniata_
Chordata_Metazoa_Eukaryota ABK62836:0.1605094732)56:0.0115694065)66:0.0046142503,(((Bos taurus

@Bos_Bovinae_Bovidae_Pecora_Ruminantia_Cetartiodactyla_Laurasiatheria_Eutheria_Mammalia_Euteleostomi_Vertebrata_Craniata_Chordata_Metazoa_Eukaryota NP_001030463:0.0459219762,(Homo sapiens @Homo_Hominidae_Catarrhini_Haplorrhini_Primates_Euarchontoglires_Eutheria_Mammalia_Euteleostomi_Vertebrata_Craniata_Chordata_Metazoa_Eukaryota AAK29181:0.0494848687,Phascolarctos cinereus @Phascolarctos_Phascolarctidae_Diprotodontia_Metatheria_Mammalia_Euteleostomi_Vertebrata_Craniata_Chordata_Metazoa_Eukaryota XP_020819557:0.0874653434)96:0.0307006955)93:0.0191192270,Felis catus @Felis_Felinae_Felidae_Feliformia_Carnivora_Laurasiatheria_Eutheria_Mammalia_Euteleostomi_Vertebrata_Craniata_Chordata_Metazoa_Eukaryota XP_003993206:0.0212681759)92:0.0294332857,Mus musculus @Mus_Mus_Murinae_Muridae_Muroidea_Myomorpha_Rodentia_Glires_Euarchontoglires_Eutheria_Mammalia_Euteleostomi_Vertebrata_Craniata_Chordata_Metazoa_Eukaryota NP_033934:0.0270298357)94:0.0320380781)100:0.2264771956,(Penaeus monodon @Penaeus_Penaeidae_Penaeoidea_Dendrobranchiata_Decapoda_Eucarida_Eumalacostraca_Malacostraca_Crustacea_Arthropoda_Ecdysozoa_Metazoa_Eukaryota ALM09355:0.0657539503,Portunus trituberculatus @Portunus_Portunidae_Portunoidea_Eubrachyura_Brachyura_Pleocyemata_Decapoda_Eucarida_Eumalacostraca_Malacostraca_Crustacea_Arthropoda_Ecdysozoa_Metazoa_Eukaryota ACI13850:0.1275517281)100:0.0959915592)54:0.0213264959,(((((Bombyx mori @Bombyx_Bombycinae_Bombycidae_Bombycoidea_Ditrysia_Glossata_Lepidoptera_Holometabola_Neoptera_Pterygota_Insecta_Hexapoda_Arthropoda_Ecdysozoa_Metazoa_Eukaryota NP_001036912:0.2792061881,Drosophila obscura @Sophophora_Drosophila_Drosophilidae_Ephydroidea_Muscomorpha_Brachycera_Diptera_Holometabola_Neoptera_Pterygota_Insecta_Hexapoda_Arthropoda_Ecdysozoa_Metazoa_Eukaryota XP_022221427:0.2185664817)100:0.1229501923,Camponotus floridanus @Camponotus_Formicinae_Formicidae_Vespoidea_Aculeata_Apocrita_Hymenoptera_Holometabola_Neoptera_Pterygota_Insecta_Hexapoda_Arthropoda_Ecdysozoa_Metazoa_Eukaryota EFN66292:0.2297158758)95:0.0390271198,Locusta migratoria manilensis @Locusta_Oedipodinae_Acrididae_Acridoidea_Acridomorpha_Acrididea_Caelifera_Orthoptera_Polyneoptera_Neoptera_Pterygota_Insecta_Hexapoda_Arthropoda_Ecdysozoa_Metazoa_Eukaryota AOG21082:0.1553260651)99:0.0427783803,Toxocara canis @Toxocara_Toxocaridae_Ascaridoidea_Ascaridida_Chromadorea_Nematoda_Ecdysozoa_Metazoa_Eukaryota KHN75905:0.5119322441)94:0.0596994182,((Crassostrea hongkongensis @Crassostrea_Ostreidae_Ostreoidea_Ostreoida_Pteriomorphia_Bivalvia_Mollusca_Lophotrochozoa_Metazoa_Eukaryota ADZ76134:0.2174859959,Octopus vulgaris @Octopus_Octopodidae_Incirrata_Octopoda_Octopodiformes_Neocoleoidea_Coleoidea_Cephalopoda_Mollusca_Lophotrochozoa_Metazoa_Eukaryota AGZ63441:0.1903649015)100:0.0596777073,(Eisenia fetida @Eisenia_Lumbricinae_Lumbricidae_Lumbricina_Haplotaxida_Oligochaeta_Clitellata_Annelida_Lophotrochozoa_Metazoa_Eukaryota AEO50756:0.3872004363,Perinereis aibuhitensis @Perinereis_Nereididae_Phyllodocida_Aciculata_Palpata_Polychaeta_Annelida_Lophotrochozoa_Metazoa_Eukaryota AHY86343:0.2436466822)95:0.0660521646)84:0.0323340553)91:0.0456898981)96:0.0981836638,((((Aphanomyces astaci @Aphanomyces_Saprolegniaceae_Saprolegniales_Oomycetes_Stramenopiles_Eukaryota RHZ15061:0.1339938022,(Achlya hypogyna @Achlya_Saprolegniaceae_Saprolegniales_Oomycetes_Stramenopiles_Eukaryota OQR84492:0.0461407583,Saprolegnia diclina VS20 @Saprolegnia_Saprolegniaceae_Saprolegniales_Oomycetes_Stramenopiles_Eukaryota XP_008617088:0.0738481678)100:0.0776856540)100:0.1463797862,((Phytophthora parasitica P1569 @Phytophthora_Peronosporales_Oomycetes_Stramenopiles_Eukaryota ETI55580:0.0966476684,Phytophthora sojae @Phytophthora_Peronosporales_Oomycetes_Stramenopiles_Eukaryota XP_009527353:0.1654292377)56:0.0448577348,Pythium oligandrum @Pythium_Pythiaceae_Pythiales_Oomycetes_Stramenopiles_Eukaryota TMW58298:0.2172462929)100:0.0644063198)100:0.1361711470,Fistulifera solaris @Fistulifera_Naviculaceae_Naviculales_Bacillariophycidae_Bacillariophyceae_Bacillariophyta_Stramenopiles_Eukaryota GAX17996:0.3682953009)100:0.0440983945,(Nannochloropsis gaditana

@Nannochloropsis_Monodopsidaceae_Eustigmatales_Eustigmatophyceae_Stramenopiles_Eukaryota EWM20937:0.0082267766,Nannochloropsis salina CCMP1776 @Microchloropsis salina_Microchloropsis_Monodopsidaceae_Eustigmatales_Eustigmatophyceae_Stramenopiles_Eukaryota TFJ82773:0.0087639525)100:0.1999472930)100:0.0807459434)100:0.3243746265,((Basidiobolus meristosporus CBS 931.73 @Basidiobolus_Basidiobolaceae_Basidiobolales_Basidiobolomycetes_Entomophthoromycotina_Zoopagomycota_Fungi_Eukaryota ORY01796:0.1561755504,(Mucor ambiguus @Mucor_Mucoraceae_Mucorineae_Mucorales_Mucoromycotina_Mucoromycota_Fungi_Eukaryota GAN01536:0.0733179637,Rhizopus microsporus @Rhizopus_Rhizopodaceae_Mucorineae_Mucorales_Mucoromycotina_Mucoromycota_Fungi_Eukaryota CEG67568:0.0698658425)100:0.1183312210)100:0.1966430474,(((Oxytricha trifallax @Oxytricha_Oxytrichinae_Oxytrichidae_Sporadotrichida_Stichotrichia_Spirotrichea_Intramacronucleata_Ciliophora_Alveolata_Eukaryota EJY78794:0.0840639828,Stylonychia lemnae @Stylonychia_Stylonychinae_Oxytrichidae_Sporadotrichida_Stichotrichia_Spirotrichea_Intramacronucleata_Ciliophora_Alveolata_Eukaryota CDW81170:0.1375539831)100:0.3409798474,((Tetrahymena thermophila SB210 @Tetrahymena_Tetrahymenidae_Tetrahymenina_Hymenostomatida_Oligohymenophorea_Intramacronucleata_Ciliophora_Alveolata_Eukaryota XP_001026590:0.1611247828,Paramecium tetraurelia strain d4-2 @Paramecium_Parameciidae_Peniculida_Oligohymenophorea_Intramacronucleata_Ciliophora_Alveolata_Eukaryota XP_001450296:0.2566902175)63:0.0671894324,Plasmodiophora brassicae @Plasmodiophora_Plasmodiophoridae_Plasmodiophorida_Imbricatea_Cercozoa_Rhizaria_Eukaryota CEO97147:0.4521296198)84:0.0588709453)99:0.0543269031,(((Toxoplasma gondii ME49 @Toxoplasma_Sarcocystidae_Eimeriorina_Eucoccidiorida_Coccidia_Conoidasida_Apicomplexa_Alveolata_Eukaryota XP_002368095:0.0000023268,Hammondia hammondi @Hammondia_Sarcocystidae_Eimeriorina_Eucoccidiorida_Coccidia_Conoidasida_Apicomplexa_Alveolata_Eukaryota KEP63689:0.0084064801)100:0.0349076326,Neospora caninum Liverpool @Neospora_Sarcocystidae_Eimeriorina_Eucoccidiorida_Coccidia_Conoidasida_Apicomplexa_Alveolata_Eukaryota XP_003883469:0.0781026336)100:0.3582475588,Vitrella brassicaformis @Vitrella_Chromerida_Alveolata_Eukaryota AND95675:0.1972696722)100:0.1629941855)86:0.0614465668)64:0.0364366294)53:0.0340927246,(Acanthamoeba castellanii @Acanthamoeba_Acanthamoebidae_Centramoebida_Longamoebia_Discosea_Amoebozoa_Eukaryota XP_004337981:0.2974592797,((Dictyostelium discoideum @Dictyostelium_Dictyosteliida_Mycetozoa_Amoebozoa_Eukaryota AAC36743:0.1293274778,(Acytostelium subglobosum LB1 @Acytostelium_Dictyosteliida_Mycetozoa_Amoebozoa_Eukaryota XP_012758538:0.0663382908,Cavenderia fasciculata @Cavenderia_Cavenderiaceae_Acytosteliales_Dictyostelids_Mycetozoa_Amoebozoa_Eukaryota XP_004355480:0.0886457860)100:0.0208319273)91:0.0274998385,Tieghemostelium lacteum @Tieghemostelium_Raperosteliaceae_Dictyosteliales_Dictyostelids_Mycetozoa_Amoebozoa_Eukaryota AGC69744:0.0821501099)100:0.1658421897)100:0.1024952410)93:0.0616302915)100:0.6901944555,((Pyropia haitanensis @Pyropia_Bangiaceae_Bangiales_Bangiophyceae_Rhodophyta_Eukaryota AJA71274:0.1233947745,Pyropia yezoensis @Pyropia_Bangiaceae_Bangiales_Bangiophyceae_Rhodophyta_Eukaryota ADO23652:0.0785126040)100:0.0729864074,Porphyra umbilicalis @Porphyra_Bangiaceae_Bangiales_Bangiophyceae_Rhodophyta_Eukaryota OSX73109:0.2768928258)100:1.3097377070)100:0.3615151100,((((((((((Bassia scoparia @Bassia_Camphorosmeae_Camphorosmoideae_Chenopodiaceae_Caryophyllales_Pentapetalae_Gunneridae_eudicotyledons_Magnoliophyta_Spermatophyta_Tracheophyta_Embryophyta_Streptophyta_Viridiplantae_Eukaryota AIP93616:0.0308248542,Beta vulgaris subsp. vulgaris @Beta_Betoideae_Chenopodiaceae_Caryophyllales_Pentapetalae_Gunneridae_eudicotyledons_Magnoliophyta_Spermatophyta_Tracheophyta_Embryophyta_Streptophyta_Viridiplantae_Eukaryota XP_010692546:0.0421971660)92:0.0128064383,Spinacia oleracea @Spinacia_Anserineae_Chenopodioideae_Chenopodiaceae_Caryophyllales_Pentapetalae_Gunneridae_eudicotyledons_

Magnoliophyta_Spermatophyta_Tracheophyta_Embryophyta_Streptophyta_Viridiplantae_Eukaryota XP_021845231:0.0141728492)99:0.0373520844,Mesembryanthemum crystallinum @Cryophytum_Mesembryanthemum_Aizoaceae_Caryophyllales_Pentapetalae_Gunneridae_eudicotyledons_Magnoliophyta_Spermatophyta_Tracheophyta_Embryophyta_Streptophyta_Viridiplantae_Eukaryota AAC19397:0.0524467824)93:0.0354835282,((Brachypodium distachyon @Brachypodium_Brachypodieae_Pooideae_BOP clade_Poaceae_Poales_Liliopsida_Magnoliophyta_Spermatophyta_Tracheophyta_Embryophyta_Streptophyta_Viridiplantae_Eukaryota XP_003558892:0.0076161350,(Triticum aestivum @Triticum_Triticinae_Triticeae_Triticodae_Pooideae_BOP clade_Poaceae_Poales_Liliopsida_Magnoliophyta_Spermatophyta_Tracheophyta_Embryophyta_Streptophyta_Viridiplantae_Eukaryota AIZ77475:0.0000029032,Triticum aestivum @Triticum_Triticinae_Triticeae_Triticodae_Pooideae_BOP clade_Poaceae_Poales_Liliopsida_Magnoliopsida_Spermatophyta_Tracheophyta_Embryophyta_Streptophyta_Viridiplantae_Eukaryota Q43206:0.0042548308)100:0.0648797926)100:0.0342981937,(Oryza brachyantha @Oryza_Oryzinae_Oryzeae_Oryzoideae_BOP clade_Poaceae_Poales_Liliopsida_Magnoliophyta_Spermatophyta_Tracheophyta_Embryophyta_Streptophyta_Viridiplantae_Eukaryota XP_006649332:0.0000023444,Oryza sativa Japonica Group @Oryza_Oryzinae_Oryzeae_Oryzoideae_BOP clade_Poaceae_Poales_Liliopsida_Magnoliophyta_Spermatophyta_Tracheophyta_Embryophyta_Streptophyta_Viridiplantae_Eukaryota XP_015629749:0.0251132815)100:0.0028779593)100:0.0863188496)91:0.0244233219,((Glycine max @Soja_Glycine_Phaseoleae_indigoferoid/millettioid clade_NPAAA clade_50 kb inversion clade_Papilionoideae_Fabaceae_Fabales_fabids_rosids_Pentapetalae_Gunneridae_eudicotyledons_Magnoliopsida_Spermatophyta_Tracheophyta_Embryophyta_Streptophyta_Viridiplantae_Eukaryota NP_001237556:0.0170038778,Glycine max @Soja_Glycine_Phaseoleae_indigoferoid/millettioid clade_NPAAA clade_50 kb inversion clade_Papilionoideae_Fabaceae_Fabales_fabids_rosids_Pentapetalae_Gunneridae_eudicotyledons_Magnoliopsida_Spermatophyta_Tracheophyta_Embryophyta_Streptophyta_Viridiplantae_Eukaryota NP_001235974:0.0042145431)100:0.0412085640,Glycine max @Soja_Glycine_Phaseoleae_indigoferoid/millettioid clade_NPAAA clade_50 kb inversion clade_Papilionoideae_Fabaceae_Fabales_fabids_rosids_Pentapetalae_Gunneridae_eudicotyledons_Magnoliopsida_Spermatophyta_Tracheophyta_Embryophyta_Streptophyta_Viridiplantae_Eukaryota NP_001237571:0.0762774642)100:0.0294582179)94:0.0333081758,(((Brassica oleracea var. oleracea @Brassica_Brassiceae_Brassicaceae_Brassicales_malvids_rosids_Pentapetalae_Gunneridae_eudicotyledons_Magnoliophyta_Spermatophyta_Tracheophyta_Embryophyta_Streptophyta_Viridiplantae_Eukaryota XP_013597488:0.0000023247,Brassica napus @Brassica_Brassiceae_Brassicaceae_Brassicales_malvids_rosids_Pentapetalae_Gunneridae_eudicotyledons_Magnoliophyta_Spermatophyta_Tracheophyta_Embryophyta_Streptophyta_Viridiplantae_Eukaryota XP_013704129:0.0041645941)100:0.0045501088,Brassica rapa @Brassica_Brassiceae_Brassicaceae_Brassicales_malvids_rosids_Pentapetalae_Gunneridae_eudicotyledons_Magnoliophyta_Spermatophyta_Tracheophyta_Embryophyta_Streptophyta_Viridiplantae_Eukaryota VDC97981:0.0038218795)100:0.0128731985,(Arabidopsis thaliana @Arabidopsis_Camelineae_Brassicaceae_Brassicales_malvids_rosids_Pentapetalae_Gunneridae_eudicotyledons_Magnoliophyta_Spermatophyta_Tracheophyta_Embryophyta_Streptophyta_Viridiplantae_Eukaryota NP_564121:0.0037199337,Arabidopsis lyrata subsp. lyrata @Arabidopsis_Camelineae_Brassicaceae_Brassicales_malvids_rosids_Pentapetalae_Gunneridae_eudicotyledons_Magnoliophyta_Spermatophyta_Tracheophyta_Embryophyta_Streptophyta_Viridiplantae_Eukaryota XP_020866686:0.0087703340)100:0.0356611437)100:0.0763191757)100:0.1184887774,((Auxenochlorella protothecoides @Auxenochlorella_Chlorellaceae_Chlorellales_Trebouxiophyceae_Chlorophyta_Viridiplantae_Eukaryota XP_011396830:0.3664369474,Chlorella sorokiniana @Chlorella_Chlorellaceae_Chlorellales_Trebouxiophyceae_Chlorophyta_Viridiplantae_Eukaryota PRW57738:0.1185992698)83:0.0327381903,Coccomyxa subellipsoidea C-169

@Coccomyxa_Coccomyxaceae_Trebouxiophyceae incertae
sedis_Trebouxiophyceae_Chlorophyta_Viridiplantae_Eukaryota
XP_005645077:0.1299473810)76:0.0170090578)93:0.1308224812,Ulva prolifera
@Ulva_Ulvaceae_Ulvales_Ulvophyceae_Chlorophyta_Viridiplantae_Eukaryota
AKA94107:0.1917208931)53:0.0332916894,((Chlamydomonas reinhardtii
@Chlamydomonas_Chlamydomonadaceae_Chlamydomonadales_Chlorophyceae_Chlorophyta_Viridiplantae_Eukaryota
XP_001696763:0.0382687025,Gonium pectorale
@Gonium_Volvocaceae_Chlamydomonadales_Chlorophyceae_Chlorophyta_Viridiplantae_Eukaryota
KXZ42175:0.0375302086)100:0.0170043992,Volvox carteri f. nagariensis
@Volvox_Volvocaceae_Chlamydomonadales_Chlorophyceae_Chlorophyta_Viridiplantae_Eukaryota
XP_002950455:0.0293213789)100:0.1069165101)100:0.2366222472,Galdieria sulphuraria
@Galdieria_Cyanidiaceae_Cyanidiales_Bangiophyceae_Rhodophyta_Eukaryota
XP_005708279:0.3142130702)97:0.1269010294,Cyanidioschyzon merolae strain 10D
@Cyanidioschyzon_Cyanidiaceae_Cyanidiales_Bangiophyceae_Rhodophyta_Eukaryota
XP_005536240:0.4106928932)98:0.1275135482,(Gracilariopsis chorda
@Gracilariopsis_Gracilariaceae_Gracilariales_Rhodymeniophycidae_Florideophyceae_Rhodophyta_Eukaryota
PXF41280:0.0805346893,Chondrus crispus
@Chondrus_Gigartinaceae_Gigartinales_Rhodymeniophycidae_Florideophyceae_Rhodophyta_Eukaryota
XP_005712277:0.1142702163)100:0.5705163940)100:0.2412167763)100:0.5780204087,(Capsaspora owczarzaki ATCC
30864 @Capsaspora_Ichthyosporea_Eukaryota XP_004366029:0.0000023575,Capsaspora owczarzaki ATCC 30864
@Capsaspora_Ichthyosporea_Eukaryota
KJE89728:0.0135875883)100:0.3916137017)99:0.2283105084)100:0.2424705610,((Artemidia motanka
1610_TRINITY_DN17294_c8_g5_i1:0.0893553446,((Namystynia karyoxenos
1621_TRINITY_DN46753_c0_g1_i1:0.0000025789,(Namystynia karyoxenos
1621_TRINITY_DN46753_c0_g1_i3:0.0164221334,Namystynia karyoxenos
1621_TRINITY_DN46753_c0_g1_i4:0.0000020027)89:0.0220656348)85:0.0140495432,Namystynia karyoxenos
1621_TRINITY_DN46753_c0_g1_i2:0.0114415135)100:0.0588496080)100:0.0715317049,Artemidia motanka
1610_TRINITY_DN17294_c8_g7_i1:0.0872784928)100:0.0633324660)97:0.0513058525)100:0.0865857645);
